# DRIME: A Distributed Data-Guided RIME Algorithm for Numerical Optimization Problems

**DOI:** 10.3390/biomimetics10090589

**Published:** 2025-09-03

**Authors:** Jinghao Yang, Yuanyuan Shao, Bin Fu, Lei Kou

**Affiliations:** 1 Metropolitan College, Boston University, Boston, MA 02215, USA; yjh0830@bu.edu; 2 Taizhou Institute, Zhejiang University, Taizhou 318000, China; shaoyuanyuan@tzizju.cn (Y.S.); fubin@tzizju.cn (B.F.); 3 School of Qilu Transportation, Shandong University, Jinan 250061, China

**Keywords:** RIME, metaheuristic algorithms, guided learning strategy, candidate pool, CEC test suite

## Abstract

To address the shortcomings of the RIME algorithm’s weak global exploration ability, insufficient information exchange among populations, and limited population diversity, this work proposes a distributed data-guided RIME algorithm called DRIME. First, this paper proposes a data-distribution-driven guided learning strategy that enhances information exchange among populations and dynamically guides populations to exploit or explore. Then, a soft-rime search phase based on weighted averaging is proposed, which balances the development and exploration of RIME by alternating with the original strategy. Finally, a candidate pool is utilized to replace the optimal reference point of the hard-rime puncture mechanism to enrich the diversity of the population and reduce the risk of falling into local optima. To evaluate the performance of the DRIME algorithm, parameter sensitivity analysis, policy effectiveness analysis, and two comparative analyses are performed on the CEC-2017 test set and the CEC-2022 test set. The parameter sensitivity analysis identifies the optimal parameter settings for the DRIME algorithm. The strategy effectiveness analysis confirms the effectiveness of the improved strategies. In comparison with ACGRIME, TERIME, IRIME, DNMRIME, GLSRIME, and HERIME on the CEC-2017 test set, the DRIME algorithm achieves Friedman rankings of 1.517, 1.069, 1.138, and 1.069 in different dimensions. In comparison with EOSMA, GLS-MPA, ISGTOA, EMTLBO, LSHADE-SPACMA, and APSM-jSO on the CEC-2022 test set, the DRIME algorithm achieves Friedman rankings of 2.167 and 1.917 in 10 and 30 dimensions, respectively. In addition, the DRIME algorithm achieved an average ranking of 1.23 in engineering constraint optimization problems, far surpassing other comparison algorithms. In conclusion, the numerical optimization experiments successfully illustrate that the DRIME algorithm has excellent search capability and can provide satisfactory solutions to a wide range of optimization problems.

## 1. Introduction

Optimization problems are common in many fields, and their purpose is to find the values of variables and related solutions that make the objective function reach the maximum or minimum value under the premise of satisfying certain constraints [1]. With the rapid development of science and technology and the deepening of research in various fields, optimization problems are becoming more and more complex, showing multimodal, nonlinear, discontinuous, and high-dimensional characteristics, and the constraints are becoming more and more cumbersome [2,3]. For example, in a large-scale production scheduling problem, it is necessary to consider the mutual influence of many productions’ equipment, process flow, resource allocation, and other factors [4]. In the complex logistics and distribution network optimization, it is necessary to take into account the traffic conditions, transportation cost, customer demand, and other factors [5]. In a UAV path planning problem, path length, path height, turning radius, degree of co-ordination, and other factors need to be considered simultaneously [6]. These complex problems are extremely difficult to solve and pose a serious challenge to the performance and efficiency of optimization algorithms [7].

Traditional optimization methods mainly include deterministic algorithms such as linear programming, nonlinear programming, dynamic programming, and integer programming. Deterministic algorithms perform well in dealing with linear, microscopic, continuous, low-dimensional, and simple optimization problems, providing exact optimal solutions. Like the simplex method in linear programming, it is widely used in linear problems such as resource allocation [8] and production planning [9]. Dynamic programming, on the other hand, is suitable for problems with overlapping subproblems and optimal substructures, such as the shortest path problem [10]. However, the frequent occurrence of complex optimization issues exposes the limitations of traditional deterministic approaches. It is highly inefficient or perhaps impossible to solve when dealing with large-scale complex optimization issues because of its reliance on gradient information, which makes it very easy to fall into local optimization, and the computational volume grows exponentially with the problem scale [11].

The metaheuristic algorithm is an efficient stochastic search algorithm, which provides a suitable solution to the optimization problem by performing random search in the problem solution space and by using stochastic operators and trial-and-error process, with both exploratory and exploitative capabilities; it can strike a balance between global and local search, thus effectively avoiding falling into the local optimum [12]. Compared with traditional methods, its advantage is that it does not need to set too many limits on the characteristics of the problem, and its adaptability is super strong [13]. Nowadays, it shines in many fields such as mechanical engineering [14,15], mission planning [16,17], electric power systems [18,19], biomedicine [20,21,22], large-scale optimization problems [23,24,25], expensive optimization problems [26,27,28], etc., helping to optimize the design parameters of parts and components, the scheduling of generator sets, the accuracy of disease diagnosis, and other key links. Based on the background and design ideas of the proposed metaheuristic algorithms, the three main categories are evolution-based algorithms, swarm-based algorithms, and physics-based algorithms.

Evolution-based algorithms draw on the evolutionary process of organisms and evolutionary theory as the source of inspiration. Among them, the Genetic Algorithm (GA) [29] continuously promotes population evolution by modeling natural selection and genetic mechanisms with the help of selection, crossover, mutation, and other operations, and eventually converges to the global optimal solution. Differential Evolution (DE) [30], on the other hand, searches for the optimal solution efficiently in the solution space by performing mutation operations through the difference vectors between individuals. In recent years, evolution-based algorithms have been emerging, such as Evolutionary Strategies (ES) [31], Genetic Programming (GP) [32], Alpha Evolution (AE) [33], which show good adaptability and optimization performance in different types of optimization problems, and provide new ideas and methods for solving complex optimization problems.

Swarm-based algorithms were developed inspired by the collaborative behavior of groups of organisms in nature. These algorithms search for optimal solutions by modeling the collaboration and information exchange between individuals in a group. These algorithms efficiently search for optimal solutions in the solution space by modeling the collaboration and information-sharing mechanism among individuals in a group. For example, Particle Swarm Optimization (PSO) [34] simulates the behavior of a flock of birds foraging for food, in which each particle dynamically adjusts its moving direction and speed according to its own experience, and the optimal experience of the group, to quickly converge to the global optimal solution, which is a simple algorithm with rapid convergence. Ant Colony Optimization (ACO) [35] simulates the communication of ants by releasing pheromones during the foraging process so as to find the shortest path from the nest to the food source, which demonstrates the powerful ability of group intelligence in the path optimization problem. In recent years, the research and development of swarm intelligence algorithms have received widespread attention, and numerous novel algorithms have emerged. These algorithms include, but are not limited to, the Superb Fairy-wren Optimization Algorithm (SFOA) [36], Hawk Fish Optimization Algorithm (HFOA) [37], Artificial Lemming Algorithm (ALA) [38], Sled Dog Optimizer (SDO) [39], Chinese Pangolin Optimizer (CPO) [40], Snow Geese Algorithm (SGA) [41], Starfish Optimization Algorithm (SOA) [42], Frilled Lizard Optimization (FLO) [43], Arctic Puffin Optimization (APO) [44], Eel and Grouper Optimizer (EGO) [45], Greater Cane Rat Algorithm (GCRA) [46], and the Secretary Bird Optimization Algorithm (SBOA) [47]. These new methods have demonstrated unique performance and advantages in different optimization problems, further expanding the application prospects of population intelligence algorithms in complex optimization problems.

Physics-based algorithms draw inspiration from the physical or chemical laws of nature and efficiently solve complex problems by simulating these natural processes. Simulated Annealing (SA) [48] is modeled on the annealing process of crystalline solids, and introduces a temperature parameter to regulate the probability of solution acceptance during the search process. The algorithm allows accepting inferior solutions with a certain probability, thus effectively getting rid of the constraints of local optimum and realizing global optimization. The Gravitational Search Algorithm (GSA) [49] is based on the law of gravity, which treats the solutions as objects with mass, and the objects interact with each other through gravitational force. The objects with larger mass represent better solutions, and the search direction is guided by the gravitational force to push the algorithm to approach the global optimal solution step by step. In recent years, optimization algorithms based on physical phenomena have emerged, such as the Planet Optimization Algorithm (POA) [50], Fata Morgana Algorithm (FMA) [51], Attraction Repulsion Optimization Algorithm (AROA) [52], Newton–Raphson-Based Optimizer (NRBO) [53], Snow Ablation Optimizer (SAO) [54], and the Fick’s Law Algorithm (FLA) [55]. These algorithms provide new ideas and methods for solving complex optimization problems by virtue of their unique physical mechanisms and efficient search capabilities, and show broad application potential.

In addition to the above three classes of classical metaheuristic algorithms, two emerging classes of optimization algorithms have emerged in recent years. One class of algorithms is based on human behaviors or cognitive processes, represented by Teaching–Learning-Based Optimization (TLBO) [56], which simulates the process of teaching and learning interactions between teachers and students. In addition, such algorithms include the Connected Banking System Optimizer (CBSO) [57], Catch Fish Optimization Algorithm (CFOA) [58], Child Drawing Development Optimization (CDDO) [59], Mountaineering Team-Based Optimization (MTBO) [60], Preschool Education Optimization Algorithm (PEOA) [61], and the Human Evolutionary Optimization Algorithm (HEOA) [62]. These algorithms provide new perspectives for solving complex optimization problems by imitating human learning, communication, and decision-making mechanisms.

**Figure 1:**
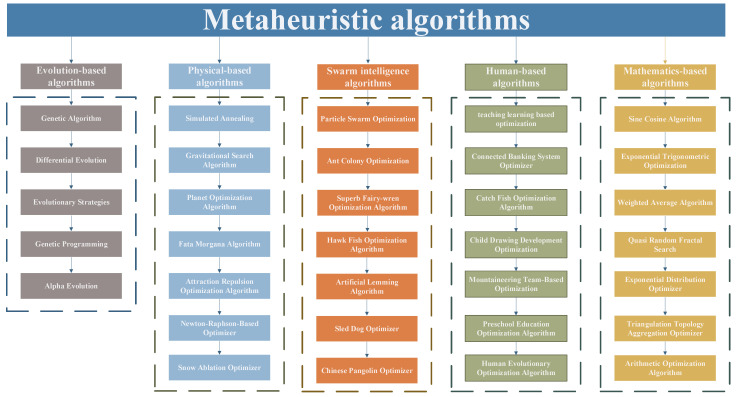
Summary of metaheuristic algorithms.

Another class of algorithms is based on mathematical theories or mathematical models, represented by the Sine Cosine Algorithm (SCA) [63], which utilizes the periodic and oscillatory properties of the sine and cosine functions to guide the search process. Such algorithms also include Exponential Trigonometric Optimization (ETO) [64], the Weighted Average Algorithm (WAA) [65], Quasi Random Fractal Search (QRFS) [66], Exponential Distribution Optimizer (EDO) [67], Triangulation Topology Aggregation Optimizer (TTAO) [68], and the Arithmetic Optimization Algorithm (AOA) [69]. These algorithms provide efficient and accurate solutions to optimization problems by leveraging the power of expression and the regularity of mathematical tools. These five categories of metaheuristic algorithms are summarized in Figure 1.

The RIME algorithm is a metaheuristic optimization algorithm based on physical phenomena, which was developed by Su et al. [70]. It is inspired by the growth patterns of soft and hard mistletoe. With its excellent optimization performance, the RIME algorithm has been widely used in several fields. However, according to the No Free Lunch (NFL) theory, no single optimization method can perform well on all problems, and each algorithm has its advantages and limitations under specific problem types and conditions. Therefore, in order to further improve the performance of the RIME algorithm, some scholars have enhanced and optimized it. Li et al. proposed a roulette-based fitness–distance-balancing mechanism for the hard search phase with the aim of balancing the development and exploration of the RIME algorithm [71]. Zhou et al. enriched the population diversity of the RIME algorithm by incorporating a shared information mechanism, and solved the photovoltaic model parameter identification problem [72]. Starting from population initialization and boundary control approaches, Xie et al. proposed an elite reverse learning population selection strategy and a positive cosine boundary control strategy [73]. Wang et al. proposed an improved RIME algorithm for the feature selection problem by combining a chaotic local search strategy and interaction mechanism [74]. Zhong et al. divided the RIME population into three subpopulations based on fitness values and used different search strategies for each of them to address their shortcomings in terms of premature convergence [75]. With the aim of improving the accuracy of medical image segmentation, Sun et al. proposed an improved RIME algorithm incorporating a dual-enhanced solution quality mechanism [76]. Wu et al. enhanced the ability of the RIME algorithm to jump out of the local optimum by combining the double decay mechanism and the simplex method [77]. Gu et al. proposed a crystalline diffusion strategy, a high-altitude coalescence strategy, and a lattice preparation strategy to boost the development and exploration of the RIME algorithm in a comprehensive manner [78]. Aljaidi et al. developed a multi-objective variant of RIME and applied it to the weight- and compliance-based optimization of eight truss designs [79]. Pandya et al. employed non-dominated sorting and crowding distance mechanisms to devise a multi-objective RIME algorithm, and successfully addressed a hybrid power system optimization problem [80]. Yu et al. used reinforcement learning to dynamically select search strategies, achieving a balance between exploitation and exploration [81].

Even though the aforementioned studies partially address RIME’s drawbacks and use these variations for optimization problems across a range of domains, RIME still has drawbacks, including a poor global search capability, ineffective population-to-population information sharing, and an imbalance between exploitation and exploration when dealing with complex problems. In this paper, based on the basic RIME, we propose a DRIME algorithm that combines a distributed data-driven guided learning strategy, a soft-rime search strategy based on weighted mean, and a hard-rime puncture mechanism based on a candidate pool. The distributed data-driven guided learning strategy can determine whether the current population needs to be exploited or explored, and search accordingly. Data-driven approaches have already been widely adopted in other domains of artificial intelligence [82]. The soft-rime search strategy based on weighted mean strengthens the global search ability of the RIME algorithm and improves the probability of the RIME algorithm escaping the local optimum. The hard-rime puncture mechanism, based on candidate pooling, can co-ordinate the trend of exploitation and exploration. In conclusion, this paper makes significant contributions in several key aspects:

(1) A RIME variant has been proposed called DRIME, which combines a distributed data-driven guided learning strategy, a soft-rime search strategy based on weighted mean, and a hard-rime puncture mechanism based on a candidate pool.
(2) The DRIME algorithm overcomes the shortcomings of the basic RIME algorithm, including its lack of information exchange between populations, the deficiency of imbalance between exploitation and exploration, and insufficient global search capability, and it improves the probability of the RIME algorithm to jump out of the local optimum.
(3) The DRIME algorithm outperformed other RIME variants and variants of other algorithms in the CEC2017 test set, the CEC2022 test set, and engineering constraint optimization problems.

The remainder of the paper is structured as follows: Section 2 presents the model of the basic RIME algorithm. Section 3 provides the framework of DRIME, including details of the improvement mechanism, execution flowchart, pseudo-code, and time complexity analysis. The performance of the DRIME algorithm is fully evaluated in Section 4, including parameter sensitivity analysis, strategy effectiveness analysis, and comparative experiments. In Section 5, the performance of the DRIME algorithm is verified on engineering constraint optimization problems. Section 6 summarizes the work and provides an outlook for the future.

## 2. Overview of the RIME Algorithm

The RIME algorithm is a physics-based metaheuristic algorithm that consists of three phases: the population initialization phase, the soft-rime search phase, and the hard-rime puncture phase. In this section, these three phases are described in detail.

### 2.1. Initialization Phase of Rime Ice

In the RIME algorithm, each ice is regarded as an agent. Thus, the whole population contains ice agents. The initial position of the $i^{th}$ ice agent $R_{i}^{0}$ is generated by Equation (1):

(1) $$R_{i}^{0}=\left[x_{i,1},x_{i,2},\cdots ,x_{i,D}\right]=lb+rand\left(1,D\right)\times \left(ub-lb\right)$$

where D denotes the number of elements contained in each ice agent, i.e., the dimension of the problem; lb and ub restrict the range of each element, i.e., the upper and lower bounds of the optimization problem; $rand\left(1,D\right)$ is a vector whose elements range from 0 to 1.

### 2.2. Soft-Rime Search Phase

The soft-rime search phase is the main update method of the RIME algorithm. In this approach, the RIME algorithm mainly simulates the behavior of soft-rime fog growing in a wide area, as shown in Equation (2):

(2) $$R_{i}^{new}=R_{best}+rand\times \cos\left(\frac{FEs\times \pi }{10\times MaxFEs}\right)\times \left(1-round\left(\frac{5\times FEs}{MaxFEs}\right)/5\right)\times \left(rand\times \left(ub-lb\right)+lb\right),rand<E$$

(3) $$E=\sqrt{\frac{FEs}{MaxFEs}}$$

where $R_{i}^{new}$ is the updated position of the $i^{th}$ ice agent; $R_{best}$ is the current position of the best ice agent; FEs and MaxFEs denote the current number of evaluations and the maximum number of evaluations, respectively; $round\left(\cdot \right)$ denotes downward rounding of the result; E is a parameter that varies with the number of evaluations and is used to control whether or not the phase is executed.

### 2.3. Hard-Rime Puncture Phase

The hard-rime puncture phase is used to accelerate the convergence of the RIME population and is executed after each soft-rime search phase. This phase mainly simulates the growth state of hard-rime fog, as shown in Equation (4):

(4) $$R_{i}^{new}=R_{best},rand<F_{norm}$$

where $F_{norm}$ denotes the normalized value of the current agent fitness value.

Algorithm 1 displays the basic RIME algorithm’s pseudo code.
**Algorithm 1.** Pseudocode of basic RIME algorithm
Initialize the rime population *R* using Equation (1)
Obtain the current best agent and best fitness
**While**
FEs<MaxFEs
  Update the parameter E using Equation (3)
  **For**
*i =* 1: *N*
    **%%** Soft-rime search phase 
    **If**
rand<E
  Update ice agent using Equation (2)
    **End If**
    **%%** Hard-rime puncture phase 
    **If**
$rand<F_{norm}$
       Update ice agent using Equation (4)
    **End If**
  **End for**
  FEs=FEs+N
**End While**

## 3. Proposed DRIME Algorithm

Two update phases are used by the basic RIME algorithm to accomplish the problem space search. In order to make up for the limitations of the fundamental RIME, this research suggests three enhancement strategies based on these two phases. First, this paper proposes a distributed data-driven guided learning strategy, which can store the historical information of the population, then determine whether the current population is more in need of exploitation or exploration; it also enhances the information exchange between individuals in the population. The soft-rime search strategy, based on weighted mean, is proposed, which alternates with the original soft-rime search strategy to solve the problem of insufficient global search capability in the RIME algorithm. In addition, a hard-rime puncture mechanism, based on a candidate pool, is proposed, which balances the exploitation and exploration ability of the RIME algorithm.

### 3.1. Distributed Data-Driven Guided Learning Strategy (DGLS)

**Figure 2:**
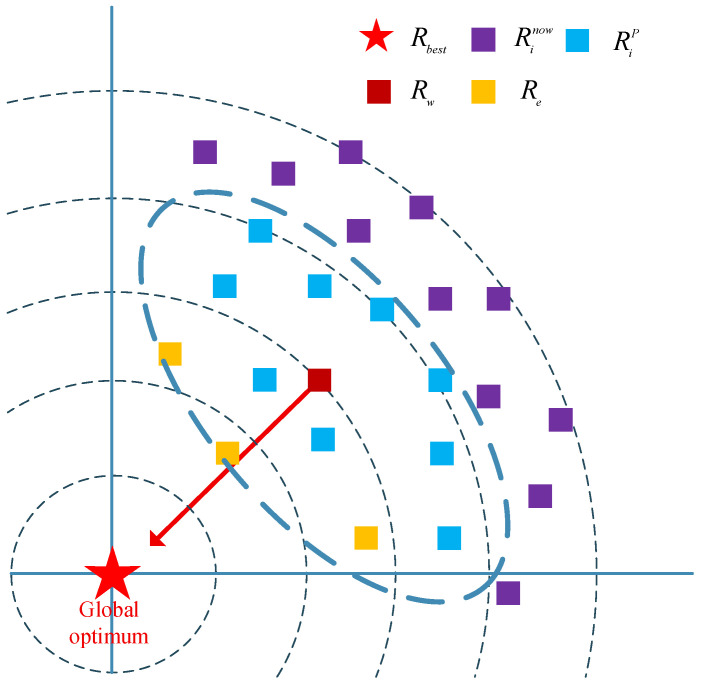
Schematic of DGLS.

The RIME algorithm updates the population mainly through a soft search phase, then accelerates the convergence through a hard search phase; that is, the basic RIME algorithm cannot determine whether the current state requires exploitation or exploration. For metaheuristic algorithms, they should perform more exploratory behaviors when global search is required, and perform more exploitative behaviors when local search is required. For this reason, metaheuristic algorithms need to determine what the current need is. Jia et al. propose a guided learning strategy that identifies the current needs of the algorithm and better balances exploration and utilization [83]. However, this strategy ignores the information exchange between populations and only utilizes the optimal individuals to adjust the population. Therefore, this paper proposes a distributed data-driven guided learning strategy (DGLS). The DGLS adopts the same judgment mechanism as GLS, but its updating method is different from GLS, as shown below:

(5) $$R_{i}^{new}=\left\{\begin{array}{l} \frac{\left(R_{i}^{now}+R_{w}+R_{e}\right)}{3}+g_{i},if\;V_{0}>\alpha \\ R_{w}+g_{i},if\;V_{0}\leq \alpha \end{array}\right.,g_{i}\sim N\left(0,Cov\right)$$

(6) $$Cov=\frac{1}{\left|P_{d}\right|}\sum_{i=1}^{\left|P_{d}\right|}\left(R_{i}^{P}-R_{w}\right)\times {\left(R_{i}^{P}-X_{w}\right)}^{T},R_{i}^{P}\in P_{d}$$

(7) $$R_{w}=\sum_{i=1}^{\left|P_{d}\right|}\omega_{i}\times R_{i}^{P},R_{i}^{P}\in P_{d}$$

(8) $$\omega_{i}=\ln\left(\left|P_{d}\right|+1\right)/\left(\sum_{i=1}^{\left|P_{d}\right|}\left(\ln\left(\left|P_{d}\right|+1\right)-\ln\left(i\right)\right)\right)$$

(9) $$V_{0}=std\left(St\right)\times B$$

(10) $$B=200/\left(lb-ub\right)$$

where $R_{w}$ is the weighted average position of the dominant population; $R_{e}$ is a randomly selected ice agent from the elite individuals. Inspired by the Grey Wolf Optimizer, we consider the top three ranked ice agents in terms of fitness values as elite individuals. Cov is the covariance matrix of the dominant population; $R_{i}^{P}$ is the $i^{th}$ ice agent in the dominant population; $\left|P_{d}\right|$ is the number of dominant groups. As shown in Figure 2, the dominant group pushes the population into promising territory, the elite individuals accelerate the convergence, and the agents themselves are used to correct the direction of the movement. $std\left(\cdot \right)$ is the function that calculates the standard deviation; St is the learning experience, storing the most recent Cmax ice agents in history—the number of storages will be discussed in the experimental section; B is used to normalize the $V_{0}$ in order to prevent it from being affected by the change of the upper and lower bounds; α is a parameter that determines whether exploitation or exploration is currently required—its value will be discussed in the experimental section.

During the execution of the DGLS, when $V_{0}>\alpha$, the RIME algorithm is considered to need exploitation at this point, and Equation (5) will be used to direct the population more towards exploitation. When $V_{0}\leq \alpha$, at this point, it is considered that the RIME algorithm needs to perform exploration, and Equation (5) will be used to direct the population to perform more global searches. Note that the strategy determines whether each dimension requires exploitation or exploration, which ensures that the exploration dimension still favors exploration and the exploitation dimension still favors exploitation. There is no sudden forced switch in the algorithm state, which better prevents GLS from dragging down the original performance of the algorithm in an attempt to improve it. This is therefore able to adapt to more complex high-latitude problems.

### 3.2. Soft-Rime Search Strategy Based on Weighted Mean (SWM)

The soft-rime search strategy of the RIME algorithm is to change the position of the population around the optimal ice agent. This approach can speed up its convergence. However, this approach will gradually cluster all the ice agents as the optimization proceeds, which will cause RIME to fall into the local optimum and the population diversity is greatly weakened. In addition, the soft-rime search strategy ignores the information exchange between populations, which is not conducive to expanding the search range. In this paper, we propose a soft-rime search strategy based on weighted mean (SWM). This strategy introduces the weighted average position of the dominant populations in Section 3.1, which can expand the search range of the populations and thus enhance the global search ability of RIME, as shown in Equation (11):

(11) $$R_{i}^{new}=R_{w}+rand\times \cos\left(\frac{FEs\times \pi }{10\times MaxFEs}\right)\times \left(1-round\left(\frac{5\times FEs}{MaxFEs}\right)/5\right)\times \left(rand\times \left(ub-lb\right)+lb\right),rand<E$$

It is worth noting that the SWM proposed in this paper is added as one of the update strategies in the hard search phase, rather than replacing the original hard search strategy. This is due to the fact that RIME needs to expand the search scope in the early stage and narrow the search scope in the later stage. Therefore, we utilize SWM more to update the population in the early stage and to apply the original search strategy more in the later stage. This choice is realized by comparing the size of rand and FEs/MaxFEs. When FEs/MaxFEs<rand, SWM is executed and vice versa, and the original search strategy is executed. As the number of evaluations increases, the value of FEs/MaxFEs becomes larger and the probability of the original search strategy being executed increases, which ensures convergence efficiency at later stages.

### 3.3. Hard-Rime Puncture Mechanism Based on Candidate Pool (HCP)

The RIME algorithm ensures convergence efficiency through a hard search phase; however, it has some drawbacks. For example, it lacks the information exchange between populations, and it increases the probability of falling into the local optimum and damages the diversity of populations by concentrating on optimal individuals. In order to improve the insufficiency of the hard search phase, this paper proposes a hard-rime puncture mechanism based on the candidate pool (HCP). The core of HCP is to construct a candidate pool. When each agent performs the hard search phase, an individual from the candidate pool is randomly selected for learning. In order to ensure convergence efficiency and address the above deficiencies, the construction of the candidate pool is crucial. First, inspired by the Equilibrium Optimizer (EO) [84], we put the ice agents with the top three fitness values into the candidate pool. These three ice agents can ensure sufficient convergence speed. Second, we put the weighted average position of the dominant population into the candidate pool; this individual can improve the quality of the population. Finally, we put the inverse position of the worst individual into the candidate pool. According to the reverse learning principle, the reverse position of an individual may be better. Therefore, this can expand the search range and help RIME to jump out of the local optimum. It is worth noting that this paper uses a stochastic reverse learning strategy, which is more random than the basic reverse learning strategy. The candidate pool is constructed by Equation (12):

(12) $$Pool=\left[R_{best},R_{second},R_{third},R_{w},R_{o}\right]$$

(13) $$R_{o}=lb+ub-rand\left(1,D\right)\times R_{worst}$$

(14) $$R_{i}^{new}=Pool_{random},rand<F_{norm}$$

where $R_{second}$ and $R_{third}$ are the second and third best ice agents; $R_{o}$ is the reverse individual of the worst ice agent, obtained by Equation (13); $Pool_{random}$ is a randomly selected individual from the pool. In a hard-rime puncture mechanism based on candidate pool, all ice agents will randomly choose one of the five individuals in the candidate pool to lean on. There is a 60 percent probability of accelerating convergence and a 40 percent probability of expanding the search. This method ensures the fast convergence of the population and achieves the preservation of individual diversity, preventing the risk of falling into the local optimum due to too-fast convergence.

### 3.4. The Structure of DRIME and Time Complexity

**Figure 3:**
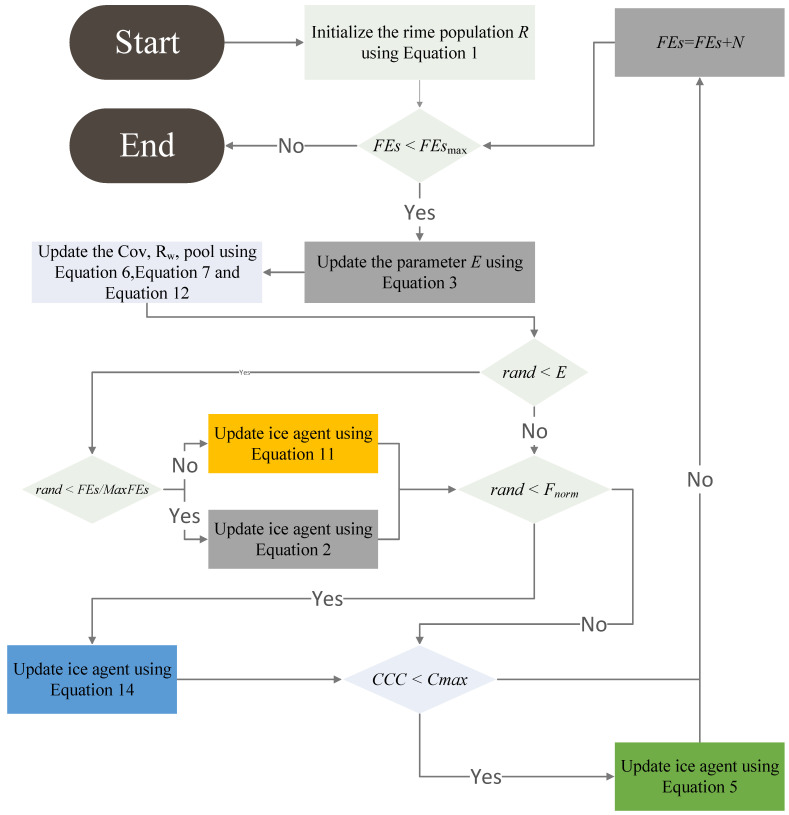
The flowchart of DRIME.

The DRIME algorithm achieves a notable increase in optimization capability by combining the above three strategies. Figure 3 provides the flowchart of the proposed DRIME, whose pseudo-code is demonstrated in Algorithm 2.
**Algorithm 2.** Pseudocode of DRIME algorithm
Initialize the rime population *R* using Equation (1)
Get the current best agent and best fitness 
**While**
FEs<MaxFEs
  Update the parameter E using Equation (3)
  Calculate the Cov using Equation (6)
  Calculate the $R_{w}$ using Equation (7)
  Construct the pool using Equation (12)
  **For**
*i =* 1: *N*
    **If**
rand<E
       **If** rand<FEs/MaxFEs
          Update ice agent using Equation (2) // Soft-rime search phase
       **Else**
          Update ice agent using Equation (11) // SWM
       **End If**
    **End If**
    **If**
$rand<F_{norm}$
       Update ice agent using Equation (14) // HCP
    **End If**
    *CCC* = *CCC* + 1 
    **If**
CCC<Cmax
       Update ice agent using Equation (5) // DGLS
       FEs=FEs+N
    **End If**
  **End for**
  FEs=FEs+N
**End While**

Algorithm time complexity indicates the trend of algorithm execution time as the amount of data increases, and it is an important metric for measurement. The population size of ice agents is represented by N; the dimension is D and the maximum iteration number is T. According to the calculation rules of time complexity, the time complexity of initializing the population is $O\left(N\times D\right)$. For the RIME algorithm, the time complexity of the soft-rime search phase is $O\left(N\times D\right)$. For DRIME, each ice agent will choose either the original soft-rime search phase or the soft-rime search strategy based on weighted mean, so the time complexity of this part is $O\left(N\times D\right)$. As for hard rime puncture mechanism, the time complexity of RIME and DRIME are the same $O\left(N\times D\right)$. The time complexity of the distributed data-driven guided learning strategy is $O\left(N\times D\right)$. Therefore, the total time complexity of RIME is as below:

$$O(RIME)=O\left(N\times D+T\times \left(Np\times D+Np\times D\right)\right)=O\left(\left(2T+1\right)\times N\times D\right)$$

We assume that DGLS is executed T1 times throughout the process, then the time complexity of DRIME is shown below:

$$O(DRIME)=O\left(N\times D+T\times \left(N\times D+N\times D\right)+T_{1}\times N\times D\right)=O\left(\left(2T+T_{1}+1\right)\times N\times D\right)$$

In summary, the time complexity of the proposed DRIME is higher than that of the RIME. The degree of DRIME complexity is related to the frequency of DGLS strategy enforcement. In the subsequent experiments, the stopping criterion uses the maximum number of function evaluations instead of the maximum number of iterations to ensure the fairness of the experiments.

## 4. Simulation Experiment and Result Analysis Using Test Suites

To evaluate the performance of the proposed DRIME, a detailed report on its performance on 29 CEC-2017 test functions and 12 CEC-2022 test functions is given in this section. Each algorithm is run independently 30 times on each test function, and the maximum number of function evaluations is uniformly set to 1000×D to weaken random fluctuations and to ensure statistical reliability. All the experiments are completed on the same hardware and software platforms listed in Table 1. The algorithms involved in the comparison follow the parameter configurations recommended in the original literature (see Table 2 for details).

### 4.1. Descriptions of Benchmark Functions and Performance Metrics

The CEC-2017 test suite and CEC-2022 test suite are the definitive benchmarks for evaluating the performance of metaheuristic algorithms and are designed to systematically measure the convergence speed, solution accuracy, local extremum escape, and global exploration of the algorithms. The two test suites provide an objective, comprehensive, and reproducible performance evaluation for DRIME. The CEC2017 test suite contains single-peaked functions (F1–F2), multi-peaked functions (F3–F9), hybrid functions (F10–F19), and composite functions (F20–F29). The CEC2022 test suite contains single-peaked functions (F1), basic functions (F2-F5), hybrid functions (F10–F19), and composite functions (F20–F29). The CEC2022 test suite contains single-peaked functions (F1), basic functions (F2–F5), and hybrid functions (F5), hybrid functions (F6–F8), and composite functions (F9–F12). These functions are generally non-linear, non-derivable, non-convex, and highly complex, and their details are summarized in Table 3 and Table 4.

The average value (Ave), standard deviation (Std), best value (Min), and ranking (Rank) obtained by DRIME, and the comparison algorithms when solving the benchmarking functions, will be used to evaluate their performance. Among them, Ave is used to measure the overall search performance of the algorithm, Std reflects the stability of the algorithm, and Min demonstrates the potential of the algorithm. In order to provide a comprehensive analysis of these data, three statistical tests were used in this experiment. The Wilcoxon rank sum test is a pairwise test that examines the strengths and weaknesses of DRIME and the comparison algorithms on individual functions. The Friedman test is a holistic test that determines whether or not there is a significant difference between DRIME and the comparison algorithms. The Nemenyi post hoc tests can further quantify the magnitude of differences between DRIME and comparison algorithms based on the results of the Friedman test.

### 4.2. Parameter Sensitivity Analysis Using CEC-2017 Test Suite

The proposed DGLS strategy contains two parameters, Cmax and α. Cmax determines how often the DGLS is executed, and, the larger Cmax is, the less frequently the DGLS is executed. Cmax determines whether the DGLS is performed more for exploitation or exploration, and, the larger α is, the more it tends to be explored. Therefore, it is necessary to perform parameter sensitivity analysis to determine the optimal parameter settings. In Section 4.2, we take a grid search approach. For Cmax, the values are taken from 5*N* to 25*N* with an interval of 5*N*. For α, the values are taken from 10 to 90 with an interval of 20. The DRIME algorithm with different parameters is run on the CEC-2017 test set 30 times.

**Figure 4:**
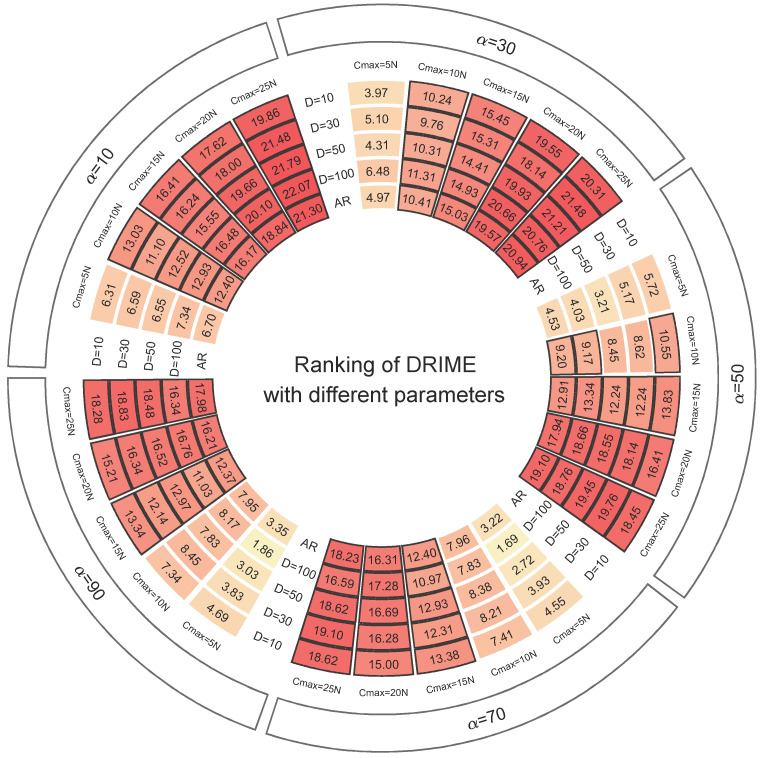
The Friedman ranking of DRIME with different parameters.

The results obtained by the DRIME algorithm with different parameters in the CEC-2017 test set will be analyzed by the Friedman rank sum test; the Friedman rankings of the algorithms will be shown, as in Figure 4. In Figure 4, AR denotes the average ranking of each algorithm under the four dimensions. Obviously, according to Figure 4, we find that, under the same α, the smaller Cmax is, the better the ranking of the corresponding algorithm is. This indicates that, the more frequently DGLS is executed, the greater its contribution to the performance of the DRIME algorithm. With the same Cmax, the performance of the algorithm gets progressively better when α increases from 10 to 70, and decreases when it further increases to 90. This indicates that, when DGLS prefers global search, it gives more boost to the DRIME algorithm. Therefore, we can conclude that DRIME performs best when Cmax=5N and α=70. Subsequent experiments will be conducted in this setting.

### 4.3. Strategy Effectiveness Analysis Using CEC-2017 Test Suite

In this subsection, we discuss the impact of the proposed three improvement mechanisms on the performance of DRIME. The DRIME-derived algorithms will be compared with the basic RIME under the CEC-2017 test suite. DRIME-D, DRIME-S, and DRIME-H are obtained by combining the basic RIME with the DGLS, SWM, and HCP, respectively. Similarly, the algorithm integrating DGLS and SWM is named DRIME-DS. DRIME-DH contains DGLS and HCP. The DRIME algorithm, excluding DGLS, is called DRIME-SH.

**Figure 5:**
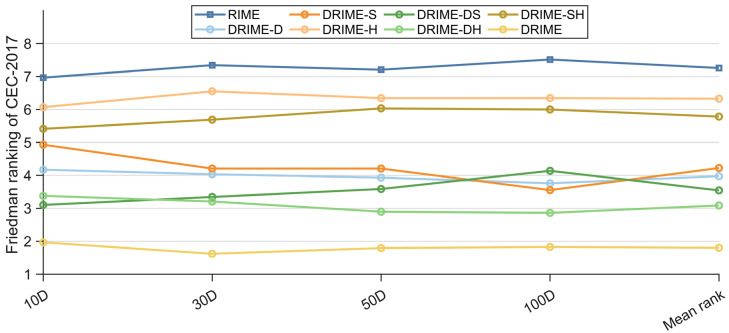
Friedman rankings of DRIME and DRIME variants with different strategies (a = 0.05).

The results obtained by the DRIME algorithm, with different strategies in the CEC-2017 test set, will be recorded in Table A1, Table A2, Table A3 and Table A4 in Appendix A. The three statistical tests mentioned in Section 4.2 will be used to analyze the differences between DRIME and the derived algorithms at a significance level of 0.05. Table 5 shows the results of the Friedman test for DRIME and the other variants on the CEC-2017 test set, and their rankings are visualized in Figure 5. According to Table 5, the *p*-values obtained for all four cases are less than 0.05, which indicates that there are significant differences between the algorithms involved in the experiment. The basic RIME algorithm is ranked last in all four dimensions, which indicates that all improvement strategies enhance the performance of the DRIME algorithm. By comparing the DRIME algorithms that combine a single improvement strategy, it can be seen that DGLS contributes the most to the DRIME algorithm, followed by SWM and HCP. DRIME-DH ranked first out of the three DRIME variants that combine two strategies, which suggests that the combination of DGLS and HCP improves DRIME more, although SWM contributes more than HCP to DRIME. DRIME-SH is ranked lower than DRIME-S on average, but better than DRIME-H. This suggests a lack of compatibility between SWM and HCP, and the fact that DRIME is ranked first in all cases suggests that the combination of the three strategies produces a more positive effect. Overall, to further determine whether each improvement strategy significantly improves the performance of DRIME, the Nemenyi post hoc test is used.

In the Nemenyi test, we will calculate the critical difference value (CDV) based on the number of algorithms and the number of functions, as shown in Equation (15). Here, M is the number of functions and K is the number of algorithms; $q_{a}$ is obtained from a statistical table obeying the F-distribution, and, in this paper, it is 3.0310.

(15) $$CDV=q_{a}\times \sqrt{\frac{K\left(K+1\right)}{6M}}$$

**Figure 6:**
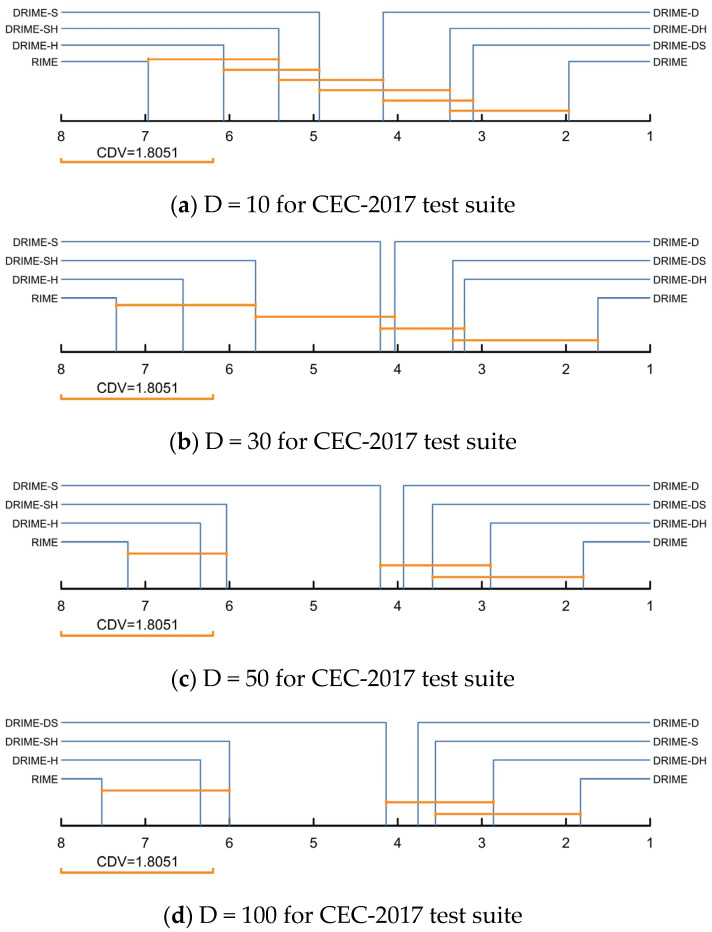
Nemenyi post hoc test of DRIME and DRIME variants with different strategies (a = 0.05).

After obtaining the CDV, we plot the results of the Nemenyi test in Figure 6, where there is no significant difference between the algorithms connected by line segments of length CDV. According to Figure 6, there is no significant difference between DRIME and DRIME-DS or DRIME-DH in 10/30/50 dimensions. This indicates that the enhancement of SWM and HCP for DRIME is limited, and the performance decay of DRIME is not significant regardless of the lack of SWM or HCP. In all four scenarios, there is no discernible difference between standard RIME and DRIME-H and DRIME-SH. Once more, this illustrates how HCP and SWM improve DRIME’s performance, but only to a certain extent.

Table 6 summarizes the results of the Wilcoxon rank sum test for DRIME and the other variants, where the symbols +/−/= indicate that the DRIME algorithm is significantly better/inferior/similar to the DRIME variants. According to Table 6, the DRIME algorithm and all its variants obtain more + than −, which means that the overall performance of these algorithms is superior to the basic RIME algorithm. The results of the comparison between the three DRIME variants combining a single improvement mechanism, and the basic RIME, again indicate that DGLS contributes the most to the DRIME algorithms, followed by SWM and HCP. The results of the three DRIME algorithms that combined two improvement methods show that, while these algorithms improved overall after integrating the two strategies, their performance on particular functions actually decreased. In summary, the three enhancement techniques put forth in this research are successful and contribute to raising the RIME algorithm’s performance.

### 4.4. Comparison Results with Other RIME Variants Using CEC-2017 Test Suite

In this subsection, we compare the proposed DRIME algorithm with other RIME variants on the CEC-2017 test set to illustrate the superiority of the DRIME algorithm. The selected RIME variants include ACGRIME [85], TERIME [86], IRIME [87], DNMRIME [88], GLSRIME [83], and HERIME [71]. The data obtained from the DRIME algorithm and the other RIME variants on the CEC-2017 test set are organized in Table A5, Table A6, Table A7 and Table A8 in Appendix A. In this part, we will show the results of Wilcoxon rank sum test, Friedman test, and Nemenyi test, and convergence analysis based on these data is performed.

**Figure 7:**
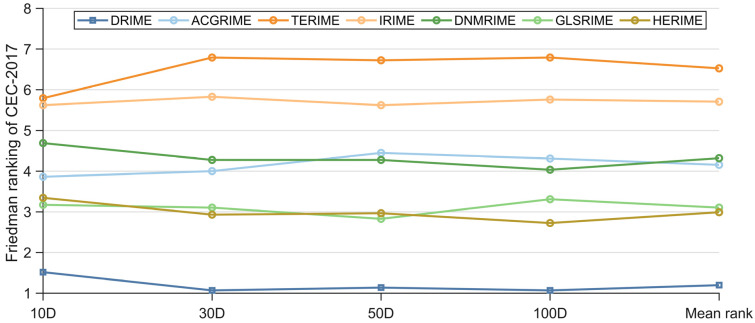
Friedman rankings of DRIME and other RIME variants (a = 0.05).

**Figure 8:**
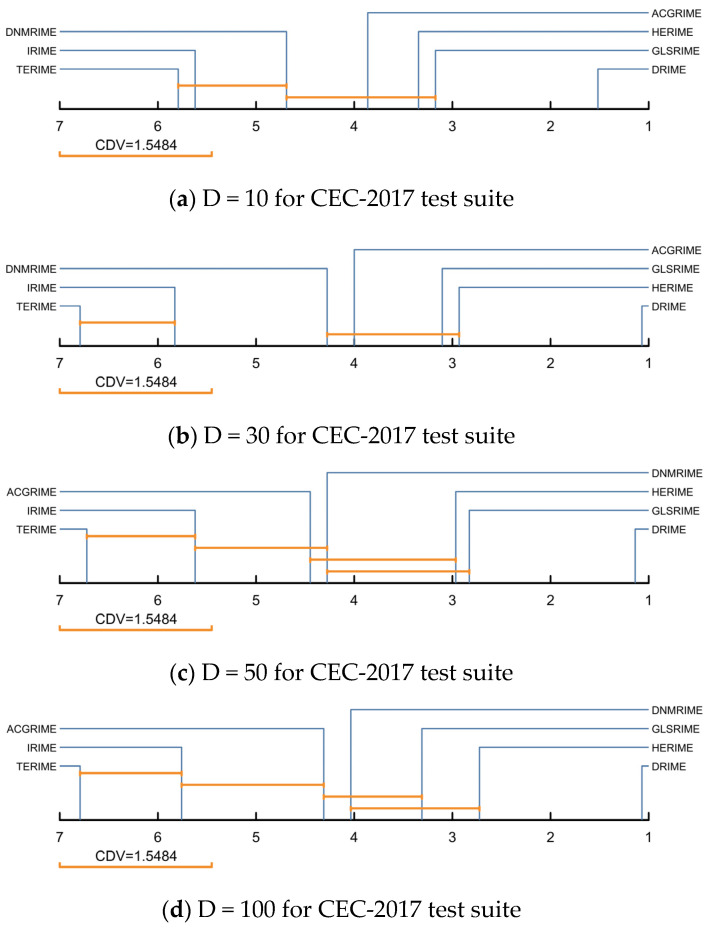
Nemenyi post hoc test of DRIME and other RIME variants.

The Friedman test results for the DRIME variants and other RIME variants on the CEC-2017 test set are listed in Table 7, where the rankings are shown in Figure 7. In Figure 7, DRIME performs the best in solving functions of different dimensions. HERIME and GLSRIME win against each other and are ranked in second and third places. It is worth noting that GLSRIME is obtained by combining the basic RIME with GLS policy. In this paper, we propose a distributed data-driven GLS strategy. The comparison results of DRIME and GLSRIME also indicate that the DGLS strategy proposed in this paper is superior to the GLS strategy. In addition, the rankings of the seven algorithms involved in the comparison fluctuated little. This indicates that the scalability of all these RIME variants is satisfactory. Similarly, Figure 8 quantifies the differences between the DRIME algorithm and other RIME variants using the Nemenyi post hoc test. According to the Nemenyi test, there are significant differences between DRIME and all RIME variants.

**Figure 9:**
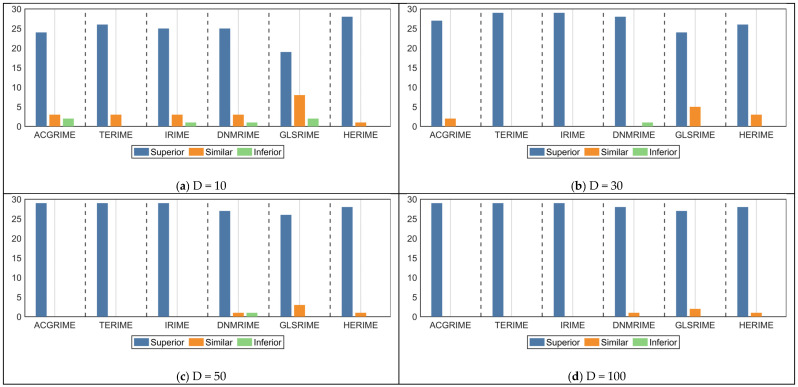
The number of +/=/− obtained by DRIME when compared to the RIEM variants.

The results of the Wilcoxon rank sum test for the DRIME algorithm and the RIME variant are shown in Table 8, where the symbol + indicates that DRIME is better, the symbol—indicates that DRIME is worse, and the symbol = indicates that DRIME and the comparator perform similarly. Figure 9 visualizes the number of +, −, and = obtained by DRIME when compared with the RIME variants. Taken as a whole, the DRIME algorithm is significantly better than other RIME variants for the vast majority of functions. Specifically, the DRIME algorithm is significantly superior (inferior) to ACGRIME on 109(2) functions, to TERIME on 113(0) functions, to IRIME on 112(1) functions, to DNMRIME on 108(3) functions, to GLSRIME on 96(2) functions, and to HERIME on 110(0) functions. Thus, the results of the Wilcoxon rank sum test demonstrate the distinct superiority of the DRIME algorithm.

**Figure 10:**
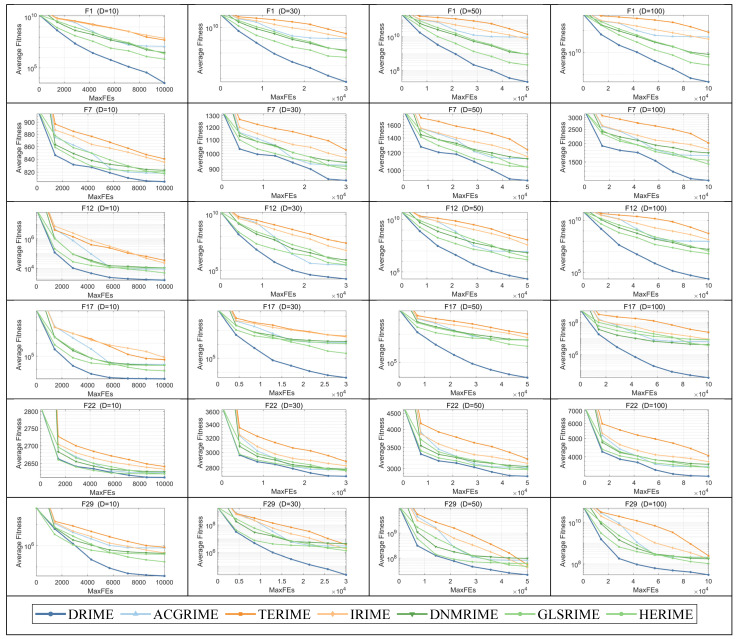
Convergence curves of DRIME and other RIEM variants.

**Figure A1:**
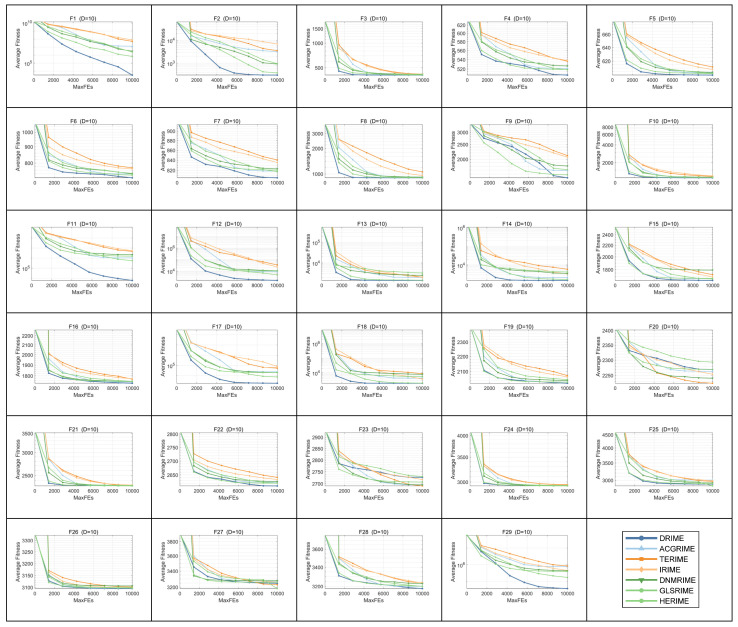
Convergence curves of DRIME and other RIEM variants when solving CEC-2017 test suite (D = 10).

**Figure A2:**
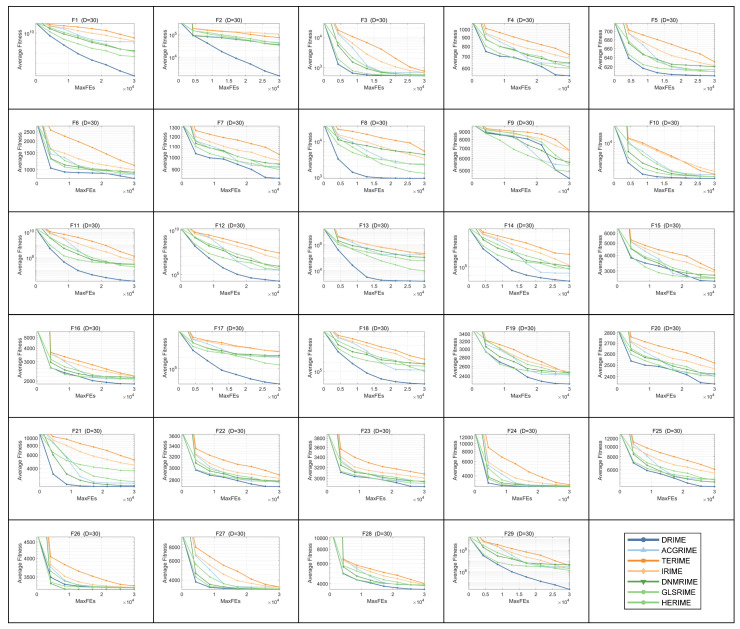
Convergence curves of DRIME and other RIEM variants when solving CEC-2017 test suite (D = 30).

**Figure A3:**
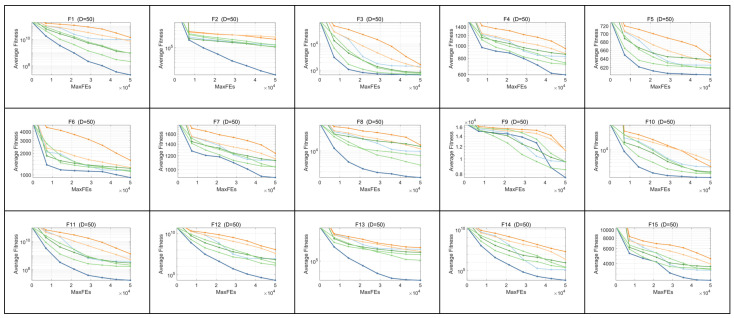

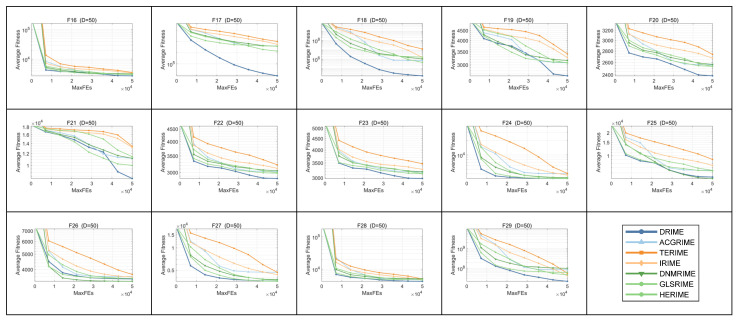
Convergence curves of DRIME and other RIEM variants when solving CEC-2017 test suite (D = 50).

**Figure A4:**
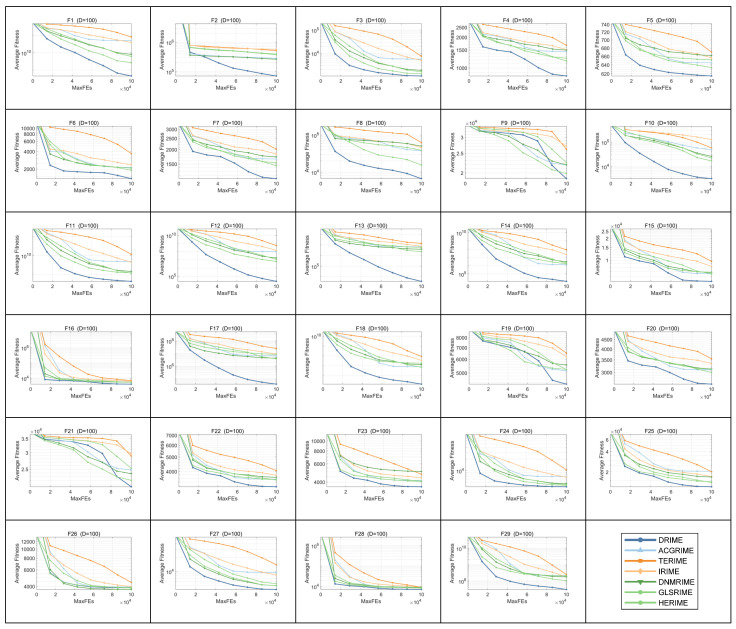
Convergence curves of DRIME and other RIEM variants when solving CEC-2017 test suite (D = 100).

To observe the trend of convergence in the DRIME algorithm throughout the search process, Figure 10 shows the convergence curves of the DRIME algorithm and other RIME variants in solving CEC-2017 test functions. In this part, the convergence curves of only six functions are selected to be presented, including the unimodal function F2, the multimodal function F4, the hybrid functions F2 and F4, and the combined functions F3 and F6. The complete convergence curves are organized in Figure A1, Figure A2, Figure A3 and Figure A4 in Appendix B. According to Figure 10, we can learn that the DRIME algorithm has excellent convergence efficiency. The DRIME algorithm’s ability to converge quickly and with high accuracy in the unimodal function F1 can be attributed to the developmental guidance of the DGLS strategy and the guidance of the excellent individuals in the HCP. The developmental guidance of the DGLS strategy partially allows for deeper searching of the dimensions that the individuals need to develop further. The inclusion of the three excellent individuals also allowed individuals to quickly move closer to the excellent individuals. Both mechanisms accelerate the convergence of the population and provide higher precision solutions. The DRIME algorithm can quickly get rid of stagnation and achieve better convergence when solving the multi-peak function F7, although it falls into stagnation in the early part of the search. This is due to reliance on the exploration guidance of the DGLS strategy, which further improves the quality of the population. The weighted individuals in the HCP and the inverse position of the worst individual enrich population diversity and expand the search range. The SWM reduces the probability of converging to the optimal individual, which also avoids falling into the local optimum to a certain extent. When solving F22 and F29, the DRIME algorithm can maintain a continuous downward trend and obtain better convergence values, although it converges a little slower in the early stages. Note that the convergence trend of the DRIME algorithm is consistent across functions of different dimensions, which suggests that it can be adapted to the optimization problems of different dimensions. In conclusion, the DRIME algorithm has efficient convergence and can provide high-quality solutions.

### 4.5. Comparison Results with Other Metaheuristic Variants Using CEC-2022 Test Suite

To further examine the search efficiency of the DRIME algorithm, this paper compares it with six improved algorithms on the CEC-2022 test set in this subsection. To further examine the search efficiency of the DRIME algorithm, this paper compares it with six improved algorithms on the CEC-2022 test set in this subsection. These six algorithms are EOSMA [89], GLS-MPA [83], ISGTOA [90], EMTLBO [91], LSHADE-SPACMA [92], and APSM-jSO [93]. The parameter settings for the comparison algorithms can be found in Table 2. The DRIME algorithm and the other improved algorithms will solve the CEC-2022 test function independently 30 times, and the experimental results will be recorded as shown in Table A9 and Table A10 in Appendix A.

Table 9 summarizes the results of the Friedman test and the Wilcoxon rank sum test regarding the performance of the DRIME algorithm and other improved algorithms on the CEC-2022 test set. First of all, we can conclude that there is a significant difference between the DRIME algorithm and the other improved algorithms, which is derived from the *p*-value. The DRIME algorithm ranked first on both the 10D and 20D functions, achieving average rankings of 2.167 and 1.917, respectively. In comparison with other algorithms, DRIME holds a significant superiority in at least eight functions. The DRIME algorithm is superior to EOSMA on 22 functions and inferior to it on only two functions. The DRIME algorithm is superior to GLS-MPA on 19 functions and inferior to it on only four functions. The DRIME algorithm is superior to ISGTOA on 17 functions and inferior to it on only six functions. The DRIME algorithm is superior to EMTLBO on 22 functions and inferior to it on only two functions. The DRIME algorithm is superior to LSHADE-SPACMA on 16 functions and inferior to it on only seven functions. The DRIME algorithm is superior to APSM-jSO on 21 functions and inferior to it on only two functions.

**Figure 11:**
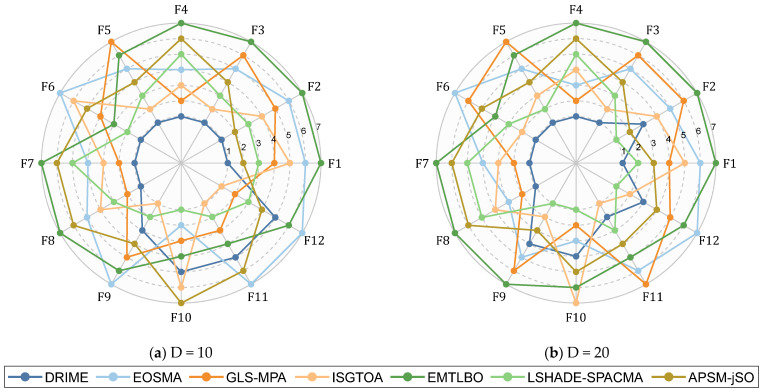
Algorithm performance sorting radar chart of DRIME and other RIEM variants.

**Figure 12:**
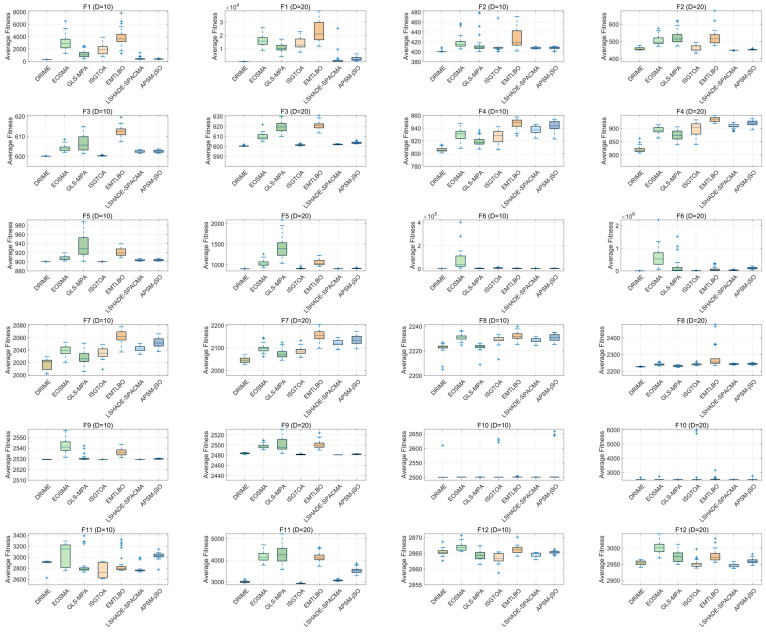
Boxplots of DRIME and other RIEM variants.

Figure 11 shows the ranking radar chart of the DRIME algorithm and other improved algorithms based on Ave. According to Figure 11, the area of the graph enclosed by the rankings of the DRIME algorithm on different functions is the smallest, which means that the DRIME algorithm has the best overall performance. The DRIME algorithm significantly underperforms when facing combinatorial functions as compared to other types of functions. The DRIME algorithm also underperforms when solving the low-dimensional functions of the CEC-2017 test set. This is due to the fact that low-dimensional problems only offer a limited number of populations, which hinders the effectiveness of the DGLS technique and makes it challenging to precisely describe the direction of population evolution. Figure 12 illustrates the distribution of solutions provided by the DRIME algorithm and other improved algorithms when solving the CEC-2022 test set. As shown in Figure 12, the DRIME algorithm has better distributions for most functions. These DRIME algorithms provide a more centralized distribution of solutions and compose lower boxes.

### 4.6. Discussion

In this subsection, we will discuss the advantages and disadvantages of the DRIME algorithm based on the results of four experiments. First, the parameter sensitivity analysis shows that the DGSL strategy prefers exploration guidance, and the execution frequency should be set higher. This is because the basic RIME algorithm seriously lacks the global search capability, so it leads to the fact that the parameters obtained at the end encourage DGLS to perform exploration bootstrapping more often. In addition, this paper illustrates the effectiveness and mutual reinforcement of different improvement strategies through ablation experiments. Both single improvement strategies and multiple improvement strategies can enhance the performance of the RIME algorithm. However, the SWM and HCP strategies do not have a significant enhancement effect when acting alone on the RIME algorithm. In order to compare the search capability of the DRIME algorithm horizontally, it is compared with multiple RIME variants on the CEC-2017 test set and with multiple other improved algorithms on the CEC-2022 test set. The results indicate that the performance of the DRIME algorithm is significantly better than these RIME variants and that it improved algorithms overall. However, DRIME does not perform as well on low-dimensional problems as it does on high-dimensional problems due to the fact that the small number of populations for low-dimensional problems does not correctly characterize the direction of population evolution, which diminishes the usefulness of the DGLS strategy.

## 5. Simulation Experiment and Result Analysis Using Engineering Constrained Optimization Problems

In this section, we apply the DRIME algorithm to 10 engineering constrained optimization problems to demonstrate its capability in tackling real-world optimization tasks. The detailed specifications of these 10 problems are listed in Table 10. Real-world optimization problems are almost always subject to constraints; hence, we transform the constrained problems into unconstrained ones. The penalty-function method—i.e., assigning a large penalty value to infeasible solutions so that they are progressively eliminated during the search—is employed for this purpose. The four highest-ranked RIME variants identified in Section 4.4, together with four top-performing variants of other algorithms in Section 4.5, are included in this experiment; the basic RIME algorithm is also included for comparison. Table 11 presents a comprehensive comparison between DRIME and the competing algorithms, including the outcomes of the Friedman test and the Wilcoxon rank sum test.

According to Table 11, DRIME achieves the best overall performance with an average ranking of 2.200, placing within the top three on every engineering problem. The +/=/− counts further underscore its superiority: the number of + symbols consistently exceeds that of—symbols. Although APSM-jSO and LSHADE-SPACMA outperform DRIME on two constrained engineering problems, DRIME significantly surpasses both algorithms on six others. In summary, DRIME demonstrates strong and reliable performance across all 10 engineering constrained optimization problems, highlighting its capability in tackling real-world optimization tasks.

## 6. Conclusions

This paper presents a distribution data-driven RIME algorithm named DRIME, which enriches its population diversity and improves the system’s global search capabilities. In DRIME, the distribution data-driven bootstrap learning strategy, based on the distribution data, collects information about the location of a population and determines whether the current demand is for exploitation or exploratory behaviors, which then guides that population to evolve correctly. A weighted mean position is introduced in the soft-rime search strategy to balance the ratio of exploitation and exploration. A candidate pool is added to the hard-rime puncture mechanism to enrich the population diversity while ensuring fast convergence. Parameter sensitivity analysis is used to identify the DRIME algorithm’s ideal parameter settings. The effectiveness of the improved strategy is confirmed by ablation experiments. Comparing the DRIME method to RIME variants and other enhanced algorithms on the CEC-2017 and CEC-2022 test sets, as well as engineering constraint optimization issues, validates its superiority. The DRIME algorithm is still deficient despite its strong performance. The DRIME algorithm needs to balance the relationship between the number of populations and the number of iterations. This is due to the fact that accurately describing the direction of population evolution requires a sufficiently large number of populations. In addition, we can explore a better switching mechanism instead of switching by the number of iterations. This is because, for each individual, different reference points are needed during the soft search phase. Therefore, we will make the following efforts in our future work: First, in order to mitigate the issue of inadequate population size, we shall attempt to provide external storage for historical individuals. Second, we will further investigate how to measure the needs of individuals so that they can be properly exploited or explored. Finally, because the majority of complicated optimization problems in the modern world are multi-objective and have multiple constraints, we will create a multi-objective version of the DRIME algorithm.

## Figures and Tables

**Table 1:** Platform specifications utilized for experiments.

Hardware	
CPU	AMD R9 7945HX (2.5 GHz)
RAM	32 GB
Operating system	64-bit Windows 11
Software	
Programming language	MATLAB R2023a

**Table 2:** Parameters setting of DRIME and other competing algorithms.

Common Setting	
Dimension of test suites	$\begin{array}{l} D=10/30/50/100\left(\mathrm{CEC}\text{-}2017\right)\\ D=10/20\left(\mathrm{CEC}\text{-}2022\right) \end{array}$
Algorithm setting	
DRIME	w=5,Cmax=5N,α=70
RIME	w=5
ACGRIME	a=4,w=5
TERIME	w=5
IRIME	$F_{1}=1,F_{2}=0.8,F_{3}=1,Cr_{1}=0.1,Cr_{2}=0.2,Cr_{3}=0.9,w=5$
DNMRIME	w=5,ms=0.1,jr=0.3,dr=0.05,jm=0.1
GLSRIME	w=5,Cmax=250,α=30
HERIME	w=5,λ=0.5
EOSMA	$a_{1}=2,a_{2}=1,GP=0.5,z=0.6,q=0.2$
GLS-MPA	FADs=0.2,P=0.5,Cmax=250,α=30
ISGTOA	λ=2
EMTLBO	s=0,p=0
LSHADE-SPACMA	p=,a=1.4,m=5,f=0.5
APSM-jSO	k=3,F=0.3,cr=0.8,h=6,a=1.3

**Table 3:** Detailed description of CEC2017 test functions.

Type	No.	Functions Name	Min
Unimodal functions	F1	Shifted and Rotated Bent Cigar Function	100
	F2	Shifted and Rotated Zakharov Function	300
Multimodal functions	F3	Shifted and Rotated Rosenbrock’s Function	400
	F4	Shifted and Rotated Rastrigin’s Function	500
	F5	Shifted and Rotated Expanded Scaffer’s F6 Function	600
	F6	Shifted and Rotated Lunacek Bi_Rastrigin Function	700
	F7	Shifted and Rotated Non-Continuous Rastrigin’s Function	800
	F8	Shifted and Rotated Levy Function	900
	F9	Shifted and Rotated Schwefel’s Function	1000
Hybrid functions	F10	Hybrid Function 1 (N = 3)	1100
	F11	Hybrid Function 2 (N = 3)	1200
	F12	Hybrid Function 3 (N = 3)	1300
	F13	Hybrid Function 4 (N = 4)	1400
	F14	Hybrid Function 5 (N = 4)	1500
	F15	Hybrid Function 6 (N = 4)	1600
	F16	Hybrid Function 6 (N = 5)	1700
	F17	Hybrid Function 6 (N = 5)	1800
	F18	Hybrid Function 6 (N = 5)	1900
	F19	Hybrid Function 6 (N = 6)	2000
Composition functions	F20	Composition Function 1 (N = 3)	2100
	F21	Composition Function 2 (N = 3)	2200
	F22	Composition Function 3 (N = 4)	2300
	F23	Composition Function 4 (N = 4)	2400
	F24	Composition Function 5 (N = 5)	2500
	F25	Composition Function 6 (N = 5)	2600
	F26	Composition Function 7 (N = 6)	2700
	F27	Composition Function 8 (N = 6)	2800
	F28	Composition Function 9 (N = 3)	2900
	F29	Composition Function 10 (N = 3)	3000

**Table 4:** Detailed description of CEC2022 test functions.

Type	No.	Functions Name	Min
Unimodal functions	F1	Shifted and Full Rotated Zakharov Function	300
Basic functions	F2	Shifted and Full Rotated Rosenbrock’s Function	400
	F3	Shifted and Full Rotated Expanded Schaffer’s f6 Function	600
	F4	Shifted and Full Rotated Non-Continuous Rastrigin’s Function	800
	F5	Shifted and Full Rotated Levy Function	900
Hybrid functions	F6	Hybrid Function 1 (N = 3)	1800
	F7	Hybrid Function 2 (N = 6)	2000
	F8	Hybrid Function 3 (N = 5)	2200
Composition functions	F9	Composition Function 1 (N = 5)	2300
	F10	Composition Function 2 (N = 4)	2400
	F11	Composition Function 3 (N = 5)	2600
	F12	Composition Function 4 (N = 6)	2700

**Table 5:** Friedman test results obtained by DRIME with different strategies (a = 0.05).

Test Suite	Dimension	RIME	DRIME-D	DRIME-S	DRIME-H	DRIME-DS	DRIME-DH	DRIME-SH	DRIME	*p*-Value
CEC-2017	10	6.966	4.172	4.931	6.069	3.103	3.379	5.414	1.966	2.62E-17
	30	7.345	4.034	4.207	6.552	3.345	3.207	5.690	1.621	2.46E-23
	50	7.207	3.931	4.207	6.345	3.586	2.897	6.034	1.793	3.07E-22
	100	7.517	3.759	3.552	6.345	4.138	2.862	6.000	1.828	3.47E-24
Mean rank		7.259	3.974	4.224	6.328	3.543	3.086	5.784	1.802	N/A
Overall rank		8	4	5	7	3	2	6	1	N/A

N/A—not applicable.

**Table 6:** Wilcoxon rank sum test results obtained by DRIME and DRIME variants with different strategies (a = 0.05).

vs. RIME (+/=/−)	CEC-2017 Test Suite			
	D = 10	D = 30	D = 50D	D = 100
DRIME-D	17/10/2	23/6/0	23/6/0	26/3/0
DRIME-S	8/20/1	24/5/0	22/7/0	27/2/0
DRIME-H	4/25/0	17/12/0	14/15/0	17/11/1
DRIME-DS	19/9/1	24/2/3	23/2/4	22/5/2
DRIME-DH	18/10/1	26/3/0	25/4/0	28/1/0
DRIME-SH	9/19/1	16/8/5	14/9/6	15/9/5
DRIME	19/8/2	25/3/1	25/2/2	27/1/1

**Table 7:** Friedman test results obtained by DRIME other RIME variants (a = 0.05).

Algorithm	CEC-2017 Test Suite					
	D = 10	D = 30	D = 50	D = 100	Mean Rank	Overall Rank
DRIME	1.517	1.069	1.138	1.069	1.198	1
ACGRIME	3.862	4.000	4.448	4.310	4.155	4
TERIME	5.793	6.793	6.724	6.793	6.526	9
IRIME	5.621	5.828	5.621	5.759	5.707	5
DNMRIME	4.690	4.276	4.276	4.034	4.319	7
GLSRIME	3.172	3.103	2.828	3.310	3.103	2
HERIME	3.345	2.931	2.966	2.724	2.991	3
*p*-value	3.98E-16	1.04E-26	1.14E-25	1.27E-26	N/A	N/A

N/A—not applicable.

**Table 8:** Wilcoxon rank sum test results obtained by DRIME and other RIME variants (a = 0.05).

DRIME vs. +/=/−	CEC-2017 Test Suite				
	10D	30D	50D	100D	Total
ACGRIME	24/3/2	27/2/0	29/0/0	29/0/0	109/5/2
TERIME	26/3/0	29/0/0	29/0/0	29/0/0	113/3/0
IRIME	25/3/1	29/0/0	29/0/0	29/0/0	112/3/1
DNMRIME	25/3/1	28/0/1	27/1/1	28/1/0	108/5/3
GLSRIME	19/8/2	24/5/0	26/3/0	27/2/0	96/18/2
HERIME	28/1/0	26/3/0	28/1/0	28/1/0	110/6/0

**Table 9:** Statistical test results obtained by DRIME and other improved algorithms (a = 0.05).

Algorithm	CEC-2022 Test Suite					
	D = 10		D = 20		Mean Rank	Overall Rank
	Rank	+/=/−	Rank	+/=/−		
DRIME	2.167	N/A	1.917	N/A	2.042	1
EOSMA	5.417	11/0/1	4.917	11/0/1	5.167	6
GLS-MPA	3.750	8/1/3	4.583	11/0/1	4.167	4
ISGTOA	3.167	9/0/3	3.250	8/1/3	3.208	3
EMTLBO	5.917	10/0/2	6.333	12/0/0	6.125	7
LSHADE-SPACMA	2.917	8/1/3	2.667	8/0/4	2.792	2
APSM-jSO	4.667	11/1/0	4.333	10/0/2	4.500	5
*p*-value	5.21E-05	N/A	5.39E-06	N/A	N/A	N/A

N/A—not applicable.

**Table 10:** Details of real-world constrained engineering optimization problems.

Problem	Name	D	g	h
1RC01	Tension/compression spring design problem	3	3	0
3RC03	Three-bar truss design problem	2	3	0
8RC06	Cantilever beam design problem	5	1	0
10RC08	Step-cone pulley problem	5	8	3
11RC09	Planetary gear train design	9	10	1
12RC10	Robot gripper problem	7	7	0
2RC02	Pressure vessel design problem	4	4	0
4RC04	Welded beam design problem	4	5	0
6RC05	Gear train design problem	4	1	1
9RC07	Multiple disk clutch brake design problem	5	7	0

**Table 11:** Comparison of DRIME and other competing algorithms in engineering constrained optimization problems.

No.	Index	DRIME	RIME	GLSRIME	HERIME	ACGRIME	IRIME	LSHADE-SPACMA	APSM-jSO	EOSMA	ISGTOA
RC01	Best	1.2740E-02	1.2771E-02	1.2771E-02	1.2760E-02	1.2736E-02	1.2994E-02	1.2668E-02	1.2668E-02	1.2893E-02	1.2980E-02
	Mean	1.3521E-02	1.7133E-02	1.7142E-02	1.4146E-02	1.4331E-02	1.4721E-02	1.2767E-02	1.2767E-02	1.3632E-02	1.5631E-02
	Std	9.4567E-04	2.5215E-03	2.3678E-03	1.5973E-03	1.5278E-03	2.1996E-03	1.0472E-04	1.0472E-04	6.2080E-04	2.8563E-03
	Rank	3	9	10	5	6	7	1	1	4	8
RC02	Best	2.6389E+02	2.6390E+02	2.6390E+02	2.6389E+02	2.6389E+02	2.6390E+02	2.6389E+02	2.6389E+02	2.6389E+02	2.6391E+02
	Mean	2.6389E+02	2.6426E+02	2.6426E+02	2.6426E+02	2.6425E+02	2.6434E+02	2.6396E+02	2.6396E+02	2.6394E+02	2.6461E+02
	Std	1.4260E-05	4.6889E-01	4.6889E-01	9.5125E-01	4.5378E-01	4.7651E-01	9.7391E-02	9.7391E-02	4.7128E-02	6.6927E-01
	Rank	1	6	6	8	5	9	3	3	2	10
RC03	Best	1.3400E+00	1.3469E+00	1.3480E+00	1.3470E+00	1.3415E+00	1.3879E+00	1.3452E+00	1.3452E+00	1.3424E+00	1.5199E+00
	Mean	1.3416E+00	1.3764E+00	1.3893E+00	1.4647E+00	1.3666E+00	1.6497E+00	1.4192E+00	1.4192E+00	1.3464E+00	2.0056E+00
	Std	1.3214E-03	3.4472E-02	3.3474E-02	9.8274E-02	2.7026E-02	1.5146E-01	7.1154E-02	7.1154E-02	2.8608E-03	3.2327E-01
	Rank	1	4	5	8	3	9	6	6	2	10
RC04	Best	1.6117E+01	1.6404E+01	1.6400E+01	1.6544E+01	1.6329E+01	1.6528E+01	1.6169E+01	1.6169E+01	1.6560E+01	1.7112E+01
	Mean	1.6491E+01	1.7062E+01	1.7124E+01	1.7488E+01	1.8099E+01	2.1606E+01	1.6940E+01	1.6940E+01	1.7117E+01	2.1567E+01
	Std	2.8586E-01	3.7382E-01	4.7502E-01	5.9431E-01	6.8694E+00	9.2808E+00	4.1347E-01	4.1347E-01	5.3814E-01	4.4724E+00
	Rank	1	4	6	7	8	10	2	2	5	9
RC05	Best	2.3545E-01	2.3646E-01	2.3526E-01	2.4000E-01	2.3646E-01	2.3767E-01	2.4426E-01	2.4426E-01	2.3526E-01	2.5000E-01
	Mean	2.4011E-01	2.4658E-01	2.4536E-01	2.6548E-01	2.6923E-01	2.5826E-01	2.5786E-01	2.5786E-01	2.4953E-01	4.5522E-01
	Std	3.7260E-03	8.4660E-03	9.1866E-03	2.2825E-02	8.8221E-02	2.0693E-02	1.2772E-02	1.2772E-02	1.1543E-02	3.1791E-01
	Rank	1	3	2	8	9	7	5	5	4	10
RC06	Best	3.1759E-16	1.0594E-16	1.3354E-16	1.0385E-16	8.8979E-17	1.2920E-16	7.3320E-17	7.3320E-17	3.7858E+00	1.2100E-16
	Mean	3.8084E+00	3.7333E+00	4.0742E+00	2.9825E+00	3.8061E-01	1.2416E-01	1.6401E-16	1.6401E-16	5.0977E+00	5.3276E+00
	Std	1.0772E+00	2.2000E+00	1.9343E+00	2.7949E+00	1.4487E+00	6.8006E-01	1.6328E-16	1.6328E-16	6.6783E-01	3.0407E+00
	Rank	7	6	8	5	4	3	1	1	9	10
RC07	Best	6.0316E+03	6.0607E+03	6.0970E+03	6.1805E+03	6.0737E+03	6.6278E+03	5.8727E+03	5.8727E+03	6.1816E+03	6.8880E+03
	Mean	6.4443E+03	7.1760E+03	7.2177E+03	7.9064E+03	6.7900E+03	9.7875E+03	6.7791E+03	6.7791E+03	6.7849E+03	1.2288E+04
	Std	2.6059E+02	5.9588E+02	5.1348E+02	1.0857E+03	5.8148E+02	2.7116E+03	7.8089E+02	7.8089E+02	3.7280E+02	3.6257E+03
	Rank	1	6	7	8	5	9	2	2	4	10
RC08	Best	1.6984E+00	1.7206E+00	1.7360E+00	1.7496E+00	1.7008E+00	1.7614E+00	1.7011E+00	1.7011E+00	1.7254E+00	1.8214E+00
	Mean	1.7199E+00	2.1473E+00	2.1167E+00	1.9213E+00	1.9516E+00	2.0730E+00	1.8130E+00	1.8130E+00	1.8242E+00	2.0852E+00
	Std	4.2266E-02	3.9882E-01	3.9066E-01	2.3583E-01	2.4368E-01	1.8136E-01	9.7650E-02	9.7650E-02	1.0342E-01	2.1107E-01
	Rank	1	10	9	5	6	7	2	2	4	8
RC09	Best	2.7009E-12	2.7009E-12	2.7009E-12	2.3078E-11	2.3078E-11	2.3078E-11	2.3078E-11	2.3078E-11	2.7009E-12	2.3078E-11
	Mean	9.9098E-10	2.8632E-09	3.5502E-09	4.8868E-09	1.0367E-08	1.3215E-08	2.7130E-09	2.7130E-09	1.2784E-09	9.7270E-09
	Std	1.4477E-09	2.9893E-09	6.9102E-09	9.5729E-09	1.3411E-08	3.1990E-08	3.2437E-09	3.2437E-09	2.4179E-09	1.1082E-08
	Rank	1	5	6	7	9	10	3	3	2	8
RC10	Best	3.9247E+08	3.9247E+08	3.9247E+08	3.9247E+08	3.9247E+08	3.9247E+08	3.9247E+08	3.9247E+08	3.9247E+08	3.9247E+08
	Mean	3.9247E+08	3.9247E+08	3.9247E+08	3.9247E+08	3.9247E+08	3.9247E+08	3.9247E+08	3.9247E+08	3.9247E+08	3.9247E+08
	Std	1.8187E-07	1.8187E-07	1.8187E-07	1.8187E-07	1.8187E-07	1.8187E-07	1.8187E-07	1.8187E-07	3.7527E-01	1.8187E-07
	Rank	1	1	1	1	1	1	1	1	10	1
Friedman Rank		2.200	5.850	6.450	6.600	6.000	7.600	3.450	3.450	4.600	8.800
+/=/−		N/A	8/1/1	9/1/0	8/2/0	8/1/1	8/1/1	6/2/2	6/2/2	8/2/0	9/1/0

N/A—not applicable.

## Data Availability

The data are provided within the manuscript.

## Appendix A

**Table A1:** Comparison of DRIME with different strategies in CEC-2017 test suite (D = 10).

No.	Index	RIME	DRIME-D	DRIME-S	DRIME-H	DRIME-DS	DRIME-DH	DRIME-SH	DRIME
F1	Best	1.2219E+05	3.5895E+03	4.5814E+04	2.2281E+04	3.5013E+03	3.7723E+03	3.1607E+04	5.1814E+02
	Avg	6.9551E+05	3.8625E+04	4.1107E+05	3.2524E+05	2.6761E+04	5.7547E+04	3.0731E+05	3.2371E+03
	Std	4.6496E+05	3.0267E+04	3.5935E+05	2.9557E+05	2.3337E+04	4.7798E+04	3.6184E+05	2.5383E+03
	Rank	8	3	7	6	2	4	5	1
F2	Best	3.1278E+02	3.0054E+02	3.0299E+02	3.0558E+02	3.0053E+02	3.0176E+02	3.1322E+02	3.0006E+02
	Avg	3.9436E+02	3.0469E+02	3.9843E+02	3.7192E+02	3.0425E+02	3.0541E+02	4.6467E+02	3.0034E+02
	Std	1.1728E+02	3.6627E+00	8.4635E+01	7.3071E+01	3.4653E+00	2.6526E+00	1.3086E+02	2.6805E-01
	Rank	6	3	7	5	2	4	8	1
F3	Best	4.0026E+02	4.0433E+02	4.0430E+02	4.0025E+02	4.0434E+02	4.0136E+02	4.0551E+02	4.0448E+02
	Avg	4.1270E+02	4.0643E+02	4.0667E+02	4.0681E+02	4.0681E+02	4.0623E+02	4.0713E+02	4.0637E+02
	Std	1.8963E+01	9.6669E-01	1.2497E+00	1.8525E+00	6.8944E-01	1.3593E+00	7.6417E-01	6.6339E-01
	Rank	8	3	4	6	5	1	7	2
F4	Best	5.0536E+02	5.0504E+02	5.0311E+02	5.0324E+02	5.0334E+02	5.0501E+02	5.0384E+02	5.0398E+02
	Avg	5.1764E+02	5.1232E+02	5.1263E+02	5.1300E+02	5.0775E+02	5.1346E+02	5.0934E+02	5.0746E+02
	Std	8.1125E+00	4.7715E+00	5.5847E+00	5.3996E+00	3.0514E+00	4.7521E+00	3.9123E+00	3.4503E+00
	Rank	8	4	5	6	2	7	3	1
F5	Best	6.0045E+02	6.0010E+02	6.0014E+02	6.0013E+02	6.0007E+02	6.0010E+02	6.0020E+02	6.0002E+02
	Avg	6.0104E+02	6.0031E+02	6.0053E+02	6.0087E+02	6.0021E+02	6.0023E+02	6.0077E+02	6.0008E+02
	Std	5.6063E-01	2.2793E-01	3.4640E-01	6.0573E-01	1.1904E-01	9.4700E-02	5.0125E-01	5.9760E-02
	Rank	8	4	5	7	2	3	6	1
F6	Best	7.1053E+02	7.0746E+02	7.1804E+02	7.1631E+02	7.1565E+02	7.1397E+02	7.1299E+02	7.1247E+02
	Avg	7.2943E+02	7.2428E+02	7.2762E+02	7.2509E+02	7.2294E+02	7.2509E+02	7.2090E+02	7.1894E+02
	Std	7.9386E+00	7.2337E+00	5.6998E+00	5.2170E+00	5.5757E+00	5.3730E+00	4.8690E+00	5.8972E+00
	Rank	8	4	7	5	3	6	2	1
F7	Best	8.0846E+02	8.0507E+02	8.0443E+02	8.0405E+02	8.0141E+02	8.0404E+02	8.0220E+02	8.0100E+02
	Avg	8.1839E+02	8.1435E+02	8.1079E+02	8.1017E+02	8.0724E+02	8.1169E+02	8.0567E+02	8.0552E+02
	Std	6.7686E+00	5.2871E+00	4.0586E+00	4.2998E+00	3.7404E+00	5.0377E+00	2.1891E+00	4.1056E+00
	Rank	8	7	5	4	3	6	2	1
F8	Best	9.0019E+02	9.0000E+02	9.0009E+02	9.0004E+02	9.0001E+02	9.0003E+02	9.0004E+02	9.0000E+02
	Avg	9.0174E+02	9.0030E+02	9.0056E+02	9.0180E+02	9.0010E+02	9.0015E+02	9.0033E+02	9.0001E+02
	Std	2.5919E+00	4.1355E-01	3.2045E-01	3.3080E+00	1.3445E-01	1.2222E-01	3.0524E-01	2.8392E-02
	Rank	7	4	6	8	2	3	5	1
F9	Best	1.1590E+03	1.1331E+03	1.1264E+03	1.0151E+03	1.1404E+03	1.1235E+03	1.0113E+03	1.1443E+03
	Avg	1.6026E+03	1.5593E+03	1.5354E+03	1.5796E+03	1.5588E+03	1.5580E+03	1.5884E+03	1.5124E+03
	Std	2.1943E+02	2.0724E+02	2.4918E+02	2.7331E+02	2.5311E+02	2.2399E+02	2.3342E+02	2.4264E+02
	Rank	8	5	2	6	4	3	7	1
F10	Best	1.1056E+03	1.1024E+03	1.1025E+03	1.1047E+03	1.1037E+03	1.1030E+03	1.1091E+03	1.1030E+03
	Avg	1.1210E+03	1.1102E+03	1.1150E+03	1.1171E+03	1.1101E+03	1.1105E+03	1.1178E+03	1.1076E+03
	Std	1.2616E+01	5.4217E+00	7.0316E+00	7.2381E+00	4.5713E+00	5.5135E+00	7.1831E+00	2.6459E+00
	Rank	8	3	5	6	2	4	7	1
F11	Best	4.9078E+03	2.6766E+03	9.0094E+03	7.5456E+03	5.4051E+03	2.5449E+03	5.1492E+04	2.2731E+03
	Avg	1.4452E+06	1.7971E+04	8.3751E+05	1.1895E+06	1.9997E+04	1.6864E+04	8.0360E+05	6.0176E+03
	Std	1.5106E+06	1.6292E+04	1.4605E+06	1.3230E+06	2.1421E+04	1.1322E+04	6.2834E+05	2.4674E+03
	Rank	8	3	6	7	4	2	5	1
F12	Best	1.6575E+03	1.3300E+03	1.4944E+03	1.8916E+03	1.3600E+03	1.3167E+03	2.2680E+03	1.3216E+03
	Avg	1.0495E+04	2.9898E+03	1.1172E+04	9.4386E+03	1.8446E+03	1.8498E+03	7.9133E+03	1.5404E+03
	Std	8.5958E+03	3.1775E+03	8.6055E+03	4.2887E+03	7.9424E+02	1.5742E+03	3.4327E+03	6.2107E+02
	Rank	7	4	8	6	2	3	5	1
F13	Best	1.4227E+03	1.4107E+03	1.4267E+03	1.4429E+03	1.4074E+03	1.4112E+03	1.4312E+03	1.4060E+03
	Avg	4.1977E+03	1.4315E+03	4.1473E+03	2.6994E+03	1.4259E+03	1.4227E+03	2.8549E+03	1.4197E+03
	Std	3.4476E+03	3.0089E+01	3.2898E+03	1.6586E+03	6.1351E+00	6.3962E+00	1.4309E+03	7.9296E+00
	Rank	8	4	7	5	3	2	6	1
F14	Best	1.5158E+03	1.5034E+03	1.5234E+03	1.5440E+03	1.5056E+03	1.5036E+03	1.5238E+03	1.5024E+03
	Avg	5.0169E+03	1.7054E+03	4.1238E+03	3.4168E+03	1.5147E+03	1.5104E+03	2.9819E+03	1.5065E+03
	Std	5.2530E+03	1.0091E+03	4.1005E+03	2.4671E+03	5.4618E+00	6.5682E+00	1.7433E+03	3.7066E+00
	Rank	8	4	7	6	3	2	5	1
F15	Best	1.6020E+03	1.6014E+03	1.6027E+03	1.6021E+03	1.6038E+03	1.6018E+03	1.6036E+03	1.6034E+03
	Avg	1.6849E+03	1.6742E+03	1.6769E+03	1.6918E+03	1.6720E+03	1.6836E+03	1.7155E+03	1.6465E+03
	Std	9.2352E+01	8.6779E+01	8.2573E+01	9.9287E+01	8.1837E+01	9.7465E+01	1.0655E+02	5.5149E+01
	Rank	6	3	4	7	2	5	8	1
F16	Best	1.7176E+03	1.7051E+03	1.7016E+03	1.7218E+03	1.7134E+03	1.7072E+03	1.7223E+03	1.7061E+03
	Avg	1.7558E+03	1.7502E+03	1.7320E+03	1.7664E+03	1.7411E+03	1.7362E+03	1.7407E+03	1.7348E+03
	Std	3.9600E+01	4.3662E+01	1.5943E+01	4.5421E+01	1.8745E+01	2.0316E+01	8.2779E+00	1.1868E+01
	Rank	7	6	1	8	5	3	4	2
F17	Best	2.8204E+03	1.8496E+03	4.4786E+03	2.3324E+03	1.8331E+03	1.8191E+03	2.0401E+03	1.8130E+03
	Avg	2.0451E+04	3.8993E+03	1.9077E+04	1.4101E+04	1.9507E+03	3.1183E+03	6.9415E+03	1.8338E+03
	Std	1.4541E+04	4.5940E+03	1.0767E+04	9.6656E+03	1.6053E+02	2.2940E+03	4.2099E+03	1.3569E+01
	Rank	8	4	7	6	2	3	5	1
F18	Best	1.9076E+03	1.9038E+03	1.9096E+03	1.9216E+03	1.9029E+03	1.9020E+03	1.9891E+03	1.9019E+03
	Avg	6.1091E+03	2.0331E+03	5.1171E+03	4.4412E+03	1.9062E+03	1.9053E+03	4.2581E+03	1.9038E+03
	Std	6.0540E+03	4.8146E+02	4.8035E+03	2.9502E+03	1.6738E+00	1.7768E+00	1.6743E+03	1.1183E+00
	Rank	8	4	7	6	3	2	5	1
F19	Best	2.0105E+03	2.0013E+03	2.0152E+03	2.0091E+03	2.0111E+03	2.0047E+03	2.0215E+03	2.0022E+03
	Avg	2.0317E+03	2.0291E+03	2.0288E+03	2.0314E+03	2.0299E+03	2.0273E+03	2.0292E+03	2.0252E+03
	Std	1.0805E+01	1.4478E+01	1.0101E+01	9.5125E+00	1.1239E+01	1.1701E+01	6.0957E+00	6.9262E+00
	Rank	8	4	3	7	6	2	5	1
F20	Best	2.2004E+03	2.2001E+03	2.2011E+03	2.2002E+03	2.2001E+03	2.2002E+03	2.2022E+03	2.2001E+03
	Avg	2.2733E+03	2.2740E+03	2.2653E+03	2.2692E+03	2.2622E+03	2.2608E+03	2.2784E+03	2.2694E+03
	Std	5.9619E+01	5.6190E+01	5.5737E+01	5.5634E+01	5.3994E+01	5.6342E+01	4.8285E+01	5.1784E+01
	Rank	6	7	3	4	2	1	8	5
F21	Best	2.2012E+03	2.2156E+03	2.2007E+03	2.2269E+03	2.3018E+03	2.2451E+03	2.3022E+03	2.3002E+03
	Avg	2.2955E+03	2.2976E+03	2.2957E+03	2.3019E+03	2.3053E+03	2.3028E+03	2.3068E+03	2.3024E+03
	Std	3.0607E+01	2.2929E+01	2.9283E+01	2.0295E+01	1.8477E+00	1.1029E+01	2.3031E+00	1.4445E+00
	Rank	1	3	2	4	7	6	8	5
F22	Best	2.6095E+03	2.6084E+03	2.6073E+03	2.6079E+03	2.6012E+03	2.6066E+03	2.6004E+03	2.6030E+03
	Avg	2.6203E+03	2.6167E+03	2.6156E+03	2.6169E+03	2.6107E+03	2.6162E+03	2.6142E+03	2.6109E+03
	Std	7.0979E+00	5.5420E+00	6.4120E+00	6.5452E+00	5.1061E+00	5.3966E+00	6.7360E+00	5.5501E+00
	Rank	8	6	4	7	1	5	3	2
F23	Best	2.5019E+03	2.5005E+03	2.5015E+03	2.5014E+03	2.5006E+03	2.5008E+03	2.5031E+03	2.5014E+03
	Avg	2.7064E+03	2.6945E+03	2.7068E+03	2.7042E+03	2.7046E+03	2.6893E+03	2.6986E+03	2.7270E+03
	Std	8.8530E+01	9.4538E+01	8.5739E+01	8.8171E+01	8.1301E+01	1.0234E+02	8.6021E+01	4.2795E+01
	Rank	6	2	7	4	5	1	3	8
F24	Best	2.8979E+03	2.8980E+03	2.8990E+03	2.8991E+03	2.8980E+03	2.8978E+03	2.9000E+03	2.8982E+03
	Avg	2.9270E+03	2.9321E+03	2.9377E+03	2.9420E+03	2.9343E+03	2.9275E+03	2.9414E+03	2.9324E+03
	Std	2.3831E+01	2.2103E+01	1.9591E+01	1.4616E+01	1.9988E+01	2.3727E+01	1.2905E+01	2.0593E+01
	Rank	1	3	6	8	5	2	7	4
F25	Best	2.8235E+03	2.8080E+03	2.6127E+03	2.8115E+03	2.8026E+03	2.8071E+03	2.8136E+03	2.9001E+03
	Avg	2.9097E+03	2.9013E+03	2.8994E+03	2.9110E+03	2.8949E+03	2.8984E+03	2.9043E+03	2.9004E+03
	Std	3.1105E+01	2.1785E+01	6.1009E+01	3.8008E+01	2.4935E+01	2.7270E+01	2.2530E+01	8.9534E-01
	Rank	7	5	3	8	1	2	6	4
F26	Best	3.0905E+03	3.0895E+03	3.0901E+03	3.0886E+03	3.0905E+03	3.0876E+03	3.0902E+03	3.0900E+03
	Avg	3.0985E+03	3.0980E+03	3.0964E+03	3.0991E+03	3.0957E+03	3.0955E+03	3.0960E+03	3.0952E+03
	Std	9.4378E+00	1.4099E+01	2.5506E+00	1.0789E+01	2.2082E+00	3.3985E+00	2.4771E+00	3.0830E+00
	Rank	7	6	5	8	3	2	4	1
F27	Best	3.1015E+03	3.1004E+03	3.1023E+03	3.1024E+03	3.1008E+03	3.1010E+03	3.1016E+03	3.1003E+03
	Avg	3.2572E+03	3.2333E+03	3.2063E+03	3.2536E+03	3.2402E+03	3.2613E+03	3.2907E+03	3.2472E+03
	Std	1.1253E+02	1.2342E+02	1.0036E+02	1.1942E+02	1.3029E+02	1.2310E+02	1.3368E+02	1.4411E+02
	Rank	6	2	1	5	3	7	8	4
F28	Best	3.1525E+03	3.1427E+03	3.1457E+03	3.1473E+03	3.1556E+03	3.1360E+03	3.1557E+03	3.1510E+03
	Avg	3.1995E+03	3.1815E+03	3.1848E+03	3.1870E+03	3.1844E+03	3.1749E+03	3.1883E+03	3.1781E+03
	Std	4.9256E+01	2.5236E+01	2.6601E+01	2.9648E+01	1.4725E+01	1.7328E+01	1.5833E+01	1.5368E+01
	Rank	8	3	5	6	4	1	7	2
F29	Best	8.0433E+03	3.9963E+03	1.0205E+04	5.0418E+03	4.1305E+03	4.0197E+03	2.5672E+04	3.6850E+03
	Avg	3.2685E+05	3.2953E+05	1.7603E+05	2.5267E+05	1.1084E+05	3.0453E+05	1.5266E+05	3.7257E+04
	Std	5.4279E+05	4.9300E+05	3.4969E+05	3.9368E+05	3.9324E+05	5.4020E+05	3.0872E+05	1.7219E+05
	Rank	7	8	4	5	2	6	3	1

**Table A2:** Comparison of DRIME with different strategies in CEC-2017 test suite (D = 30).

No.	Index	RIME	DRIME-D	DRIME-S	DRIME-H	DRIME-DS	DRIME-DH	DRIME-SH	DRIME
F1	Best	1.3991E+07	7.6444E+05	4.0734E+06	5.3393E+06	9.5526E+05	1.6346E+06	4.1556E+06	1.0336E+05
	Avg	3.0638E+07	5.2785E+06	1.3978E+07	1.9984E+07	4.3864E+06	4.2573E+06	1.8225E+07	5.4087E+05
	Std	1.1145E+07	3.4771E+06	7.2505E+06	8.9374E+06	3.5656E+06	2.2210E+06	1.2113E+07	4.4719E+05
	Rank	8	4	5	7	3	2	6	1
F2	Best	1.1749E+04	1.2062E+03	1.3494E+04	9.7901E+03	1.1740E+03	1.7719E+03	2.1753E+04	5.5212E+02
	Avg	3.1796E+04	3.2963E+03	2.5759E+04	2.5010E+04	3.2855E+03	4.1048E+03	3.1233E+04	1.4789E+03
	Std	9.2105E+03	1.2282E+03	6.3144E+03	9.0928E+03	1.3508E+03	1.6924E+03	5.6478E+03	6.3419E+02
	Rank	8	3	6	5	2	4	7	1
F3	Best	4.8063E+02	4.8709E+02	4.7930E+02	4.8668E+02	5.1229E+02	4.7412E+02	5.1719E+02	4.9689E+02
	Avg	5.1810E+02	5.1983E+02	5.2057E+02	5.2457E+02	5.2447E+02	5.1989E+02	5.2889E+02	5.2169E+02
	Std	2.1827E+01	2.0361E+01	2.1018E+01	2.2904E+01	5.8185E+00	2.2392E+01	6.0839E+00	7.5835E+00
	Rank	1	2	4	7	6	3	8	5
F4	Best	5.5683E+02	5.4282E+02	5.2882E+02	5.4254E+02	5.2675E+02	5.3871E+02	5.3481E+02	5.3063E+02
	Avg	5.9960E+02	5.6360E+02	5.5669E+02	5.7714E+02	5.4463E+02	5.5995E+02	5.6565E+02	5.4376E+02
	Std	2.2829E+01	1.3738E+01	1.4349E+01	1.9243E+01	1.0900E+01	1.5000E+01	1.9216E+01	1.2058E+01
	Rank	8	5	3	7	2	4	6	1
F5	Best	6.0381E+02	6.0096E+02	6.0155E+02	6.0181E+02	6.0089E+02	6.0086E+02	6.0262E+02	6.0037E+02
	Avg	6.0871E+02	6.0202E+02	6.0259E+02	6.0645E+02	6.0298E+02	6.0203E+02	6.0725E+02	6.0146E+02
	Std	3.8322E+00	9.2152E-01	7.3774E-01	3.0662E+00	1.3695E+00	9.0190E-01	2.6206E+00	7.5368E-01
	Rank	8	2	4	6	5	3	7	1
F6	Best	8.1869E+02	7.9040E+02	7.9111E+02	8.1801E+02	7.7101E+02	7.8234E+02	7.9331E+02	7.6244E+02
	Avg	8.8809E+02	8.2999E+02	8.3024E+02	8.6215E+02	8.2357E+02	8.2540E+02	8.3519E+02	8.0749E+02
	Std	3.5951E+01	2.0906E+01	1.8906E+01	2.3889E+01	2.4613E+01	2.5534E+01	2.3051E+01	3.0163E+01
	Rank	8	4	5	7	2	3	6	1
F7	Best	8.5403E+02	8.3943E+02	8.3334E+02	8.3354E+02	8.2495E+02	8.4177E+02	8.2817E+02	8.1895E+02
	Avg	8.9334E+02	8.6051E+02	8.5511E+02	8.6763E+02	8.3809E+02	8.6398E+02	8.4979E+02	8.3161E+02
	Std	2.5469E+01	1.5079E+01	1.3987E+01	1.6239E+01	8.6010E+00	1.6606E+01	1.2685E+01	7.4953E+00
	Rank	8	5	4	7	2	6	3	1
F8	Best	9.9061E+02	9.0361E+02	9.1102E+02	9.1313E+02	9.0439E+02	9.0161E+02	9.4313E+02	9.0167E+02
	Avg	1.7869E+03	9.2965E+02	9.5319E+02	1.2616E+03	9.3744E+02	9.2366E+02	1.0759E+03	9.1526E+02
	Std	1.1099E+03	2.2604E+01	3.3780E+01	3.5714E+02	3.3849E+01	2.5653E+01	9.7015E+01	1.0496E+01
	Rank	8	3	5	7	4	2	6	1
F9	Best	3.9506E+03	3.6228E+03	3.8761E+03	3.4490E+03	4.0650E+03	3.3254E+03	3.5049E+03	3.0332E+03
	Avg	4.9603E+03	4.6841E+03	4.7693E+03	4.7902E+03	5.0863E+03	4.7270E+03	4.7269E+03	4.4306E+03
	Std	6.1288E+02	5.8455E+02	5.6356E+02	7.0290E+02	6.8405E+02	7.0641E+02	7.0798E+02	6.4303E+02
	Rank	7	2	5	6	8	4	3	1
F10	Best	1.2250E+03	1.1796E+03	1.2223E+03	1.2000E+03	1.1741E+03	1.1529E+03	1.2365E+03	1.1616E+03
	Avg	1.3521E+03	1.2236E+03	1.2802E+03	1.2946E+03	1.2258E+03	1.2210E+03	1.2868E+03	1.2087E+03
	Std	7.4446E+01	2.4881E+01	2.7870E+01	4.4833E+01	2.7521E+01	3.1192E+01	2.5433E+01	2.3476E+01
	Rank	8	3	5	7	4	2	6	1
F11	Best	3.2897E+06	3.0954E+05	1.3256E+06	3.2750E+06	8.0092E+05	1.1176E+05	3.5717E+06	4.1685E+05
	Avg	1.9070E+07	3.4831E+06	8.9218E+06	1.9189E+07	3.6736E+06	1.9187E+06	1.2077E+07	1.4532E+06
	Std	1.7785E+07	4.0717E+06	5.5327E+06	1.3479E+07	2.6011E+06	2.1763E+06	4.6337E+06	6.1274E+05
	Rank	7	3	5	8	4	2	6	1
F12	Best	5.3195E+04	1.4138E+04	1.9179E+04	2.7354E+04	1.5613E+04	6.5060E+03	2.5756E+04	6.6651E+03
	Avg	2.2965E+05	3.8035E+04	7.6089E+04	1.3986E+05	2.7183E+04	2.3169E+04	5.2734E+04	1.7523E+04
	Std	1.6260E+05	1.7286E+04	5.0305E+04	7.6957E+04	7.0779E+03	1.2652E+04	3.4545E+04	6.3923E+03
	Rank	8	4	6	7	3	2	5	1
F13	Best	6.2942E+03	1.5174E+03	4.3398E+03	4.5272E+03	1.5259E+03	1.5007E+03	3.5765E+03	1.4710E+03
	Avg	5.5110E+04	1.6120E+03	4.5846E+04	5.4210E+04	1.5618E+03	1.5525E+03	4.8513E+04	1.5007E+03
	Std	4.3914E+04	1.7311E+02	4.0661E+04	4.6535E+04	1.6792E+01	2.5674E+01	4.4461E+04	1.6259E+01
	Rank	8	4	5	7	3	2	6	1
F14	Best	1.0383E+04	2.5935E+03	5.0735E+03	1.6286E+04	2.5322E+03	2.7334E+03	5.9796E+03	1.7322E+03
	Avg	6.5643E+04	1.0960E+04	1.6113E+04	3.8189E+04	4.3868E+03	5.6913E+03	1.1580E+04	2.2916E+03
	Std	4.3771E+04	7.9631E+03	1.0006E+04	1.9599E+04	1.3352E+03	3.3484E+03	5.3028E+03	4.1223E+02
	Rank	8	4	6	7	2	3	5	1
F15	Best	1.8874E+03	1.9167E+03	1.9740E+03	2.1321E+03	1.8599E+03	1.6787E+03	2.2235E+03	1.9480E+03
	Avg	2.6901E+03	2.5520E+03	2.4287E+03	2.5247E+03	2.5029E+03	2.4538E+03	2.6101E+03	2.4857E+03
	Std	3.7575E+02	3.1657E+02	2.6057E+02	2.2668E+02	3.0347E+02	3.6459E+02	2.0323E+02	3.0596E+02
	Rank	8	6	1	5	4	2	7	3
F16	Best	1.8151E+03	1.7559E+03	1.7657E+03	1.8078E+03	1.7636E+03	1.7601E+03	1.7823E+03	1.7644E+03
	Avg	2.0959E+03	1.9748E+03	1.9149E+03	2.0537E+03	1.9031E+03	1.9672E+03	1.9900E+03	1.8781E+03
	Std	1.7482E+02	1.5003E+02	1.1000E+02	1.6341E+02	1.2890E+02	1.5119E+02	1.5989E+02	8.9640E+01
	Rank	8	5	3	7	2	4	6	1
F17	Best	7.9874E+04	3.1765E+03	5.4288E+04	3.8096E+04	5.8222E+03	3.3922E+03	5.1729E+04	2.0838E+03
	Avg	1.2517E+06	1.6802E+04	7.8089E+05	1.0291E+06	1.2426E+04	1.0819E+04	3.3835E+05	4.0202E+03
	Std	1.0054E+06	7.9836E+03	8.7478E+05	1.2380E+06	4.5231E+03	5.1421E+03	2.4257E+05	2.0100E+03
	Rank	8	4	6	7	3	2	5	1
F18	Best	1.3328E+04	2.2430E+03	3.8006E+03	8.2813E+03	2.4028E+03	2.0852E+03	6.3614E+03	2.0235E+03
	Avg	3.4982E+05	1.0441E+04	3.7439E+04	1.5445E+05	5.6001E+03	9.0015E+03	7.8918E+04	3.3183E+03
	Std	3.4980E+05	1.0367E+04	3.3873E+04	2.3581E+05	2.0195E+03	8.8017E+03	9.2874E+04	1.4974E+03
	Rank	8	4	5	7	2	3	6	1
F19	Best	2.1877E+03	2.0812E+03	2.1060E+03	2.1681E+03	2.2184E+03	2.1308E+03	2.2235E+03	2.1136E+03
	Avg	2.4353E+03	2.3402E+03	2.3653E+03	2.3843E+03	2.2715E+03	2.3453E+03	2.2858E+03	2.2479E+03
	Std	1.3278E+02	1.7278E+02	1.6179E+02	1.2088E+02	6.7670E+01	1.5257E+02	6.2661E+01	5.0813E+01
	Rank	8	4	6	7	2	5	3	1
F20	Best	2.3659E+03	2.3399E+03	2.3355E+03	2.3421E+03	2.3271E+03	2.3436E+03	2.3267E+03	2.3195E+03
	Avg	2.3986E+03	2.3706E+03	2.3530E+03	2.3713E+03	2.3520E+03	2.3658E+03	2.3528E+03	2.3386E+03
	Std	1.7502E+01	1.5365E+01	1.1063E+01	1.6503E+01	1.8467E+01	1.3308E+01	1.5381E+01	1.9274E+01
	Rank	8	6	4	7	2	5	3	1
F21	Best	2.3205E+03	2.3121E+03	2.3160E+03	2.3175E+03	2.3106E+03	2.3134E+03	2.3143E+03	2.3025E+03
	Avg	3.4742E+03	2.8099E+03	2.3236E+03	3.0801E+03	2.3166E+03	2.6857E+03	2.3261E+03	2.3085E+03
	Std	1.8013E+03	1.2801E+03	4.6785E+00	1.5543E+03	4.4453E+00	1.1447E+03	7.4730E+00	3.2937E+00
	Rank	8	6	3	7	2	5	4	1
F22	Best	2.7124E+03	2.6897E+03	2.6849E+03	2.6892E+03	2.6816E+03	2.6884E+03	2.6954E+03	2.6729E+03
	Avg	2.7568E+03	2.7301E+03	2.7120E+03	2.7378E+03	2.7065E+03	2.7217E+03	2.7202E+03	2.6954E+03
	Std	2.4505E+01	2.2625E+01	1.7172E+01	2.4331E+01	1.7052E+01	1.9403E+01	1.3168E+01	9.5088E+00
	Rank	8	6	3	7	2	5	4	1
F23	Best	2.8690E+03	2.8480E+03	2.8519E+03	2.8605E+03	2.8376E+03	2.8560E+03	2.8493E+03	2.8353E+03
	Avg	2.9117E+03	2.8914E+03	2.8801E+03	2.8969E+03	2.8671E+03	2.8909E+03	2.8683E+03	2.8578E+03
	Std	2.2493E+01	2.5171E+01	1.5765E+01	1.9783E+01	1.9307E+01	1.9629E+01	1.3424E+01	1.3832E+01
	Rank	8	6	4	7	2	5	3	1
F24	Best	2.8943E+03	2.8889E+03	2.8890E+03	2.8907E+03	2.8988E+03	2.8901E+03	2.9298E+03	2.9025E+03
	Avg	2.9232E+03	2.9169E+03	2.9184E+03	2.9223E+03	2.9351E+03	2.9111E+03	2.9485E+03	2.9293E+03
	Std	2.7158E+01	1.7576E+01	1.6170E+01	1.9699E+01	1.4970E+01	2.0488E+01	9.5033E+00	9.6698E+00
	Rank	5	2	3	4	7	1	8	6
F25	Best	4.2530E+03	4.0580E+03	2.8843E+03	3.1042E+03	3.9276E+03	4.0402E+03	2.9118E+03	2.8244E+03
	Avg	4.7937E+03	4.3811E+03	4.2585E+03	4.5321E+03	4.3544E+03	4.4033E+03	4.6958E+03	4.1381E+03
	Std	2.5309E+02	1.9582E+02	3.2276E+02	4.0317E+02	3.4911E+02	2.1379E+02	7.0553E+02	3.8884E+02
	Rank	8	4	2	6	3	5	7	1
F26	Best	3.2126E+03	3.2132E+03	3.2115E+03	3.2169E+03	3.2312E+03	3.2080E+03	3.2274E+03	3.2275E+03
	Avg	3.2350E+03	3.2371E+03	3.2274E+03	3.2346E+03	3.2449E+03	3.2303E+03	3.2499E+03	3.2394E+03
	Std	1.3741E+01	1.4185E+01	8.5678E+00	1.0896E+01	8.9307E+00	1.3456E+01	1.0848E+01	7.6155E+00
	Rank	4	5	1	3	7	2	8	6
F27	Best	3.2268E+03	3.2257E+03	3.2418E+03	3.2620E+03	3.2302E+03	3.2226E+03	3.2839E+03	3.2372E+03
	Avg	3.3045E+03	3.2867E+03	3.3026E+03	3.3160E+03	3.2989E+03	3.2790E+03	3.3128E+03	3.2901E+03
	Std	4.6372E+01	3.6571E+01	2.1287E+01	4.1564E+01	1.9186E+01	2.5438E+01	1.6709E+01	1.3314E+01
	Rank	6	2	5	8	4	1	7	3
F28	Best	3.5081E+03	3.4715E+03	3.4554E+03	3.5361E+03	3.4819E+03	3.4627E+03	3.5292E+03	3.4884E+03
	Avg	3.8439E+03	3.7374E+03	3.6655E+03	3.8386E+03	3.6428E+03	3.7199E+03	3.7652E+03	3.5727E+03
	Std	1.8650E+02	1.4860E+02	1.5881E+02	1.7895E+02	1.3438E+02	1.7171E+02	1.4886E+02	7.9040E+01
	Rank	8	5	3	7	2	4	6	1
F29	Best	2.3332E+05	2.4081E+04	2.2046E+05	2.5097E+05	2.3033E+04	1.6590E+04	3.0036E+05	1.4092E+04
	Avg	2.4397E+06	1.5570E+05	8.7634E+05	2.1945E+06	7.2225E+04	7.0292E+04	3.0593E+06	2.3200E+04
	Std	2.0619E+06	1.5443E+05	5.8123E+05	1.3247E+06	5.2229E+04	9.5801E+04	1.3977E+06	9.4801E+03
	Rank	7	4	5	6	3	2	8	1

**Table A3:** Comparison of DRIME with different strategies in CEC-2017 test suite (D = 50).

No.	Index	RIME	DRIME-D	DRIME-S	DRIME-H	DRIME-DS	DRIME-DH	DRIME-SH	DRIME
F1	Best	6.8842E+07	1.1229E+07	2.6951E+07	4.8922E+07	1.7144E+07	1.3659E+07	5.9938E+07	4.0061E+06
	Avg	1.6944E+08	4.9376E+07	7.9082E+07	1.3133E+08	6.3718E+07	3.9428E+07	1.6596E+08	2.0985E+07
	Std	7.6863E+07	2.6791E+07	3.5014E+07	5.6907E+07	4.3985E+07	2.0648E+07	8.4964E+07	1.2195E+07
	Rank	8	3	5	6	4	2	7	1
F2	Best	5.6999E+04	1.4024E+04	5.7187E+04	5.1857E+04	1.0705E+04	9.0775E+03	7.0485E+04	6.4166E+03
	Avg	1.0342E+05	2.1710E+04	8.2396E+04	9.0327E+04	1.8606E+04	2.0696E+04	9.2906E+04	1.1025E+04
	Std	2.6705E+04	6.1452E+03	1.3124E+04	1.8187E+04	4.7448E+03	4.5363E+03	9.8947E+03	2.7665E+03
	Rank	8	4	5	6	2	3	7	1
F3	Best	5.0124E+02	5.4367E+02	5.9847E+02	5.8217E+02	6.2434E+02	5.5332E+02	6.7983E+02	6.1259E+02
	Avg	6.7392E+02	6.5097E+02	6.5763E+02	6.6400E+02	6.9777E+02	6.5509E+02	7.2854E+02	6.6641E+02
	Std	7.0428E+01	5.9318E+01	4.5162E+01	4.0517E+01	3.3749E+01	4.2226E+01	3.0955E+01	2.3270E+01
	Rank	6	1	3	4	7	2	8	5
F4	Best	6.1377E+02	5.8455E+02	5.8051E+02	6.1549E+02	5.6834E+02	5.8311E+02	6.1142E+02	5.6453E+02
	Avg	7.0896E+02	6.3644E+02	6.2185E+02	6.5131E+02	6.0555E+02	6.2273E+02	6.3998E+02	5.9621E+02
	Std	4.7270E+01	2.9374E+01	2.7384E+01	2.5404E+01	2.2218E+01	2.2991E+01	1.6078E+01	1.6224E+01
	Rank	8	5	3	7	2	4	6	1
F5	Best	6.0966E+02	6.0340E+02	6.0303E+02	6.0649E+02	6.0417E+02	6.0243E+02	6.0664E+02	6.0127E+02
	Avg	6.1745E+02	6.0641E+02	6.0603E+02	6.1379E+02	6.0910E+02	6.0664E+02	6.1462E+02	6.0404E+02
	Std	4.9970E+00	2.6165E+00	2.0779E+00	3.7287E+00	2.7240E+00	3.4057E+00	3.9966E+00	1.3802E+00
	Rank	8	3	2	6	5	4	7	1
F6	Best	9.7473E+02	9.0287E+02	8.9254E+02	9.6701E+02	8.7195E+02	8.9040E+02	8.9257E+02	8.3515E+02
	Avg	1.1025E+03	9.6137E+02	9.6839E+02	1.0447E+03	9.5689E+02	9.4862E+02	9.8966E+02	9.2051E+02
	Std	5.8103E+01	3.6185E+01	3.5425E+01	4.8305E+01	4.4980E+01	3.3563E+01	4.3218E+01	4.2875E+01
	Rank	8	4	5	7	3	2	6	1
F7	Best	9.7874E+02	8.8096E+02	8.8617E+02	9.1852E+02	8.6805E+02	8.6989E+02	8.9683E+02	8.6561E+02
	Avg	1.0211E+03	9.3426E+02	9.2826E+02	9.7468E+02	9.0749E+02	9.2144E+02	9.4691E+02	8.9997E+02
	Std	2.8451E+01	2.7876E+01	2.3561E+01	3.1959E+01	2.4293E+01	3.3034E+01	2.3190E+01	2.5593E+01
	Rank	8	5	4	7	2	3	6	1
F8	Best	2.3410E+03	9.9473E+02	1.1502E+03	1.5894E+03	1.2954E+03	1.0378E+03	1.7011E+03	1.0340E+03
	Avg	4.7837E+03	1.2545E+03	1.6265E+03	3.2937E+03	1.6685E+03	1.3178E+03	2.7659E+03	1.2891E+03
	Std	2.0008E+03	1.8262E+02	3.7879E+02	1.2221E+03	2.7017E+02	2.1971E+02	5.4356E+02	2.0886E+02
	Rank	8	1	4	7	5	3	6	2
F9	Best	6.9718E+03	6.0779E+03	6.3990E+03	7.0923E+03	5.2324E+03	6.1550E+03	6.2358E+03	6.0615E+03
	Avg	8.4727E+03	8.0093E+03	8.1804E+03	8.3135E+03	7.9582E+03	7.6611E+03	8.1973E+03	7.5995E+03
	Std	7.1243E+02	6.8480E+02	8.1634E+02	9.2031E+02	1.0785E+03	9.9074E+02	9.6119E+02	9.6850E+02
	Rank	8	4	5	7	3	2	6	1
F10	Best	1.5596E+03	1.2771E+03	1.3439E+03	1.4571E+03	1.3068E+03	1.2982E+03	1.4769E+03	1.2720E+03
	Avg	1.7438E+03	1.3643E+03	1.5450E+03	1.6190E+03	1.3997E+03	1.3586E+03	1.7139E+03	1.3273E+03
	Std	1.3661E+02	4.2645E+01	8.5326E+01	1.2086E+02	4.9182E+01	3.2250E+01	9.6210E+01	4.0439E+01
	Rank	8	3	5	6	4	2	7	1
F11	Best	2.5340E+07	4.2343E+06	9.2450E+06	1.3893E+07	1.8414E+07	3.9642E+06	5.0953E+07	3.9022E+06
	Avg	1.6119E+08	2.6636E+07	6.4051E+07	1.1900E+08	4.8430E+07	2.1478E+07	1.1918E+08	1.7893E+07
	Std	9.4393E+07	2.0104E+07	3.1884E+07	7.0784E+07	2.3545E+07	1.5136E+07	3.3639E+07	9.2710E+06
	Rank	8	3	5	6	4	2	7	1
F12	Best	1.7622E+05	3.9324E+04	8.3632E+04	1.5958E+05	2.4203E+04	1.3478E+04	1.2649E+05	1.5808E+04
	Avg	8.6703E+05	7.8250E+04	3.9502E+05	7.0257E+05	4.3286E+04	3.0531E+04	3.6808E+05	2.0819E+04
	Std	5.4712E+05	2.5628E+04	2.7333E+05	4.6678E+05	1.4136E+04	9.8550E+03	2.8331E+05	3.6439E+03
	Rank	8	4	6	7	3	2	5	1
F13	Best	1.4859E+04	1.7043E+03	5.9307E+04	1.0732E+05	1.8393E+03	1.7197E+03	9.1187E+04	1.5816E+03
	Avg	3.4422E+05	2.4598E+03	3.2347E+05	3.1018E+05	2.0704E+03	2.2215E+03	2.8552E+05	1.6520E+03
	Std	2.3876E+05	1.0667E+03	1.8652E+05	1.1861E+05	2.1865E+02	7.7410E+02	1.2982E+05	4.2203E+01
	Rank	8	4	7	6	2	3	5	1
F14	Best	5.9325E+04	5.4444E+03	1.4716E+04	3.1202E+04	6.0818E+03	4.8916E+03	1.9496E+04	3.7229E+03
	Avg	1.6172E+05	1.7407E+04	5.7953E+04	1.2734E+05	1.3251E+04	1.2218E+04	7.2578E+04	6.9619E+03
	Std	7.5990E+04	6.4615E+03	3.5581E+04	7.9048E+04	4.8655E+03	6.2291E+03	6.5924E+04	1.6229E+03
	Rank	8	4	5	7	3	2	6	1
F15	Best	2.8888E+03	2.3568E+03	2.1413E+03	2.8750E+03	2.3318E+03	2.4614E+03	2.3269E+03	2.1112E+03
	Avg	3.5722E+03	3.3121E+03	2.8044E+03	3.3146E+03	2.7016E+03	3.0659E+03	2.7828E+03	2.5272E+03
	Std	4.5475E+02	4.0429E+02	4.0448E+02	2.9803E+02	2.0846E+02	3.6727E+02	3.1264E+02	2.6810E+02
	Rank	8	6	4	7	2	5	3	1
F16	Best	2.4104E+03	2.1830E+03	2.2797E+03	2.3834E+03	2.2480E+03	2.1528E+03	2.3025E+03	2.2575E+03
	Avg	3.1609E+03	2.7990E+03	2.8522E+03	2.9706E+03	2.7490E+03	2.7837E+03	2.8759E+03	2.7246E+03
	Std	3.3127E+02	2.7240E+02	3.0014E+02	3.1538E+02	2.6557E+02	2.8475E+02	2.4462E+02	2.2001E+02
	Rank	8	4	5	7	2	3	6	1
F17	Best	2.0622E+05	1.8308E+04	2.2999E+05	4.2525E+05	1.4246E+04	1.2619E+04	6.4348E+05	3.6396E+03
	Avg	3.1300E+06	3.9390E+04	2.2081E+06	3.5798E+06	2.4992E+04	3.1249E+04	2.0300E+06	8.3535E+03
	Std	2.4282E+06	1.6947E+04	1.4597E+06	2.4560E+06	6.2697E+03	1.3418E+04	6.2573E+05	4.5065E+03
	Rank	7	4	6	8	2	3	5	1
F18	Best	7.6775E+04	4.7780E+03	1.3249E+04	4.7473E+04	1.3799E+04	3.5611E+03	5.2486E+04	1.0285E+04
	Avg	1.2091E+06	2.5676E+04	6.4263E+04	7.9577E+05	2.3370E+04	1.5497E+04	1.4870E+05	1.6155E+04
	Std	1.0279E+06	1.4872E+04	3.2194E+04	8.2275E+05	6.2811E+03	6.9362E+03	5.6727E+04	2.9681E+03
	Rank	8	4	5	7	3	1	6	2
F19	Best	2.4917E+03	2.4222E+03	2.2993E+03	2.5340E+03	2.2420E+03	2.6339E+03	2.3514E+03	2.2472E+03
	Avg	3.0445E+03	2.8730E+03	2.8522E+03	2.9920E+03	2.7611E+03	2.9723E+03	2.8210E+03	2.6364E+03
	Std	3.1228E+02	2.6321E+02	2.7514E+02	2.2737E+02	2.6063E+02	2.0337E+02	3.1726E+02	2.3806E+02
	Rank	8	5	4	7	2	6	3	1
F20	Best	2.4708E+03	2.3773E+03	2.3695E+03	2.4228E+03	2.3555E+03	2.3822E+03	2.3876E+03	2.3532E+03
	Avg	2.5238E+03	2.4354E+03	2.4197E+03	2.4626E+03	2.4005E+03	2.4259E+03	2.4298E+03	2.3836E+03
	Std	3.2690E+01	2.9704E+01	2.4794E+01	2.0295E+01	2.9132E+01	2.2537E+01	2.1438E+01	2.0439E+01
	Rank	8	6	3	7	2	4	5	1
F21	Best	7.9296E+03	7.5058E+03	7.8433E+03	7.4386E+03	2.3687E+03	6.3818E+03	2.3789E+03	2.3353E+03
	Avg	1.0109E+04	9.7136E+03	9.7054E+03	9.7030E+03	8.2635E+03	9.0114E+03	9.5690E+03	8.2025E+03
	Std	9.8119E+02	1.0575E+03	1.0090E+03	1.0186E+03	3.3855E+03	8.9966E+02	2.6488E+03	3.2379E+03
	Rank	8	7	6	5	2	3	4	1
F22	Best	2.8446E+03	2.8331E+03	2.8244E+03	2.8472E+03	2.8197E+03	2.8466E+03	2.8300E+03	2.7913E+03
	Avg	2.9741E+03	2.9050E+03	2.8696E+03	2.9342E+03	2.8692E+03	2.8980E+03	2.8907E+03	2.8347E+03
	Std	6.2520E+01	4.4425E+01	3.1244E+01	5.2863E+01	3.5649E+01	2.4428E+01	2.5956E+01	2.3233E+01
	Rank	8	6	3	7	2	5	4	1
F23	Best	3.0060E+03	3.0147E+03	2.9837E+03	3.0093E+03	2.9583E+03	2.9927E+03	2.9820E+03	2.9502E+03
	Avg	3.0981E+03	3.0803E+03	3.0278E+03	3.0650E+03	3.0132E+03	3.0556E+03	3.0365E+03	2.9809E+03
	Std	4.4117E+01	4.0485E+01	2.2892E+01	3.9217E+01	2.9039E+01	3.4204E+01	3.0245E+01	1.7682E+01
	Rank	8	7	3	6	2	5	4	1
F24	Best	3.0794E+03	3.0407E+03	3.0779E+03	3.0908E+03	3.1513E+03	3.0719E+03	3.1712E+03	3.1392E+03
	Avg	3.1591E+03	3.1248E+03	3.1759E+03	3.1736E+03	3.2116E+03	3.1234E+03	3.2597E+03	3.1865E+03
	Std	4.3522E+01	3.2373E+01	4.4457E+01	5.2837E+01	3.4061E+01	3.8251E+01	4.3347E+01	2.8716E+01
	Rank	3	2	5	4	7	1	8	6
F25	Best	5.4970E+03	4.9030E+03	4.7098E+03	5.1428E+03	4.7188E+03	4.4625E+03	5.7398E+03	4.8458E+03
	Avg	6.2197E+03	5.2969E+03	5.2194E+03	5.7559E+03	5.4517E+03	5.2209E+03	6.5919E+03	5.2630E+03
	Std	4.3409E+02	3.0327E+02	2.8945E+02	3.3792E+02	3.4429E+02	2.7085E+02	6.4049E+02	1.9879E+02
	Rank	7	4	1	6	5	2	8	3
F26	Best	3.3828E+03	3.3615E+03	3.3679E+03	3.3911E+03	3.4594E+03	3.3698E+03	3.4829E+03	3.3985E+03
	Avg	3.4677E+03	3.4733E+03	3.4409E+03	3.4955E+03	3.5382E+03	3.4642E+03	3.5947E+03	3.4901E+03
	Std	5.0072E+01	6.2015E+01	3.9575E+01	8.0240E+01	4.2303E+01	5.5016E+01	6.5057E+01	4.4434E+01
	Rank	3	4	1	6	7	2	8	5
F27	Best	3.3303E+03	3.3782E+03	3.4057E+03	3.3569E+03	3.4844E+03	3.3644E+03	3.5166E+03	3.4371E+03
	Avg	3.4680E+03	3.4689E+03	3.4976E+03	3.4896E+03	3.6221E+03	3.4715E+03	3.6518E+03	3.5491E+03
	Std	7.4788E+01	5.7980E+01	6.7463E+01	7.7676E+01	6.9326E+01	5.1127E+01	6.9556E+01	5.0592E+01
	Rank	1	2	5	4	7	3	8	6
F28	Best	3.9736E+03	3.6380E+03	3.8659E+03	3.9180E+03	3.8510E+03	3.8954E+03	4.1923E+03	3.8499E+03
	Avg	4.6282E+03	4.2313E+03	4.2389E+03	4.5937E+03	4.3210E+03	4.3048E+03	4.5727E+03	4.1930E+03
	Std	3.5122E+02	3.2531E+02	2.5734E+02	3.6678E+02	2.5488E+02	2.7314E+02	2.0024E+02	1.5125E+02
	Rank	8	2	3	7	5	4	6	1
F29	Best	3.5875E+07	1.4594E+07	1.7073E+07	3.4513E+07	3.1068E+07	8.0112E+06	7.3721E+07	1.1161E+07
	Avg	5.8634E+07	2.4029E+07	3.4440E+07	6.2055E+07	4.9682E+07	1.3578E+07	1.0282E+08	2.1554E+07
	Std	1.7764E+07	7.5368E+06	9.6168E+06	1.7402E+07	1.2143E+07	3.5107E+06	1.6889E+07	6.1361E+06
	Rank	6	3	4	7	5	1	8	2

**Table A4:** Comparison of DRIME with different strategies in CEC-2017 test suite (D = 100).

No.	Index	RIME	DRIME-D	DRIME-S	DRIME-H	DRIME-DS	DRIME-DH	DRIME-SH	DRIME
F1	Best	8.9770E+08	4.1054E+08	4.8701E+08	7.3966E+08	4.3739E+08	4.8051E+08	1.0262E+09	1.8597E+08
	Avg	1.5648E+09	7.2491E+08	7.8224E+08	1.2931E+09	1.0680E+09	7.3183E+08	1.7410E+09	4.5502E+08
	Std	3.8504E+08	1.7556E+08	1.9183E+08	3.2408E+08	4.3808E+08	2.4677E+08	5.6854E+08	2.1106E+08
	Rank	7	2	4	6	5	3	8	1
F2	Best	3.0629E+05	8.1425E+04	2.0672E+05	2.3581E+05	6.5277E+04	1.0826E+05	2.1051E+05	5.4624E+04
	Avg	3.7345E+05	1.3799E+05	2.6222E+05	3.3492E+05	8.7971E+04	1.4853E+05	2.5284E+05	7.0773E+04
	Std	4.2162E+04	2.4849E+04	2.6697E+04	4.8400E+04	1.1204E+04	2.4280E+04	1.7051E+04	9.0094E+03
	Rank	8	3	6	7	2	4	5	1
F3	Best	9.2913E+02	9.1827E+02	9.8009E+02	9.3543E+02	1.0122E+03	8.9736E+02	1.0644E+03	9.2033E+02
	Avg	1.1140E+03	1.0294E+03	1.0647E+03	1.1251E+03	1.1100E+03	1.0331E+03	1.2355E+03	1.0414E+03
	Std	8.4048E+01	6.3630E+01	6.4550E+01	1.0056E+02	6.2417E+01	7.4486E+01	8.4769E+01	6.8193E+01
	Rank	6	1	4	7	5	2	8	3
F4	Best	9.8405E+02	8.0246E+02	7.8648E+02	8.8861E+02	7.8909E+02	7.9250E+02	9.1442E+02	7.6337E+02
	Avg	1.1105E+03	8.9929E+02	8.8522E+02	1.0171E+03	8.8904E+02	8.9459E+02	9.8371E+02	8.3476E+02
	Std	7.8946E+01	4.9735E+01	4.4703E+01	7.5043E+01	5.4520E+01	5.4467E+01	3.8567E+01	5.5361E+01
	Rank	8	5	2	7	3	4	6	1
F5	Best	6.2116E+02	6.0916E+02	6.1195E+02	6.2030E+02	6.1376E+02	6.1071E+02	6.2441E+02	6.0875E+02
	Avg	6.3233E+02	6.1635E+02	6.1594E+02	6.2817E+02	6.2152E+02	6.1568E+02	6.3208E+02	6.1504E+02
	Std	5.3855E+00	3.6484E+00	3.0324E+00	3.8997E+00	3.9459E+00	2.9474E+00	3.8124E+00	2.8775E+00
	Rank	8	4	3	6	5	2	7	1
F6	Best	1.7638E+03	1.3104E+03	1.3360E+03	1.5797E+03	1.2303E+03	1.2509E+03	1.4285E+03	1.2401E+03
	Avg	1.8845E+03	1.4485E+03	1.4985E+03	1.7524E+03	1.4676E+03	1.3865E+03	1.6200E+03	1.3717E+03
	Std	8.2051E+01	7.7140E+01	6.6815E+01	9.5638E+01	1.0628E+02	6.0128E+01	8.5829E+01	8.2306E+01
	Rank	8	3	5	7	4	2	6	1
F7	Best	1.2700E+03	1.1258E+03	1.1292E+03	1.2210E+03	1.1042E+03	1.1296E+03	1.2288E+03	1.0340E+03
	Avg	1.4183E+03	1.2119E+03	1.1946E+03	1.3285E+03	1.2038E+03	1.2081E+03	1.2967E+03	1.1217E+03
	Std	8.4831E+01	4.8780E+01	4.6168E+01	6.2342E+01	4.8860E+01	4.9272E+01	3.8791E+01	4.1154E+01
	Rank	8	5	2	7	3	4	6	1
F8	Best	1.0631E+04	2.6509E+03	4.4201E+03	9.0533E+03	4.7367E+03	3.1090E+03	1.3128E+04	4.3350E+03
	Avg	2.2456E+04	5.0535E+03	7.7561E+03	1.8042E+04	9.5806E+03	4.4571E+03	1.6651E+04	6.6472E+03
	Std	7.8428E+03	1.4373E+03	2.0313E+03	5.2229E+03	2.3857E+03	1.0624E+03	2.1085E+03	1.1423E+03
	Rank	8	2	4	7	5	1	6	3
F9	Best	1.7015E+04	1.5835E+04	1.4934E+04	1.6093E+04	1.5225E+04	1.5052E+04	1.6698E+04	1.4964E+04
	Avg	1.9568E+04	1.9267E+04	1.8290E+04	1.8666E+04	2.1158E+04	1.8749E+04	1.9809E+04	1.8742E+04
	Std	1.2078E+03	1.6163E+03	1.4406E+03	1.6826E+03	3.3141E+03	1.6845E+03	2.1039E+03	2.5902E+03
	Rank	6	5	1	2	8	4	7	3
F10	Best	6.4350E+03	2.6082E+03	6.5749E+03	6.5748E+03	3.3022E+03	2.6927E+03	1.1361E+04	2.6623E+03
	Avg	1.0324E+04	3.5106E+03	1.0568E+04	9.9497E+03	3.9388E+03	3.2575E+03	1.8125E+04	3.1655E+03
	Std	2.3534E+03	3.6694E+02	2.4258E+03	1.9364E+03	4.0055E+02	3.4538E+02	3.1061E+03	2.9751E+02
	Rank	6	3	7	5	4	2	8	1
F11	Best	2.9446E+08	1.4715E+08	2.9286E+08	2.8080E+08	4.3340E+08	1.5534E+08	4.8213E+08	3.3608E+08
	Avg	7.9315E+08	4.3223E+08	5.5190E+08	7.6233E+08	5.6918E+08	3.6312E+08	6.7380E+08	4.3000E+08
	Std	3.0286E+08	1.8430E+08	1.4377E+08	3.0241E+08	1.1797E+08	1.5322E+08	9.7163E+07	4.9462E+07
	Rank	8	3	4	7	5	1	6	2
F12	Best	1.3984E+06	6.0985E+04	4.8624E+05	6.9845E+05	3.4275E+04	4.1750E+04	3.2828E+05	1.8665E+04
	Avg	4.2650E+06	1.2603E+05	1.2447E+06	2.7896E+06	6.0901E+04	8.3238E+04	1.6683E+06	2.3730E+04
	Std	4.1321E+06	4.3398E+04	6.1621E+05	1.5377E+06	5.3372E+04	3.0701E+04	1.0668E+06	3.1901E+03
	Rank	8	4	5	7	2	3	6	1
F13	Best	1.4184E+06	1.1947E+04	1.2974E+06	1.5213E+06	5.3429E+03	9.1460E+03	2.8930E+06	2.0737E+03
	Avg	5.1483E+06	3.0093E+04	3.5269E+06	4.4013E+06	1.8792E+04	2.3127E+04	4.2129E+06	3.6614E+03
	Std	2.4045E+06	1.6807E+04	1.3065E+06	2.3299E+06	8.8927E+03	1.1042E+04	7.1744E+05	2.4276E+03
	Rank	8	4	5	7	2	3	6	1
F14	Best	1.4018E+05	1.7003E+04	5.7279E+04	1.5196E+05	1.6092E+04	8.7381E+03	4.4947E+04	1.0036E+04
	Avg	7.0962E+05	2.8459E+05	2.0193E+05	4.8666E+05	2.1733E+04	1.6700E+04	2.4432E+05	1.2583E+04
	Std	4.6458E+05	1.3528E+06	1.2768E+05	2.9531E+05	3.0188E+03	5.2715E+03	1.9855E+05	1.3319E+03
	Rank	8	6	4	7	3	2	5	1
F15	Best	4.7109E+03	4.3822E+03	3.8354E+03	3.9894E+03	4.3096E+03	4.4412E+03	4.5370E+03	3.3743E+03
	Avg	6.1292E+03	5.9358E+03	5.4679E+03	6.0565E+03	5.2928E+03	5.6363E+03	5.8111E+03	5.1112E+03
	Std	8.1024E+02	7.5826E+02	7.7912E+02	7.7983E+02	5.0896E+02	6.5913E+02	5.6480E+02	6.0194E+02
	Rank	8	6	3	7	2	4	5	1
F16	Best	4.4819E+03	3.7651E+03	3.8030E+03	3.9451E+03	3.4557E+03	3.6036E+03	3.6302E+03	2.8039E+03
	Avg	5.2664E+03	4.7229E+03	4.4207E+03	4.9875E+03	4.2084E+03	4.5575E+03	4.4310E+03	4.0253E+03
	Std	4.1898E+02	4.3555E+02	3.7812E+02	5.2234E+02	4.0321E+02	5.0020E+02	3.8993E+02	4.0261E+02
	Rank	8	6	3	7	2	5	4	1
F17	Best	2.7467E+06	5.2545E+04	1.3980E+06	1.2959E+06	5.1301E+04	5.1064E+04	1.9984E+06	1.7350E+04
	Avg	8.1556E+06	1.0268E+05	3.3888E+06	5.9812E+06	7.0415E+04	7.7756E+04	3.1128E+06	3.6221E+04
	Std	3.4667E+06	3.0172E+04	1.1656E+06	2.9122E+06	1.2779E+04	1.9685E+04	5.6074E+05	1.3042E+04
	Rank	8	4	6	7	2	3	5	1
F18	Best	2.3432E+06	6.9368E+04	1.7372E+05	1.7845E+06	1.6439E+05	1.7922E+04	5.7161E+05	4.1133E+04
	Avg	1.2306E+07	3.3961E+05	5.2294E+05	5.9457E+06	3.7152E+05	5.4707E+04	1.1542E+06	1.3806E+05
	Std	6.4231E+06	3.2416E+05	3.0877E+05	2.8307E+06	1.8326E+05	2.5200E+04	3.6885E+05	6.3792E+04
	Rank	8	3	5	7	4	1	6	2
F19	Best	4.5594E+03	4.1911E+03	3.8849E+03	4.5130E+03	3.9301E+03	3.2561E+03	3.9140E+03	3.1375E+03
	Avg	5.3192E+03	4.9463E+03	4.7914E+03	5.2042E+03	4.6470E+03	4.8143E+03	4.6079E+03	4.3069E+03
	Std	4.7803E+02	4.1678E+02	4.5632E+02	3.1962E+02	3.7665E+02	5.8348E+02	4.8795E+02	5.0682E+02
	Rank	8	6	4	7	3	5	2	1
F20	Best	2.8427E+03	2.6144E+03	2.6298E+03	2.7293E+03	2.5850E+03	2.6583E+03	2.6604E+03	2.5424E+03
	Avg	2.9663E+03	2.7378E+03	2.6996E+03	2.8651E+03	2.6886E+03	2.7335E+03	2.7546E+03	2.5954E+03
	Std	6.5904E+01	5.9858E+01	3.9706E+01	7.1787E+01	7.0904E+01	4.3024E+01	4.8331E+01	3.5985E+01
	Rank	8	5	3	7	2	4	6	1
F21	Best	1.8244E+04	1.8028E+04	1.6001E+04	1.8001E+04	1.8655E+04	1.8219E+04	1.8058E+04	1.7569E+04
	Avg	2.2003E+04	2.1049E+04	2.1065E+04	2.1703E+04	2.1771E+04	2.1160E+04	2.1374E+04	2.0584E+04
	Std	1.3610E+03	1.6228E+03	2.2546E+03	1.4929E+03	1.8793E+03	1.3331E+03	2.1951E+03	2.2566E+03
	Rank	8	2	3	6	7	4	5	1
F22	Best	3.2334E+03	3.2459E+03	3.1214E+03	3.1846E+03	3.1427E+03	3.2093E+03	3.2161E+03	3.0879E+03
	Avg	3.4149E+03	3.3503E+03	3.1926E+03	3.3459E+03	3.2412E+03	3.3170E+03	3.2921E+03	3.1578E+03
	Std	8.2638E+01	7.3948E+01	4.1678E+01	6.3792E+01	4.9117E+01	6.2955E+01	6.4551E+01	4.3185E+01
	Rank	8	7	2	6	3	5	4	1
F23	Best	3.7678E+03	3.6694E+03	3.5793E+03	3.7846E+03	3.5807E+03	3.7354E+03	3.6546E+03	3.5022E+03
	Avg	3.9611E+03	3.8840E+03	3.7046E+03	3.9113E+03	3.7046E+03	3.8745E+03	3.7854E+03	3.5991E+03
	Std	1.0294E+02	9.0606E+01	5.1293E+01	7.3307E+01	4.8487E+01	8.6730E+01	8.1218E+01	4.3531E+01
	Rank	8	6	2	7	3	5	4	1
F24	Best	3.6044E+03	3.5604E+03	3.6819E+03	3.6252E+03	3.7473E+03	3.6216E+03	3.8089E+03	3.6855E+03
	Avg	3.8823E+03	3.7318E+03	3.7984E+03	3.8561E+03	3.8697E+03	3.7324E+03	3.9671E+03	3.7750E+03
	Std	1.3329E+02	8.5337E+01	5.9844E+01	1.2452E+02	6.8911E+01	8.4161E+01	8.7047E+01	6.6123E+01
	Rank	7	1	4	5	6	2	8	3
F25	Best	1.1328E+04	9.2692E+03	9.2462E+03	1.0567E+04	1.0566E+04	9.3506E+03	1.4335E+04	1.0327E+04
	Avg	1.3047E+04	1.0950E+04	1.0357E+04	1.2314E+04	1.2984E+04	1.1099E+04	1.6992E+04	1.2120E+04
	Std	8.9873E+02	8.2882E+02	5.0322E+02	1.0641E+03	1.1268E+03	7.1887E+02	1.3756E+03	1.1013E+03
	Rank	7	2	1	5	6	3	8	4
F26	Best	3.6059E+03	3.6400E+03	3.5696E+03	3.6594E+03	3.8460E+03	3.5864E+03	3.9704E+03	3.7547E+03
	Avg	3.7678E+03	3.7358E+03	3.6976E+03	3.8352E+03	3.9538E+03	3.7357E+03	4.1126E+03	3.8663E+03
	Std	1.0508E+02	7.0069E+01	7.6044E+01	9.4861E+01	5.4366E+01	9.4377E+01	7.7166E+01	5.7117E+01
	Rank	4	3	1	5	7	2	8	6
F27	Best	3.9053E+03	3.7708E+03	3.9151E+03	3.9002E+03	4.0500E+03	3.7643E+03	4.0666E+03	3.8831E+03
	Avg	4.4116E+03	4.0032E+03	4.0487E+03	4.2211E+03	4.3197E+03	3.9862E+03	4.2964E+03	4.0196E+03
	Std	3.7284E+02	1.7655E+02	1.0137E+02	2.0927E+02	1.3792E+02	1.4358E+02	1.1794E+02	8.2876E+01
	Rank	8	2	4	5	7	1	6	3
F28	Best	7.2055E+03	5.8822E+03	6.2691E+03	6.8154E+03	6.4996E+03	5.5875E+03	7.1913E+03	6.1746E+03
	Avg	8.1762E+03	7.2406E+03	6.9686E+03	7.9736E+03	7.2518E+03	6.8874E+03	8.1596E+03	6.9884E+03
	Std	5.2046E+02	5.8976E+02	4.2836E+02	6.1890E+02	4.3790E+02	5.1168E+02	4.6525E+02	4.4857E+02
	Rank	8	4	2	6	5	1	7	3
F29	Best	3.7308E+07	8.0644E+06	1.6563E+07	3.7415E+07	2.4745E+07	3.3686E+06	5.2336E+07	9.3543E+06
	Avg	9.8516E+07	2.7585E+07	3.4680E+07	1.1485E+08	6.9989E+07	9.9647E+06	9.3678E+07	2.9735E+07
	Std	4.8600E+07	1.7460E+07	1.3125E+07	4.6390E+07	2.7973E+07	4.2379E+06	2.0876E+07	1.0504E+07
	Rank	7	2	4	8	5	1	6	3

**Table A5:** Comparison of DRIME with other RIME variants in CEC-2017 test suite (D = 10).

No.	Index	DRIME	ACGRIME	TERIME	IRIME	DNMRIME	GLSRIME	HERIME
F1	Best	5.1814E+02	3.3067E+05	7.5237E+06	2.2307E+07	2.7128E+05	9.6798E+04	5.4938E+05
	Mean	3.2371E+03	1.2818E+07	5.1310E+07	7.6251E+07	3.0813E+06	6.8640E+05	2.3975E+06
	Std	2.5383E+03	1.6586E+07	3.3615E+07	4.3877E+07	2.7421E+06	4.5168E+05	1.6278E+06
	Rank	1	5	6	7	4	2	3
F2	Best	3.0006E+02	4.6380E+02	1.1004E+03	1.3107E+03	3.8840E+02	3.0677E+02	5.3700E+02
	Mean	3.0034E+02	3.1631E+03	3.5008E+03	6.9038E+03	9.1730E+02	3.7202E+02	9.4899E+02
	Std	2.6805E-01	1.4854E+03	1.7516E+03	3.1164E+03	4.2869E+02	5.9531E+01	3.6599E+02
	Rank	1	5	6	7	3	2	4
F3	Best	4.0448E+02	4.0571E+02	4.0802E+02	4.0858E+02	4.0092E+02	4.0014E+02	4.0326E+02
	Mean	4.0637E+02	4.1359E+02	4.1607E+02	4.1414E+02	4.1460E+02	4.1251E+02	4.0687E+02
	Std	6.6339E-01	1.2472E+01	1.4985E+01	1.1695E+01	2.1446E+01	1.8262E+01	1.6816E+00
	Rank	1	4	7	5	6	3	2
F4	Best	5.0398E+02	5.0606E+02	5.1330E+02	5.2060E+02	5.0738E+02	5.0579E+02	5.0682E+02
	Mean	5.0746E+02	5.2031E+02	5.3550E+02	5.3680E+02	5.2536E+02	5.1786E+02	5.1739E+02
	Std	3.4503E+00	6.9714E+00	1.0886E+01	8.9340E+00	1.0592E+01	6.0282E+00	8.6039E+00
	Rank	1	4	6	7	5	3	2
F5	Best	6.0002E+02	6.0054E+02	6.0513E+02	6.0374E+02	6.0061E+02	6.0032E+02	6.0067E+02
	Mean	6.0008E+02	6.0229E+02	6.1061E+02	6.0704E+02	6.0291E+02	6.0110E+02	6.0153E+02
	Std	5.9760E-02	1.4271E+00	5.1388E+00	2.5475E+00	1.6607E+00	6.1004E-01	5.5189E-01
	Rank	1	4	7	6	5	2	3
F6	Best	7.1247E+02	7.1905E+02	7.4747E+02	7.3911E+02	7.2770E+02	7.1499E+02	7.2436E+02
	Mean	7.1894E+02	7.3829E+02	7.7161E+02	7.6619E+02	7.4097E+02	7.3200E+02	7.3729E+02
	Std	5.8972E+00	1.1978E+01	1.4910E+01	1.1363E+01	9.0340E+00	8.7371E+00	8.9954E+00
	Rank	1	4	7	6	5	2	3
F7	Best	8.0100E+02	8.0734E+02	8.2000E+02	8.1798E+02	8.0808E+02	8.0877E+02	8.0452E+02
	Mean	8.0552E+02	8.1733E+02	8.3943E+02	8.3427E+02	8.2176E+02	8.1940E+02	8.1646E+02
	Std	4.1056E+00	5.6577E+00	1.2212E+01	1.0795E+01	7.2783E+00	7.5120E+00	7.0936E+00
	Rank	1	3	7	6	5	4	2
F8	Best	9.0000E+02	9.0385E+02	9.3837E+02	9.1130E+02	9.0070E+02	9.0013E+02	9.0043E+02
	Mean	9.0001E+02	9.2141E+02	1.0574E+03	9.5783E+02	9.1436E+02	9.0192E+02	9.0348E+02
	Std	2.8392E-02	1.6873E+01	1.3403E+02	3.6822E+01	3.4698E+01	2.3450E+00	3.8248E+00
	Rank	1	5	7	6	4	2	3
F9	Best	1.1443E+03	1.2823E+03	1.6184E+03	1.5535E+03	1.1926E+03	1.1509E+03	1.1851E+03
	Mean	1.5124E+03	1.6859E+03	2.1115E+03	2.0620E+03	1.8057E+03	1.5815E+03	1.7087E+03
	Std	2.4264E+02	1.9338E+02	2.8942E+02	2.8209E+02	3.5364E+02	2.1274E+02	2.3124E+02
	Rank	1	3	7	6	5	2	4
F10	Best	1.1030E+03	1.1134E+03	1.1195E+03	1.1190E+03	1.1084E+03	1.1033E+03	1.1076E+03
	Mean	1.1076E+03	1.1363E+03	1.1672E+03	1.1983E+03	1.1363E+03	1.1190E+03	1.1283E+03
	Std	2.6459E+00	1.8658E+01	5.0604E+01	7.3858E+01	3.8084E+01	8.2126E+00	1.6923E+01
	Rank	1	4	6	7	5	2	3
F11	Best	2.2731E+03	7.0765E+03	4.3035E+05	5.2080E+05	1.4891E+05	1.0822E+04	3.3161E+04
	Mean	6.0176E+03	1.0415E+06	4.6612E+06	5.2371E+06	2.1520E+06	1.5505E+06	5.5664E+05
	Std	2.4674E+03	1.4556E+06	4.6815E+06	3.3826E+06	2.4412E+06	1.7284E+06	6.0174E+05
	Rank	1	3	6	7	5	4	2
F12	Best	1.3216E+03	1.4699E+03	2.1123E+03	4.0927E+03	2.1731E+03	1.5516E+03	1.7123E+03
	Mean	1.5404E+03	8.8473E+03	3.4039E+04	2.2892E+04	1.0694E+04	1.0359E+04	4.7785E+03
	Std	6.2107E+02	9.1047E+03	2.9406E+04	2.3484E+04	8.4689E+03	8.3378E+03	3.7273E+03
	Rank	1	3	7	6	5	4	2
F13	Best	1.4060E+03	1.4349E+03	1.4437E+03	1.4472E+03	1.4287E+03	1.4355E+03	1.4359E+03
	Mean	1.4197E+03	1.9845E+03	2.0474E+03	2.0490E+03	2.5083E+03	3.2916E+03	1.4588E+03
	Std	7.9296E+00	7.3686E+02	7.3150E+02	7.0483E+02	1.0336E+03	2.4803E+03	1.4634E+01
	Rank	1	3	4	5	6	7	2
F14	Best	1.5024E+03	1.5377E+03	1.6628E+03	1.6407E+03	1.5753E+03	1.5236E+03	1.5454E+03
	Mean	1.5065E+03	2.1469E+03	5.9798E+03	3.4177E+03	3.5726E+03	4.3072E+03	1.6534E+03
	Std	3.7066E+00	9.8283E+02	6.2037E+03	1.6218E+03	2.2672E+03	3.4889E+03	1.0082E+02
	Rank	1	3	7	4	5	6	2
F15	Best	1.6034E+03	1.6029E+03	1.6220E+03	1.6188E+03	1.6246E+03	1.6031E+03	1.6062E+03
	Mean	1.6465E+03	1.6700E+03	1.7257E+03	1.6947E+03	1.7920E+03	1.6747E+03	1.6664E+03
	Std	5.5149E+01	7.0261E+01	1.1038E+02	7.0183E+01	1.0105E+02	8.6550E+01	8.1616E+01
	Rank	1	3	6	5	7	4	2
F16	Best	1.7061E+03	1.7147E+03	1.7331E+03	1.7335E+03	1.7255E+03	1.7106E+03	1.7286E+03
	Mean	1.7348E+03	1.7448E+03	1.7694E+03	1.7732E+03	1.7489E+03	1.7484E+03	1.7504E+03
	Std	1.1868E+01	1.4806E+01	2.7793E+01	2.3828E+01	1.5326E+01	3.2241E+01	1.8149E+01
	Rank	1	2	6	7	4	3	5
F17	Best	1.8130E+03	2.2192E+03	9.9290E+03	7.4769E+03	4.1502E+03	3.2168E+03	2.2587E+03
	Mean	1.8338E+03	2.4056E+04	5.4859E+04	7.9348E+04	2.0018E+04	1.9780E+04	7.4998E+03
	Std	1.3569E+01	1.4407E+04	5.9321E+04	1.1262E+05	1.4007E+04	1.3298E+04	4.5430E+03
	Rank	1	5	6	7	4	3	2
F18	Best	1.9019E+03	1.9144E+03	1.9528E+03	1.9435E+03	1.9302E+03	1.9150E+03	1.9165E+03
	Mean	1.9038E+03	4.2748E+03	8.6585E+03	3.6782E+03	7.8346E+03	5.3413E+03	1.9525E+03
	Std	1.1183E+00	4.6648E+03	7.3340E+03	1.9018E+03	6.2439E+03	4.4800E+03	3.2453E+01
	Rank	1	4	7	3	6	5	2
F19	Best	2.0022E+03	2.0204E+03	2.0404E+03	2.0356E+03	2.0173E+03	2.0085E+03	2.0222E+03
	Mean	2.0252E+03	2.0323E+03	2.0725E+03	2.0650E+03	2.0416E+03	2.0314E+03	2.0476E+03
	Std	6.9262E+00	9.3229E+00	1.9951E+01	1.6765E+01	1.6514E+01	1.1030E+01	1.2731E+01
	Rank	1	3	7	6	4	2	5
F20	Best	2.2001E+03	2.2033E+03	2.2039E+03	2.2065E+03	2.1038E+03	2.2002E+03	2.2037E+03
	Mean	2.2694E+03	2.2622E+03	2.2238E+03	2.2529E+03	2.2401E+03	2.2687E+03	2.2923E+03
	Std	5.1784E+01	5.5331E+01	4.1994E+01	5.8145E+01	6.0624E+01	5.9400E+01	4.8337E+01
	Rank	6	4	1	3	2	5	7
F21	Best	2.3002E+03	2.2293E+03	2.2327E+03	2.2451E+03	2.2472E+03	2.2235E+03	2.2403E+03
	Mean	2.3024E+03	2.3011E+03	2.3046E+03	2.3134E+03	2.3070E+03	2.2972E+03	2.3068E+03
	Std	1.4445E+00	2.5145E+01	3.2063E+01	2.1801E+01	1.4687E+01	2.7946E+01	1.2747E+01
	Rank	3	2	4	7	6	1	5
F22	Best	2.6030E+03	2.6119E+03	2.6192E+03	2.6194E+03	2.6099E+03	2.6109E+03	2.6103E+03
	Mean	2.6109E+03	2.6231E+03	2.6404E+03	2.6328E+03	2.6254E+03	2.6196E+03	2.6197E+03
	Std	5.5501E+00	6.3402E+00	1.1698E+01	7.7455E+00	9.4443E+00	7.1074E+00	5.8055E+00
	Rank	1	4	7	6	5	2	3
F23	Best	2.5014E+03	2.5171E+03	2.5165E+03	2.5342E+03	2.5022E+03	2.5015E+03	2.5089E+03
	Mean	2.7270E+03	2.6939E+03	2.6898E+03	2.7162E+03	2.6942E+03	2.7059E+03	2.7293E+03
	Std	4.2795E+01	9.1954E+01	1.1356E+02	9.1291E+01	1.0202E+02	8.9147E+01	5.9068E+01
	Rank	6	2	1	5	3	4	7
F24	Best	2.8982E+03	2.9022E+03	2.9063E+03	2.9049E+03	2.8982E+03	2.8981E+03	2.8993E+03
	Mean	2.9324E+03	2.9391E+03	2.9518E+03	2.9411E+03	2.9318E+03	2.9267E+03	2.9363E+03
	Std	2.0593E+01	1.7716E+01	2.2104E+01	1.9462E+01	2.7527E+01	2.4221E+01	1.9785E+01
	Rank	3	5	7	6	2	1	4
F25	Best	2.9001E+03	2.9371E+03	2.9285E+03	2.9359E+03	2.6256E+03	2.8208E+03	2.8336E+03
	Mean	2.9004E+03	3.0041E+03	2.9698E+03	2.9847E+03	2.9281E+03	2.9073E+03	2.9505E+03
	Std	8.9534E-01	4.1346E+01	3.3066E+01	2.6742E+01	1.0171E+02	3.1717E+01	4.3012E+01
	Rank	1	7	5	6	3	2	4
F26	Best	3.0900E+03	3.0917E+03	3.0952E+03	3.0921E+03	3.0899E+03	3.0891E+03	3.0897E+03
	Mean	3.0952E+03	3.0961E+03	3.1007E+03	3.0957E+03	3.1051E+03	3.0981E+03	3.0957E+03
	Std	3.0830E+00	2.3771E+00	3.6364E+00	1.9684E+00	2.6113E+01	9.3359E+00	3.2619E+00
	Rank	1	4	6	2	7	5	3
F27	Best	3.1003E+03	3.1455E+03	3.1470E+03	3.1701E+03	3.1712E+03	3.1049E+03	3.1688E+03
	Mean	3.2472E+03	3.2721E+03	3.2289E+03	3.2611E+03	3.2780E+03	3.2578E+03	3.2876E+03
	Std	1.4411E+02	9.8123E+01	6.8487E+01	8.7826E+01	4.4606E+01	1.0908E+02	1.0322E+02
	Rank	2	5	1	4	6	3	7
F28	Best	3.1510E+03	3.1634E+03	3.1641E+03	3.1673E+03	3.1624E+03	3.1458E+03	3.1435E+03
	Mean	3.1781E+03	3.1998E+03	3.2352E+03	3.2320E+03	3.2272E+03	3.2015E+03	3.1998E+03
	Std	1.5368E+01	3.3335E+01	4.8027E+01	4.3537E+01	4.5461E+01	4.2963E+01	3.3793E+01
	Rank	1	3	7	6	5	4	2
F29	Best	3.6850E+03	1.5305E+04	6.2882E+03	1.7845E+04	4.0761E+03	1.0473E+04	6.2787E+03
	Mean	3.7257E+04	7.7955E+05	8.4200E+05	4.5259E+05	4.3102E+05	3.9316E+05	1.7435E+05
	Std	1.7219E+05	7.8556E+05	7.0777E+05	3.6341E+05	6.9757E+05	5.8292E+05	3.9112E+05
	Rank	1	6	7	5	4	3	2

**Table A6:** Comparison of DRIME with other RIME variants in CEC-2017 test suite (D = 30).

No.	Index	DRIME	ACGRIME	TERIME	IRIME	DNMRIME	GLSRIME	HERIME
F1	Best	1.0336E+05	4.8841E+08	1.2960E+09	8.4963E+08	4.8641E+07	1.4735E+07	6.2973E+07
	Mean	5.4087E+05	1.3448E+09	3.3320E+09	1.6033E+09	1.6532E+08	4.6103E+07	1.4147E+08
	Std	4.4719E+05	6.2776E+08	1.0997E+09	4.5194E+08	7.1437E+07	2.4082E+07	5.7468E+07
	Rank	1	5	7	6	4	2	3
F2	Best	5.5212E+02	3.2891E+04	4.0986E+04	5.1987E+04	2.1997E+04	1.4403E+04	2.2396E+04
	Mean	1.4789E+03	4.4268E+04	7.2898E+04	1.0542E+05	3.5318E+04	3.3418E+04	4.0920E+04
	Std	6.3419E+02	6.4916E+03	1.6017E+04	3.4314E+04	6.7233E+03	9.1794E+03	9.8096E+03
	Rank	1	5	6	7	3	2	4
F3	Best	4.9689E+02	5.4655E+02	6.0906E+02	5.5146E+02	5.0778E+02	4.8078E+02	5.0223E+02
	Mean	5.2169E+02	6.6563E+02	7.4032E+02	6.7119E+02	5.5675E+02	5.2737E+02	5.6426E+02
	Std	7.5835E+00	7.3116E+01	1.0862E+02	6.1113E+01	2.9843E+01	2.9919E+01	4.1480E+01
	Rank	1	5	7	6	3	2	4
F4	Best	5.3063E+02	5.9279E+02	6.6056E+02	6.3627E+02	6.0834E+02	5.6999E+02	5.7764E+02
	Mean	5.4376E+02	6.3550E+02	7.1749E+02	6.8435E+02	6.4332E+02	6.0107E+02	6.1189E+02
	Std	1.2058E+01	2.2472E+01	2.9071E+01	2.5816E+01	2.6525E+01	2.2103E+01	2.7785E+01
	Rank	1	4	7	6	5	2	3
F5	Best	6.0037E+02	6.0442E+02	6.1688E+02	6.1467E+02	6.0675E+02	6.0309E+02	6.0510E+02
	Mean	6.0146E+02	6.1319E+02	6.3002E+02	6.2156E+02	6.1966E+02	6.0965E+02	6.0887E+02
	Std	7.5368E-01	5.1186E+00	7.4612E+00	4.1436E+00	6.6782E+00	4.5907E+00	3.1014E+00
	Rank	1	4	7	6	5	3	2
F6	Best	7.6244E+02	8.3319E+02	1.0073E+03	9.4120E+02	8.7771E+02	8.4552E+02	8.3710E+02
	Mean	8.0749E+02	8.9098E+02	1.1095E+03	9.9628E+02	9.3706E+02	9.0891E+02	9.0316E+02
	Std	3.0163E+01	2.8834E+01	6.6344E+01	3.4708E+01	3.0782E+01	2.8721E+01	2.8076E+01
	Rank	1	2	7	6	5	4	3
F7	Best	8.1895E+02	8.9347E+02	9.6151E+02	9.2753E+02	8.9590E+02	8.6156E+02	8.6186E+02
	Mean	8.3161E+02	9.2489E+02	1.0258E+03	9.7279E+02	9.4118E+02	9.1644E+02	8.9859E+02
	Std	7.4953E+00	1.7143E+01	3.3795E+01	2.5930E+01	2.2525E+01	2.9832E+01	2.0905E+01
	Rank	1	4	7	6	5	3	2
F8	Best	9.0167E+02	1.5021E+03	2.3938E+03	2.0870E+03	2.0969E+03	1.0240E+03	9.7810E+02
	Mean	9.1526E+02	2.3034E+03	5.2723E+03	3.3540E+03	4.2497E+03	2.1969E+03	1.2764E+03
	Std	1.0496E+01	5.1485E+02	2.1518E+03	8.3031E+02	1.8090E+03	1.4347E+03	1.9355E+02
	Rank	1	4	7	5	6	3	2
F9	Best	3.0332E+03	4.3911E+03	4.9054E+03	5.0989E+03	4.2081E+03	3.3031E+03	4.1513E+03
	Mean	4.4306E+03	5.3795E+03	6.7718E+03	6.7347E+03	5.6576E+03	4.9137E+03	5.4832E+03
	Std	6.4303E+02	5.3014E+02	6.9386E+02	6.2874E+02	5.9367E+02	7.8958E+02	6.4670E+02
	Rank	1	3	7	6	5	2	4
F10	Best	1.1616E+03	1.3201E+03	1.3841E+03	1.5170E+03	1.2287E+03	1.2068E+03	1.2766E+03
	Mean	1.2087E+03	1.5285E+03	1.5537E+03	1.8199E+03	1.3511E+03	1.3553E+03	1.3577E+03
	Std	2.3476E+01	1.5127E+02	1.7449E+02	2.5976E+02	5.9422E+01	6.8214E+01	5.0528E+01
	Rank	1	5	6	7	2	3	4
F11	Best	4.1685E+05	4.7962E+06	4.4022E+07	1.3099E+07	4.0568E+06	2.8368E+06	2.4011E+06
	Mean	1.4532E+06	3.2360E+07	1.3008E+08	6.0499E+07	2.9955E+07	2.9073E+07	1.9569E+07
	Std	6.1274E+05	2.6793E+07	5.9344E+07	3.5924E+07	1.9657E+07	3.9080E+07	1.7633E+07
	Rank	1	5	7	6	4	3	2
F12	Best	6.6651E+03	3.0771E+04	2.0116E+06	7.7864E+05	1.9981E+05	8.8731E+04	1.5059E+05
	Mean	1.7523E+04	3.1011E+05	2.7515E+07	6.0575E+06	8.3208E+05	3.3007E+05	4.8519E+05
	Std	6.3923E+03	6.1089E+05	2.2988E+07	6.7088E+06	8.1931E+05	1.7692E+05	3.7970E+05
	Rank	1	2	7	6	5	3	4
F13	Best	1.4710E+03	1.3800E+04	3.2359E+04	1.0429E+04	6.8502E+03	5.5607E+03	3.2881E+03
	Mean	1.5007E+03	1.6460E+05	2.0947E+05	1.5245E+05	1.0551E+05	5.8419E+04	9.4105E+03
	Std	1.6259E+01	2.4078E+05	2.0618E+05	1.1400E+05	7.9645E+04	3.6039E+04	6.8349E+03
	Rank	1	6	7	5	4	3	2
F14	Best	1.7322E+03	6.1418E+03	2.0093E+05	5.0626E+04	1.5981E+04	2.4272E+04	1.1373E+04
	Mean	2.2916E+03	1.9363E+04	2.7200E+06	1.5671E+05	1.1890E+05	6.6010E+04	5.3294E+04
	Std	4.1223E+02	1.0715E+04	3.7554E+06	1.0075E+05	9.5072E+04	3.4445E+04	3.2617E+04
	Rank	1	2	7	6	5	4	3
F15	Best	1.9480E+03	2.0443E+03	2.5661E+03	2.3203E+03	2.2850E+03	1.8566E+03	2.1492E+03
	Mean	2.4857E+03	2.6595E+03	3.0565E+03	2.9367E+03	2.7732E+03	2.6203E+03	2.6483E+03
	Std	3.0596E+02	3.0151E+02	3.1510E+02	2.7176E+02	2.9717E+02	2.8277E+02	2.9941E+02
	Rank	1	4	7	6	5	2	3
F16	Best	1.7644E+03	1.8114E+03	1.8990E+03	1.8465E+03	1.8406E+03	1.8533E+03	1.8289E+03
	Mean	1.8781E+03	2.0837E+03	2.2319E+03	2.2215E+03	2.1648E+03	2.1095E+03	2.0881E+03
	Std	8.9640E+01	1.6642E+02	1.9376E+02	1.9749E+02	1.7615E+02	1.6759E+02	1.7869E+02
	Rank	1	2	7	6	5	4	3
F17	Best	2.0838E+03	1.5737E+05	3.2312E+05	3.2971E+05	1.8164E+05	9.8760E+04	4.6539E+04
	Mean	4.0202E+03	1.3578E+06	3.7876E+06	3.3920E+06	1.5970E+06	1.1443E+06	2.2500E+05
	Std	2.0100E+03	1.6140E+06	3.1266E+06	3.6537E+06	4.4564E+06	1.2295E+06	1.3385E+05
	Rank	1	4	7	6	5	3	2
F18	Best	2.0235E+03	6.3676E+03	2.8112E+05	7.0371E+04	6.2003E+04	1.2922E+04	6.4667E+03
	Mean	3.3183E+03	1.4031E+05	2.4671E+06	5.6837E+05	7.1626E+05	3.8888E+05	1.0203E+05
	Std	1.4974E+03	2.5767E+05	1.8858E+06	5.0652E+05	5.3642E+05	3.7354E+05	9.7780E+04
	Rank	1	3	7	5	6	4	2
F19	Best	2.1136E+03	2.2249E+03	2.1895E+03	2.2448E+03	2.2011E+03	2.1544E+03	2.1143E+03
	Mean	2.2479E+03	2.4231E+03	2.4515E+03	2.4874E+03	2.4735E+03	2.4410E+03	2.4599E+03
	Std	5.0813E+01	1.5198E+02	1.5118E+02	1.4624E+02	1.4685E+02	1.5792E+02	1.4567E+02
	Rank	1	2	4	7	6	3	5
F20	Best	2.3195E+03	2.3806E+03	2.4632E+03	2.4264E+03	2.3757E+03	2.3652E+03	2.3710E+03
	Mean	2.3386E+03	2.4195E+03	2.5173E+03	2.4670E+03	2.4220E+03	2.4047E+03	2.4031E+03
	Std	1.9274E+01	2.0210E+01	2.8866E+01	1.8847E+01	2.4847E+01	1.9789E+01	1.8972E+01
	Rank	1	4	7	6	5	3	2
F21	Best	2.3025E+03	2.4430E+03	2.6129E+03	2.5170E+03	2.3326E+03	2.3217E+03	2.3515E+03
	Mean	2.3085E+03	2.5653E+03	5.1810E+03	4.4405E+03	2.3712E+03	3.7244E+03	2.6902E+03
	Std	3.2937E+00	8.1662E+01	2.8219E+03	2.6040E+03	2.2478E+01	1.8850E+03	1.1834E+03
	Rank	1	3	7	6	2	5	4
F22	Best	2.6729E+03	2.7334E+03	2.8174E+03	2.7859E+03	2.7297E+03	2.7223E+03	2.7126E+03
	Mean	2.6954E+03	2.7740E+03	2.8806E+03	2.8255E+03	2.7818E+03	2.7741E+03	2.7627E+03
	Std	9.5088E+00	1.9033E+01	3.4206E+01	1.8417E+01	2.9431E+01	2.6143E+01	2.4350E+01
	Rank	1	3	7	6	5	4	2
F23	Best	2.8353E+03	2.9043E+03	3.0279E+03	2.9300E+03	2.9014E+03	2.8745E+03	2.8876E+03
	Mean	2.8578E+03	2.9482E+03	3.0758E+03	3.0064E+03	2.9474E+03	2.9135E+03	2.9354E+03
	Std	1.3832E+01	2.5985E+01	3.1344E+01	3.1738E+01	2.8896E+01	1.9960E+01	2.1181E+01
	Rank	1	5	7	6	4	2	3
F24	Best	2.9025E+03	2.9808E+03	2.9784E+03	2.9686E+03	2.9158E+03	2.9039E+03	2.9013E+03
	Mean	2.9293E+03	3.0490E+03	3.1056E+03	3.0390E+03	2.9718E+03	2.9425E+03	2.9409E+03
	Std	9.6698E+00	3.8988E+01	7.1104E+01	4.4591E+01	2.5050E+01	3.0919E+01	2.3965E+01
	Rank	1	6	7	5	4	3	2
F25	Best	2.8244E+03	3.7719E+03	4.3158E+03	3.9297E+03	3.0812E+03	4.4363E+03	3.5239E+03
	Mean	4.1381E+03	4.8631E+03	6.0762E+03	5.6024E+03	4.5786E+03	4.8746E+03	4.7859E+03
	Std	3.8884E+02	6.0370E+02	4.8961E+02	4.0049E+02	1.0200E+03	3.6468E+02	3.2753E+02
	Rank	1	4	7	6	2	5	3
F26	Best	3.2275E+03	3.2163E+03	3.2382E+03	3.2143E+03	3.2000E+03	3.2106E+03	3.2136E+03
	Mean	3.2394E+03	3.2508E+03	3.2814E+03	3.2477E+03	3.2000E+03	3.2383E+03	3.2436E+03
	Std	7.6155E+00	2.2702E+01	1.9138E+01	1.4444E+01	7.6087E-05	1.3731E+01	1.5140E+01
	Rank	3	6	7	5	1	2	4
F27	Best	3.2372E+03	3.3275E+03	3.3612E+03	3.3230E+03	3.2960E+03	3.2615E+03	3.2601E+03
	Mean	3.2901E+03	3.4473E+03	3.4709E+03	3.4557E+03	3.3201E+03	3.3143E+03	3.3309E+03
	Std	1.3314E+01	6.6006E+01	7.3040E+01	7.9150E+01	2.5143E+01	4.9278E+01	3.2898E+01
	Rank	1	5	7	6	3	2	4
F28	Best	3.4884E+03	3.5795E+03	3.5442E+03	3.6876E+03	3.5085E+03	3.5564E+03	3.5498E+03
	Mean	3.5727E+03	3.8971E+03	4.0219E+03	3.9822E+03	3.8964E+03	3.9120E+03	3.8454E+03
	Std	7.9040E+01	1.9970E+02	2.1906E+02	1.6226E+02	1.7720E+02	1.7851E+02	1.7905E+02
	Rank	1	4	7	6	3	5	2
F29	Best	1.4092E+04	3.1747E+05	5.8935E+05	2.9409E+05	2.0665E+05	5.0334E+05	3.7614E+05
	Mean	2.3200E+04	2.9804E+06	3.7727E+06	1.8312E+06	4.3743E+06	2.2049E+06	1.3340E+06
	Std	9.4801E+03	2.6411E+06	3.3171E+06	1.2379E+06	2.4402E+06	1.7938E+06	9.3962E+05
	Rank	1	5	6	3	7	4	2

**Table A7:** Comparison of DRIME with other RIME variants in CEC-2017 test suite (D = 50).

No.	Index	DRIME	ACGRIME	TERIME	IRIME	DNMRIME	GLSRIME	HERIME
F1	Best	4.0061E+06	4.3160E+09	7.4463E+09	5.0200E+09	4.8836E+08	1.1144E+08	4.7760E+08
	Mean	2.0985E+07	9.6500E+09	1.4032E+10	7.4145E+09	9.3538E+08	2.1448E+08	8.9173E+08
	Std	1.2195E+07	2.8044E+09	4.4315E+09	1.6001E+09	2.5324E+08	7.7010E+07	2.5048E+08
	Rank	1	6	7	5	4	2	3
F2	Best	6.4166E+03	9.6258E+04	1.1077E+05	1.6648E+05	6.6120E+04	8.1551E+04	9.4597E+04
	Mean	1.1025E+04	1.2230E+05	1.9550E+05	2.3373E+05	1.0506E+05	1.0801E+05	1.2541E+05
	Std	2.7665E+03	1.5333E+04	3.5815E+04	3.9088E+04	1.8836E+04	1.9201E+04	1.5473E+04
	Rank	1	4	6	7	2	3	5
F3	Best	6.1259E+02	9.5752E+02	1.1993E+03	9.4378E+02	6.5411E+02	5.9392E+02	6.6341E+02
	Mean	6.6641E+02	1.4279E+03	1.6360E+03	1.2605E+03	8.2876E+02	7.0132E+02	7.6319E+02
	Std	2.3270E+01	2.7922E+02	2.6796E+02	1.6559E+02	8.2911E+01	6.7552E+01	6.4043E+01
	Rank	1	6	7	5	4	2	3
F4	Best	5.6453E+02	7.3863E+02	8.7670E+02	7.6958E+02	7.5898E+02	6.5922E+02	6.7235E+02
	Mean	5.9621E+02	8.2722E+02	9.5194E+02	8.6983E+02	8.5904E+02	7.1999E+02	7.3665E+02
	Std	1.6224E+01	4.7839E+01	4.7833E+01	4.1180E+01	5.0941E+01	4.1845E+01	3.7656E+01
	Rank	1	4	7	6	5	2	3
F5	Best	6.0127E+02	6.1454E+02	6.3323E+02	6.2104E+02	6.2001E+02	6.0974E+02	6.0965E+02
	Mean	6.0404E+02	6.2463E+02	6.4524E+02	6.3015E+02	6.3694E+02	6.1936E+02	6.1665E+02
	Std	1.3802E+00	5.7147E+00	7.1778E+00	4.8244E+00	9.1159E+00	4.7932E+00	3.4328E+00
	Rank	1	4	7	5	6	3	2
F6	Best	8.3515E+02	1.0492E+03	1.3950E+03	1.1734E+03	1.1204E+03	1.0488E+03	9.8411E+02
	Mean	9.2051E+02	1.1531E+03	1.6014E+03	1.2702E+03	1.2355E+03	1.1590E+03	1.1112E+03
	Std	4.2875E+01	6.0461E+01	1.0043E+02	4.5683E+01	6.1200E+01	6.6925E+01	5.5200E+01
	Rank	1	3	7	6	5	4	2
F7	Best	8.6561E+02	1.0636E+03	1.1452E+03	1.0909E+03	1.0540E+03	9.2434E+02	9.7990E+02
	Mean	8.9997E+02	1.1281E+03	1.2406E+03	1.1541E+03	1.1265E+03	1.0352E+03	1.0330E+03
	Std	2.5593E+01	3.8945E+01	4.5361E+01	3.8378E+01	4.1006E+01	4.4583E+01	2.8093E+01
	Rank	1	5	7	6	4	3	2
F8	Best	1.0340E+03	5.4360E+03	8.0719E+03	8.1368E+03	3.4808E+03	2.9768E+03	2.1596E+03
	Mean	1.2891E+03	9.7377E+03	1.5865E+04	1.1704E+04	1.4169E+04	7.0123E+03	3.3747E+03
	Std	2.0886E+02	3.1598E+03	4.9580E+03	2.8518E+03	6.4904E+03	3.6817E+03	1.0067E+03
	Rank	1	4	7	5	6	3	2
F9	Best	6.0615E+03	7.8792E+03	1.0238E+04	9.9878E+03	7.3709E+03	6.6421E+03	7.9677E+03
	Mean	7.5995E+03	9.6049E+03	1.1354E+04	1.1399E+04	9.5865E+03	8.5313E+03	9.5969E+03
	Std	9.6850E+02	7.1470E+02	6.8686E+02	8.0164E+02	1.2410E+03	9.6025E+02	1.0518E+03
	Rank	1	5	6	7	3	2	4
F10	Best	1.2720E+03	1.9162E+03	1.7759E+03	3.0395E+03	1.6190E+03	1.4811E+03	1.5161E+03
	Mean	1.3273E+03	2.8564E+03	2.9761E+03	4.4648E+03	1.9868E+03	1.7601E+03	1.8652E+03
	Std	4.0439E+01	5.7464E+02	6.5546E+02	1.0974E+03	1.7930E+02	1.3505E+02	1.7615E+02
	Rank	1	5	6	7	4	2	3
F11	Best	3.9022E+06	1.3678E+08	6.1262E+08	3.3268E+08	6.0217E+07	1.7972E+07	6.1562E+07
	Mean	1.7893E+07	3.9805E+08	1.3215E+09	5.1778E+08	2.9731E+08	1.6899E+08	2.1223E+08
	Std	9.2710E+06	2.0055E+08	5.5261E+08	1.4919E+08	1.8002E+08	9.0723E+07	1.3960E+08
	Rank	1	5	7	6	4	2	3
F12	Best	1.5808E+04	2.2377E+05	2.0397E+07	1.6026E+07	1.2121E+06	4.9978E+05	4.2918E+05
	Mean	2.0819E+04	8.8138E+06	1.1565E+08	3.8972E+07	6.6019E+06	1.4702E+06	2.6279E+06
	Std	3.6439E+03	1.8336E+07	7.4485E+07	1.9922E+07	5.6882E+06	7.0791E+05	2.2628E+06
	Rank	1	5	7	6	4	2	3
F13	Best	1.5816E+03	1.6835E+05	3.2142E+05	1.1499E+05	9.7100E+04	7.5670E+04	2.1875E+04
	Mean	1.6520E+03	1.2608E+06	1.7423E+06	7.7348E+05	6.3666E+05	3.9081E+05	1.1939E+05
	Std	4.2203E+01	9.7651E+05	1.0786E+06	4.8758E+05	4.3643E+05	2.6936E+05	5.5707E+04
	Rank	1	6	7	5	4	3	2
F14	Best	3.7229E+03	1.6900E+04	3.4446E+06	3.0362E+05	1.0442E+05	3.7243E+04	6.1344E+04
	Mean	6.9619E+03	1.3479E+05	2.1192E+07	2.5133E+06	9.1928E+05	2.4854E+05	2.7178E+05
	Std	1.6229E+03	2.6137E+05	1.6330E+07	1.8905E+06	1.0788E+06	1.6771E+05	2.1330E+05
	Rank	1	2	7	6	5	3	4
F15	Best	2.1112E+03	2.7311E+03	3.6773E+03	2.6840E+03	2.7565E+03	2.8784E+03	2.4884E+03
	Mean	2.5272E+03	3.3281E+03	4.5629E+03	3.9563E+03	3.6803E+03	3.5003E+03	3.3935E+03
	Std	2.6810E+02	3.6666E+02	4.0005E+02	3.9452E+02	3.6800E+02	3.3075E+02	4.5568E+02
	Rank	1	2	7	6	5	4	3
F16	Best	2.2575E+03	2.5518E+03	2.4124E+03	2.6005E+03	2.7540E+03	2.5052E+03	2.4218E+03
	Mean	2.7246E+03	3.0939E+03	3.5473E+03	3.3702E+03	3.2013E+03	3.1138E+03	3.0158E+03
	Std	2.2001E+02	2.8413E+02	4.1792E+02	3.0022E+02	2.3169E+02	3.2528E+02	2.9847E+02
	Rank	1	3	7	6	5	4	2
F17	Best	3.6396E+03	8.1870E+05	1.2530E+06	8.0491E+05	7.0788E+05	7.4940E+05	2.8211E+05
	Mean	8.3535E+03	4.1321E+06	1.0436E+07	5.7925E+06	3.8129E+06	3.8833E+06	1.4088E+06
	Std	4.5065E+03	3.2992E+06	7.6470E+06	4.7025E+06	2.0800E+06	2.6030E+06	1.0344E+06
	Rank	1	5	7	6	3	4	2
F18	Best	1.0285E+04	5.7372E+04	1.6781E+06	2.0122E+05	1.0604E+05	1.2285E+05	8.5345E+04
	Mean	1.6155E+04	7.1641E+05	1.2194E+07	1.5156E+06	1.0430E+06	1.6525E+06	4.4752E+05
	Std	2.9681E+03	8.7414E+05	9.7777E+06	8.8988E+05	1.2848E+06	1.4633E+06	3.2383E+05
	Rank	1	3	7	5	4	6	2
F19	Best	2.2472E+03	2.3842E+03	2.7148E+03	2.5950E+03	2.5504E+03	2.6511E+03	2.3827E+03
	Mean	2.6364E+03	3.0737E+03	3.4159E+03	3.2706E+03	3.1597E+03	3.0874E+03	3.0737E+03
	Std	2.3806E+02	2.6561E+02	2.9494E+02	2.8950E+02	2.6206E+02	2.4262E+02	3.3943E+02
	Rank	1	2	7	6	5	4	3
F20	Best	2.3532E+03	2.4795E+03	2.6692E+03	2.5989E+03	2.4901E+03	2.4426E+03	2.4735E+03
	Mean	2.3836E+03	2.5607E+03	2.7396E+03	2.6726E+03	2.5666E+03	2.5339E+03	2.5362E+03
	Std	2.0439E+01	4.0378E+01	4.0096E+01	3.6702E+01	4.0530E+01	6.1555E+01	3.8940E+01
	Rank	1	4	7	6	5	2	3
F21	Best	2.3353E+03	4.1046E+03	1.1452E+04	1.2159E+04	2.5727E+03	7.5733E+03	8.8890E+03
	Mean	8.2025E+03	1.1186E+04	1.3390E+04	1.3182E+04	1.1167E+04	1.0037E+04	1.1463E+04
	Std	3.2379E+03	2.0236E+03	9.2853E+02	6.0341E+02	3.3163E+03	1.0058E+03	1.0215E+03
	Rank	1	4	7	6	3	2	5
F22	Best	2.7913E+03	2.9374E+03	3.1276E+03	3.0226E+03	2.9164E+03	2.8762E+03	2.9014E+03
	Mean	2.8347E+03	3.0275E+03	3.2210E+03	3.1192E+03	3.0473E+03	2.9780E+03	2.9947E+03
	Std	2.3233E+01	4.2403E+01	5.8147E+01	4.3726E+01	5.6929E+01	4.8666E+01	4.3853E+01
	Rank	1	4	7	6	5	2	3
F23	Best	2.9502E+03	3.0795E+03	3.3218E+03	3.2209E+03	3.0803E+03	3.0266E+03	3.0659E+03
	Mean	2.9809E+03	3.1871E+03	3.4722E+03	3.2751E+03	3.1984E+03	3.1350E+03	3.1438E+03
	Std	1.7682E+01	4.3817E+01	6.5021E+01	2.9485E+01	7.6943E+01	4.7174E+01	4.2948E+01
	Rank	1	4	7	6	5	2	3
F24	Best	3.1392E+03	3.3488E+03	3.4123E+03	3.3512E+03	3.1686E+03	3.0908E+03	3.1687E+03
	Mean	3.1865E+03	4.0210E+03	4.0792E+03	3.7676E+03	3.3245E+03	3.1796E+03	3.2615E+03
	Std	2.8716E+01	3.0357E+02	3.8422E+02	2.2412E+02	8.5180E+01	5.4280E+01	6.4202E+01
	Rank	2	6	7	5	4	1	3
F25	Best	4.8458E+03	5.2553E+03	8.0799E+03	4.9044E+03	3.5944E+03	5.5947E+03	5.6521E+03
	Mean	5.2630E+03	6.4561E+03	8.9675E+03	7.4811E+03	5.0324E+03	6.3443E+03	6.4500E+03
	Std	1.9879E+02	9.6001E+02	5.2806E+02	7.2226E+02	1.6906E+03	5.4285E+02	3.9498E+02
	Rank	2	5	7	6	1	3	4
F26	Best	3.3985E+03	3.4150E+03	3.5303E+03	3.4280E+03	3.2000E+03	3.3848E+03	3.3742E+03
	Mean	3.4901E+03	3.6151E+03	3.7244E+03	3.5845E+03	3.3695E+03	3.5157E+03	3.5173E+03
	Std	4.4434E+01	9.1322E+01	8.9351E+01	9.9326E+01	1.9144E+02	8.3029E+01	6.8193E+01
	Rank	2	6	7	5	1	3	4
F27	Best	3.4371E+03	3.9395E+03	4.0177E+03	3.6898E+03	3.3000E+03	3.3475E+03	3.4815E+03
	Mean	3.5491E+03	4.6911E+03	4.6910E+03	4.3581E+03	3.7339E+03	3.5405E+03	3.6529E+03
	Std	5.0592E+01	3.5288E+02	6.3082E+02	4.9196E+02	1.9468E+02	1.1703E+02	1.2466E+02
	Rank	2	7	6	5	4	1	3
F28	Best	3.8499E+03	3.9833E+03	4.2297E+03	4.1955E+03	4.1836E+03	4.0593E+03	4.1059E+03
	Mean	4.1930E+03	4.8126E+03	4.9729E+03	4.8320E+03	5.0491E+03	4.7147E+03	4.6534E+03
	Std	1.5125E+02	4.2405E+02	3.8542E+02	3.4029E+02	4.2764E+02	2.9177E+02	2.8700E+02
	Rank	1	4	6	5	7	3	2
F29	Best	1.1161E+07	3.9769E+07	2.4891E+07	1.8713E+07	3.2753E+07	2.7108E+07	2.3689E+07
	Mean	2.1554E+07	8.3325E+07	5.5545E+07	4.2073E+07	9.9016E+07	6.1804E+07	4.6483E+07
	Std	6.1361E+06	2.4541E+07	2.8287E+07	1.4859E+07	3.4262E+07	2.2536E+07	1.5251E+07
	Rank	1	6	4	2	7	5	3

**Table A8:** Comparison of DRIME with other RIME variants in CEC-2017 test suite (D = 100).

No.	Index	DRIME	ACGRIME	TERIME	IRIME	DNMRIME	GLSRIME	HERIME
F1	Best	1.8597E+08	3.9328E+10	6.4886E+10	2.9514E+10	5.0905E+09	1.3819E+09	3.9484E+09
	Mean	4.5502E+08	5.0241E+10	8.1611E+10	3.9491E+10	8.0830E+09	2.6819E+09	6.1836E+09
	Std	2.1106E+08	6.6045E+09	1.2335E+10	6.5991E+09	1.8400E+09	6.5276E+08	1.2475E+09
	Rank	1	6	7	5	4	2	3
F2	Best	5.4624E+04	2.4434E+05	3.9951E+05	3.8923E+05	2.2300E+05	2.6540E+05	3.2376E+05
	Mean	7.0773E+04	2.8223E+05	5.2072E+05	5.7180E+05	2.6333E+05	3.9684E+05	3.7742E+05
	Std	9.0094E+03	1.5469E+04	7.1239E+04	1.0355E+05	1.6610E+04	5.5156E+04	3.2786E+04
	Rank	1	3	6	7	2	5	4
F3	Best	9.2033E+02	3.3326E+03	4.2514E+03	3.2770E+03	1.3181E+03	1.0685E+03	1.2032E+03
	Mean	1.0414E+03	5.5311E+03	7.3815E+03	4.8939E+03	1.8251E+03	1.2902E+03	1.5948E+03
	Std	6.8193E+01	1.2841E+03	2.0117E+03	1.0735E+03	2.7954E+02	1.5098E+02	2.4419E+02
	Rank	1	6	7	5	4	2	3
F4	Best	7.6337E+02	1.2541E+03	1.5347E+03	1.3782E+03	1.1830E+03	1.1217E+03	1.0682E+03
	Mean	8.3476E+02	1.4486E+03	1.6720E+03	1.5119E+03	1.4967E+03	1.2467E+03	1.1685E+03
	Std	5.5361E+01	9.4593E+01	8.3029E+01	6.9030E+01	1.3937E+02	6.9196E+01	4.9622E+01
	Rank	1	4	7	6	5	3	2
F5	Best	6.0875E+02	6.3135E+02	6.4871E+02	6.4413E+02	6.4487E+02	6.3577E+02	6.2449E+02
	Mean	6.1504E+02	6.4396E+02	6.6914E+02	6.5521E+02	6.6088E+02	6.5070E+02	6.3294E+02
	Std	2.8775E+00	5.4798E+00	8.3877E+00	4.5340E+00	9.0183E+00	1.0144E+01	5.3088E+00
	Rank	1	3	7	5	6	4	2
F6	Best	1.2401E+03	1.8517E+03	3.1998E+03	2.1830E+03	1.8387E+03	1.8575E+03	1.7025E+03
	Mean	1.3717E+03	2.1400E+03	3.6928E+03	2.3523E+03	2.0790E+03	2.0783E+03	1.9046E+03
	Std	8.2306E+01	1.2327E+02	2.9908E+02	9.8547E+01	1.4747E+02	1.0387E+02	8.0924E+01
	Rank	1	5	7	6	4	3	2
F7	Best	1.0340E+03	1.5629E+03	1.8626E+03	1.6912E+03	1.5117E+03	1.3382E+03	1.3299E+03
	Mean	1.1217E+03	1.6586E+03	2.0173E+03	1.8296E+03	1.7272E+03	1.5378E+03	1.4510E+03
	Std	4.1154E+01	4.6134E+01	9.1561E+01	8.6443E+01	1.2716E+02	1.0532E+02	6.5784E+01
	Rank	1	4	7	6	5	3	2
F8	Best	4.3350E+03	2.2956E+04	4.2520E+04	3.0321E+04	3.5883E+04	2.4220E+04	9.1247E+03
	Mean	6.6472E+03	3.7888E+04	6.2629E+04	4.5665E+04	5.0205E+04	3.8782E+04	1.5460E+04
	Std	1.1423E+03	6.0589E+03	1.4153E+04	7.0134E+03	6.9621E+03	9.6107E+03	3.3082E+03
	Rank	1	3	7	5	6	4	2
F9	Best	1.4964E+04	1.8336E+04	2.3400E+04	2.3718E+04	1.8916E+04	1.7115E+04	2.0189E+04
	Mean	1.8742E+04	2.1915E+04	2.6207E+04	2.7134E+04	2.1932E+04	1.9877E+04	2.2493E+04
	Std	2.5902E+03	1.6053E+03	1.5107E+03	1.5093E+03	2.3557E+03	1.4441E+03	1.3086E+03
	Rank	1	3	6	7	4	2	5
F10	Best	2.6623E+03	2.9090E+04	3.4028E+04	8.7468E+04	1.4369E+04	9.2486E+03	1.2090E+04
	Mean	3.1655E+03	4.8985E+04	5.7630E+04	1.2768E+05	2.5968E+04	1.6822E+04	2.2318E+04
	Std	2.9751E+02	1.1152E+04	1.5455E+04	2.5904E+04	5.9185E+03	3.9107E+03	5.2959E+03
	Rank	1	5	6	7	4	2	3
F11	Best	3.3608E+08	1.8996E+09	6.0531E+09	2.7739E+09	7.5838E+08	3.4544E+08	3.7702E+08
	Mean	4.3000E+08	4.8801E+09	1.1714E+10	4.4593E+09	1.4476E+09	1.2164E+09	1.1711E+09
	Std	4.9462E+07	1.6328E+09	3.2462E+09	1.0683E+09	4.5234E+08	6.6048E+08	4.5570E+08
	Rank	1	6	7	5	4	3	2
F12	Best	1.8665E+04	1.0586E+07	2.6106E+08	8.4496E+07	5.9183E+06	2.6084E+06	3.8870E+06
	Mean	2.3730E+04	1.0022E+08	5.6490E+08	1.4527E+08	1.6880E+07	6.4402E+06	1.1457E+07
	Std	3.1901E+03	8.0293E+07	2.6528E+08	4.6143E+07	6.7492E+06	2.3102E+06	5.1872E+06
	Rank	1	5	7	6	4	2	3
F13	Best	2.0737E+03	3.9317E+06	3.8999E+06	2.3711E+06	1.4175E+06	1.3645E+06	9.2361E+05
	Mean	3.6614E+03	6.6797E+06	1.4047E+07	7.5816E+06	3.9435E+06	5.9876E+06	2.3067E+06
	Std	2.4276E+03	2.0600E+06	6.4837E+06	2.6250E+06	1.4042E+06	3.5157E+06	8.9681E+05
	Rank	1	5	7	6	3	4	2
F14	Best	1.0036E+04	4.2785E+04	2.5759E+07	5.2135E+06	8.2252E+05	3.9109E+05	2.8735E+05
	Mean	1.2583E+04	1.3612E+06	8.4521E+07	2.2520E+07	2.8842E+06	1.6707E+06	2.0102E+06
	Std	1.3319E+03	2.2465E+06	5.8730E+07	1.0246E+07	1.4897E+06	2.5877E+06	4.2532E+06
	Rank	1	2	7	6	5	3	4
F15	Best	3.3743E+03	5.5161E+03	7.8063E+03	7.3628E+03	5.2052E+03	5.2825E+03	5.2019E+03
	Mean	5.1112E+03	6.4767E+03	9.6269E+03	8.2317E+03	6.7504E+03	6.8295E+03	6.4934E+03
	Std	6.0194E+02	6.7387E+02	8.5225E+02	4.6052E+02	7.5944E+02	7.6965E+02	6.6698E+02
	Rank	1	2	7	6	4	5	3
F16	Best	2.8039E+03	4.0408E+03	5.8967E+03	4.8243E+03	4.3033E+03	4.3542E+03	4.3623E+03
	Mean	4.0253E+03	5.4390E+03	6.7896E+03	6.0732E+03	5.3867E+03	5.4130E+03	5.4031E+03
	Std	4.0261E+02	6.4325E+02	5.2678E+02	5.7452E+02	5.6059E+02	5.3053E+02	6.1661E+02
	Rank	1	5	7	6	2	4	3
F17	Best	1.7350E+04	2.0880E+06	5.6762E+06	2.2980E+06	1.2931E+06	1.0417E+06	1.3971E+06
	Mean	3.6221E+04	6.5180E+06	2.4884E+07	9.0866E+06	4.2033E+06	8.5962E+06	3.7657E+06
	Std	1.3042E+04	3.0273E+06	1.2162E+07	4.4881E+06	1.7955E+06	6.1433E+06	1.4362E+06
	Rank	1	4	7	6	3	5	2
F18	Best	4.1133E+04	8.7197E+05	2.4860E+07	1.1794E+07	1.3664E+06	4.5249E+06	1.7425E+06
	Mean	1.3806E+05	8.1380E+06	9.0344E+07	3.6250E+07	1.3147E+07	1.8419E+07	7.1568E+06
	Std	6.3792E+04	6.4734E+06	6.1796E+07	1.3974E+07	1.0640E+07	1.0468E+07	4.5118E+06
	Rank	1	3	7	6	4	5	2
F19	Best	3.1375E+03	4.1496E+03	5.0702E+03	4.9590E+03	4.6511E+03	3.9881E+03	3.8986E+03
	Mean	4.3069E+03	5.1494E+03	6.5159E+03	6.2055E+03	5.5565E+03	5.2381E+03	5.2328E+03
	Std	5.0682E+02	5.1948E+02	5.0371E+02	5.4310E+02	6.2758E+02	5.4873E+02	4.9672E+02
	Rank	1	2	7	6	5	4	3
F20	Best	2.5424E+03	2.9650E+03	3.2625E+03	3.1773E+03	2.9197E+03	2.8910E+03	2.8675E+03
	Mean	2.5954E+03	3.0930E+03	3.5492E+03	3.3496E+03	3.1273E+03	3.1137E+03	2.9999E+03
	Std	3.5985E+01	7.8918E+01	1.3029E+02	7.4472E+01	1.0236E+02	1.1516E+02	6.4532E+01
	Rank	1	3	7	6	5	4	2
F21	Best	1.7569E+04	1.3940E+04	2.6139E+04	2.6893E+04	3.5630E+03	1.9394E+04	2.1437E+04
	Mean	2.0584E+04	2.4752E+04	2.8916E+04	2.9650E+04	2.3792E+04	2.2116E+04	2.5250E+04
	Std	2.2566E+03	2.3637E+03	1.4059E+03	1.2664E+03	5.5420E+03	1.3158E+03	1.8875E+03
	Rank	1	4	6	7	3	2	5
F22	Best	3.0879E+03	3.4415E+03	3.8255E+03	3.6354E+03	3.4704E+03	3.3584E+03	3.3638E+03
	Mean	3.1578E+03	3.5512E+03	4.0575E+03	3.7764E+03	3.6559E+03	3.5266E+03	3.5104E+03
	Std	4.3185E+01	5.4284E+01	1.1057E+02	7.7362E+01	9.4871E+01	9.5931E+01	7.2241E+01
	Rank	1	4	7	6	5	3	2
F23	Best	3.5022E+03	3.9897E+03	4.5437E+03	4.1909E+03	3.9805E+03	3.9065E+03	3.9146E+03
	Mean	3.5991E+03	4.1538E+03	4.7647E+03	4.3827E+03	5.0629E+03	4.0611E+03	4.0821E+03
	Std	4.3531E+01	8.5632E+01	1.3936E+02	8.2386E+01	1.1831E+03	9.1414E+01	7.4456E+01
	Rank	1	4	6	5	7	2	3
F24	Best	3.6855E+03	5.5811E+03	7.1098E+03	6.0777E+03	4.1517E+03	3.8757E+03	3.9996E+03
	Mean	3.7750E+03	6.9432E+03	1.0489E+04	6.7266E+03	4.5363E+03	4.1478E+03	4.3253E+03
	Std	6.6123E+01	5.8063E+02	1.7705E+03	4.3405E+02	2.5074E+02	2.2139E+02	2.0282E+02
	Rank	1	6	7	5	4	2	3
F25	Best	1.0327E+04	1.5205E+04	1.7354E+04	1.5855E+04	6.9212E+03	1.2156E+04	1.2600E+04
	Mean	1.2120E+04	2.0587E+04	2.0276E+04	1.7151E+04	1.6894E+04	1.4350E+04	1.4023E+04
	Std	1.1013E+03	3.1495E+03	1.4051E+03	7.3193E+02	3.8529E+03	1.0778E+03	7.8640E+02
	Rank	1	7	6	5	4	3	2
F26	Best	3.7547E+03	3.7500E+03	4.1491E+03	3.7030E+03	3.2000E+03	3.7114E+03	3.6303E+03
	Mean	3.8663E+03	3.9459E+03	4.4170E+03	3.9933E+03	3.7067E+03	3.8971E+03	3.8426E+03
	Std	5.7117E+01	1.1816E+02	1.7142E+02	9.5782E+01	3.6265E+02	1.3734E+02	9.3087E+01
	Rank	3	5	7	6	1	4	2
F27	Best	3.8831E+03	7.3664E+03	9.7387E+03	6.6589E+03	4.4132E+03	3.9053E+03	4.4837E+03
	Mean	4.0196E+03	9.4479E+03	1.3978E+04	8.3477E+03	4.8711E+03	4.8804E+03	5.3844E+03
	Std	8.2876E+01	9.5457E+02	2.2875E+03	7.7473E+02	3.0214E+02	6.1081E+02	7.3620E+02
	Rank	1	6	7	5	2	3	4
F28	Best	6.1746E+03	7.2910E+03	8.1055E+03	8.0273E+03	7.8375E+03	7.5730E+03	6.8076E+03
	Mean	6.9884E+03	8.8324E+03	9.1358E+03	9.0762E+03	8.8764E+03	8.5958E+03	8.1343E+03
	Std	4.4857E+02	5.3314E+02	6.0512E+02	7.3307E+02	5.6548E+02	6.4556E+02	6.6291E+02
	Rank	1	4	7	6	5	3	2
F29	Best	9.3543E+06	5.3418E+07	1.1807E+08	8.7870E+07	6.7614E+07	5.5284E+07	3.3540E+07
	Mean	2.9735E+07	2.1424E+08	2.4597E+08	1.7817E+08	1.7591E+08	1.9984E+08	1.0431E+08
	Std	1.0504E+07	7.7033E+07	8.4721E+07	5.5652E+07	6.9471E+07	9.0530E+07	5.2053E+07
	Rank	1	6	7	4	3	5	2

**Table A9:** Comparison of DRIME with other improved algorithms in CEC-2022 test suite (D = 10).

No.	Index	DRIME	EOSMA	GLS-MPA	ISGTOA	EMTLBO	LSHADE-SPACMA	APSM-jSO
F1	Best	3.0002E+02	1.2467E+03	3.9630E+02	7.3559E+02	1.2638E+03	3.1911E+02	3.1975E+02
	Mean	3.0020E+02	2.9857E+03	1.2092E+03	1.9775E+03	3.9370E+03	4.4028E+02	3.7410E+02
	Std	1.4672E-01	1.1523E+03	6.0770E+02	8.2658E+02	1.4037E+03	2.2402E+02	4.3890E+01
	Rank	1	6	4	5	7	3	2
F2	Best	4.0000E+02	4.0577E+02	4.0079E+02	4.0025E+02	4.0175E+02	4.0558E+02	4.0073E+02
	Mean	4.0057E+02	4.1951E+02	4.1741E+02	4.0942E+02	4.2635E+02	4.0775E+02	4.0761E+02
	Std	1.8908E+00	1.3433E+01	2.2760E+01	1.1371E+01	1.9773E+01	1.6658E+00	3.0934E+00
	Rank	1	6	5	4	7	3	2
F3	Best	6.0001E+02	6.0197E+02	6.0122E+02	6.0008E+02	6.0725E+02	6.0146E+02	6.0148E+02
	Mean	6.0008E+02	6.0396E+02	6.0664E+02	6.0023E+02	6.1248E+02	6.0241E+02	6.0256E+02
	Std	4.5623E-02	1.4518E+00	3.7382E+00	1.4080E-01	2.6019E+00	4.9018E-01	6.4149E-01
	Rank	1	5	6	2	7	3	4
F4	Best	8.0100E+02	8.0817E+02	8.0709E+02	8.0617E+02	8.2802E+02	8.2406E+02	8.2296E+02
	Mean	8.0631E+02	8.2974E+02	8.1867E+02	8.2653E+02	8.4735E+02	8.3729E+02	8.4367E+02
	Std	3.2727E+00	7.8692E+00	6.8865E+00	9.2396E+00	7.3932E+00	5.7350E+00	7.1487E+00
	Rank	1	4	2	3	7	5	6
F5	Best	9.0000E+02	9.0149E+02	9.0101E+02	9.0000E+02	9.0860E+02	9.0125E+02	9.0137E+02
	Mean	9.0004E+02	9.0830E+02	9.3457E+02	9.0008E+02	9.2118E+02	9.0330E+02	9.0342E+02
	Std	1.1728E-01	4.3773E+00	2.1704E+01	1.4355E-01	8.8962E+00	1.3681E+00	1.7665E+00
	Rank	1	5	7	2	6	3	4
F6	Best	1.8067E+03	6.7346E+03	1.8910E+03	2.9768E+03	1.8892E+03	2.0447E+03	2.2923E+03
	Mean	1.8499E+03	7.1976E+04	3.6534E+03	6.4743E+03	3.6396E+03	3.0700E+03	3.9545E+03
	Std	4.7378E+01	8.6235E+04	1.8236E+03	3.0557E+03	1.8423E+03	9.8815E+02	1.2744E+03
	Rank	1	7	4	6	3	2	5
F7	Best	2.0018E+03	2.0202E+03	2.0054E+03	2.0093E+03	2.0368E+03	2.0332E+03	2.0374E+03
	Mean	2.0173E+03	2.0388E+03	2.0270E+03	2.0355E+03	2.0617E+03	2.0425E+03	2.0514E+03
	Std	8.4174E+00	7.7721E+00	1.0293E+01	8.3766E+00	9.3100E+00	4.5166E+00	7.5242E+00
	Rank	1	4	2	3	7	5	6
F8	Best	2.2051E+03	2.2247E+03	2.2089E+03	2.2131E+03	2.2251E+03	2.2244E+03	2.2252E+03
	Mean	2.2222E+03	2.2309E+03	2.2231E+03	2.2290E+03	2.2320E+03	2.2287E+03	2.2309E+03
	Std	4.5423E+00	2.5177E+00	2.9881E+00	3.6336E+00	3.3632E+00	1.7950E+00	2.7025E+00
	Rank	1	5	2	4	7	3	6
F9	Best	2.5293E+03	2.5315E+03	2.5293E+03	2.5293E+03	2.5312E+03	2.5293E+03	2.5295E+03
	Mean	2.5294E+03	2.5423E+03	2.5309E+03	2.5293E+03	2.5360E+03	2.5294E+03	2.5299E+03
	Std	9.4325E-02	5.9188E+00	2.9474E+00	2.2059E-02	3.1567E+00	3.4485E-02	3.4836E-01
	Rank	3	7	5	1	6	2	4
F10	Best	2.5002E+03	2.5004E+03	2.5003E+03	2.5004E+03	2.5006E+03	2.5004E+03	2.5004E+03
	Mean	2.5040E+03	2.5006E+03	2.5007E+03	2.5132E+03	2.5010E+03	2.5006E+03	2.5156E+03
	Std	2.0065E+01	1.2838E-01	1.9641E-01	3.8620E+01	8.2727E-01	1.0298E-01	4.5473E+01
	Rank	5	2	3	6	4	1	7
F11	Best	2.6251E+03	2.7581E+03	2.7292E+03	2.6081E+03	2.7603E+03	2.7310E+03	2.7752E+03
	Mean	2.9024E+03	3.0631E+03	2.8483E+03	2.7669E+03	2.8703E+03	2.7778E+03	3.0244E+03
	Std	5.2819E+01	2.0215E+02	1.8146E+02	1.3738E+02	1.7731E+02	6.8433E+01	5.9996E+01
	Rank	5	7	3	1	4	2	6
F12	Best	2.8627E+03	2.8656E+03	2.8614E+03	2.8587E+03	2.8640E+03	2.8630E+03	2.8642E+03
	Mean	2.8654E+03	2.8670E+03	2.8642E+03	2.8633E+03	2.8661E+03	2.8646E+03	2.8652E+03
	Std	1.0279E+00	1.2031E+00	1.4228E+00	1.4756E+00	1.2038E+00	6.2745E-01	4.7693E-01
	Rank	5	7	2	1	6	3	4

**Table A10:** Comparison of DRIME with other improved algorithms in CEC-2022 test suite (D = 20).

No.	Index	DRIME	EOSMA	GLS-MPA	ISGTOA	EMTLBO	LSHADE-SPACMA	APSM-jSO
F1	Best	3.0497E+02	8.7616E+03	4.0382E+03	7.3815E+03	1.1842E+04	4.1624E+02	8.4551E+02
	Mean	3.3503E+02	1.5822E+04	1.0588E+04	1.4211E+04	2.2919E+04	2.0665E+03	2.6063E+03
	Std	2.8664E+01	4.3551E+03	3.2946E+03	4.3340E+03	7.9294E+03	4.6981E+03	1.5004E+03
	Rank	1	6	4	5	7	2	3
F2	Best	4.4950E+02	4.7153E+02	4.7263E+02	4.3177E+02	4.7459E+02	4.4515E+02	4.4991E+02
	Mean	4.5783E+02	5.0605E+02	5.2247E+02	4.6044E+02	5.2255E+02	4.4842E+02	4.5173E+02
	Std	8.4541E+00	2.6998E+01	3.8051E+01	1.4800E+01	4.3122E+01	1.4802E+00	1.9822E+00
	Rank	3	5	6	4	7	1	2
F3	Best	6.0008E+02	6.0474E+02	6.0967E+02	6.0034E+02	6.1328E+02	6.0148E+02	6.0242E+02
	Mean	6.0043E+02	6.0972E+02	6.1928E+02	6.0135E+02	6.2089E+02	6.0202E+02	6.0367E+02
	Std	4.6460E-01	3.4019E+00	5.2551E+00	7.1706E-01	4.4356E+00	3.0820E-01	8.8521E-01
	Rank	1	5	6	2	7	3	4
F4	Best	8.0722E+02	8.6308E+02	8.3813E+02	8.3881E+02	9.1778E+02	8.8861E+02	8.9409E+02
	Mean	8.2054E+02	8.9232E+02	8.7323E+02	8.9698E+02	9.3338E+02	9.0833E+02	9.1923E+02
	Std	1.1572E+01	1.1936E+01	1.7386E+01	2.5461E+01	8.9492E+00	8.0436E+00	1.0812E+01
	Rank	1	3	2	4	7	5	6
F5	Best	9.0001E+02	9.3501E+02	1.0361E+03	9.0039E+02	9.6077E+02	9.0198E+02	9.0326E+02
	Mean	9.0052E+02	1.0330E+03	1.4148E+03	9.0802E+02	1.0551E+03	9.0478E+02	9.0985E+02
	Std	7.9534E-01	7.0684E+01	2.5375E+02	1.3174E+01	7.1880E+01	1.1807E+00	5.9775E+00
	Rank	1	5	7	3	6	2	4
F6	Best	1.9620E+03	7.3205E+04	2.4298E+03	2.2454E+03	5.4226E+03	9.5935E+03	4.1953E+04
	Mean	2.8321E+03	6.3594E+05	1.8336E+05	6.3993E+03	7.7360E+04	3.4160E+04	1.2375E+05
	Std	6.8932E+02	4.6314E+05	3.5938E+05	4.6565E+03	8.8667E+04	1.1985E+04	4.8172E+04
	Rank	1	7	6	2	4	3	5
F7	Best	2.0272E+03	2.0610E+03	2.0449E+03	2.0578E+03	2.0969E+03	2.0919E+03	2.0943E+03
	Mean	2.0447E+03	2.0965E+03	2.0729E+03	2.0856E+03	2.1561E+03	2.1213E+03	2.1344E+03
	Std	1.1041E+01	1.8864E+01	1.9881E+01	1.6962E+01	2.4403E+01	1.2468E+01	2.2518E+01
	Rank	1	4	2	3	7	5	6
F8	Best	2.2244E+03	2.2310E+03	2.2232E+03	2.2322E+03	2.2330E+03	2.2366E+03	2.2356E+03
	Mean	2.2266E+03	2.2404E+03	2.2310E+03	2.2406E+03	2.2839E+03	2.2426E+03	2.2444E+03
	Std	1.2058E+00	5.5594E+00	3.8483E+00	5.0694E+00	6.6817E+01	3.2525E+00	4.4134E+00
	Rank	1	3	2	4	7	5	6
F9	Best	2.4818E+03	2.4908E+03	2.4833E+03	2.4809E+03	2.4903E+03	2.4808E+03	2.4813E+03
	Mean	2.4834E+03	2.4981E+03	2.4996E+03	2.4814E+03	2.5008E+03	2.4809E+03	2.4819E+03
	Std	1.0742E+00	4.8031E+00	1.2125E+01	4.4272E-01	7.1851E+00	1.9691E-02	4.2590E-01
	Rank	4	5	6	2	7	1	3
F10	Best	2.5003E+03	2.5007E+03	2.5006E+03	2.5005E+03	2.5007E+03	2.5005E+03	2.5007E+03
	Mean	2.5086E+03	2.5080E+03	2.5015E+03	2.8497E+03	2.5421E+03	2.5008E+03	2.5094E+03
	Std	3.1016E+01	3.7933E+01	1.5041E+00	1.0366E+03	1.2269E+02	1.4472E-01	4.4999E+01
	Rank	4	3	2	7	6	1	5
F11	Best	2.9533E+03	3.7787E+03	3.5594E+03	2.9155E+03	3.7200E+03	3.0197E+03	3.2743E+03
	Mean	3.0020E+03	4.1439E+03	4.2950E+03	2.9270E+03	4.1415E+03	3.0695E+03	3.5196E+03
	Std	4.0613E+01	2.0977E+02	4.0318E+02	1.0313E+01	2.0632E+02	2.6716E+01	1.3406E+02
	Rank	2	6	7	1	5	3	4
F12	Best	2.9405E+03	2.9691E+03	2.9492E+03	2.9380E+03	2.9561E+03	2.9365E+03	2.9465E+03
	Mean	2.9538E+03	3.0008E+03	2.9743E+03	2.9511E+03	2.9759E+03	2.9459E+03	2.9591E+03
	Std	7.2714E+00	1.6865E+01	1.6759E+01	1.1757E+01	1.7107E+01	5.1191E+00	7.3760E+00
	Rank	3	7	5	2	6	1	4
